# A Modified Murine Embryonic Stem Cell Test for Evaluating the Teratogenic Effects of Drugs on Early Embryogenesis

**DOI:** 10.1371/journal.pone.0145286

**Published:** 2015-12-18

**Authors:** Ruoxing Yu, Norio Miyamura, Yoshimi Okamoto-Uchida, Norie Arima, Mari Ishigami-Yuasa, Hiroyuki Kagechika, Hiroshi Nishina

**Affiliations:** 1 Department of Developmental and Regenerative Biology, Medical Research Institute (MRI), Tokyo Medical and Dental University (TMDU), Tokyo, 113–8510, Japan; 2 Chemical Biology Screening Center, and Department of Organic and Medicinal Chemistry, Institute of Biomaterials and Bioengineering, TMDU, Tokyo, 101–0062, Japan; University of Newcastle upon Tyne, UNITED KINGDOM

## Abstract

Mammalian fetal development is easily disrupted by exogenous agents, making it essential to test new drug candidates for embryotoxicity and teratogenicity. To standardize the testing of drugs that might be used to treat pregnant women, the U.S. Food and Drug Administration (FDA) formulated special grade categories, labeled A, B, C, D and X, that define the level of risk associated with the use of a specific drug during pregnancy. Drugs in categories (Cat.) D and X are those with embryotoxic and/or teratogenic effects on humans and animals. However, which stages of pregnancy are affected by these agents and their molecular mechanisms are unknown. We describe here an embryonic stem cell test (EST) that classifies FDA pregnancy Cat.D and Cat.X drugs into 4 classes based on their differing effects on primitive streak formation. We show that ~84% of Cat.D and Cat.X drugs target this period of embryogenesis. Our results demonstrate that our modified EST can identify how a drug affects early embryogenesis, when it acts, and its molecular mechanism. Our test may thus be a useful addition to the drug safety testing armamentarium.

## Introduction

Although drug use during pregnancy is a major cause of teratogenicity and embryotoxicity, the demand for drugs to treat gestational side-effects or pre-existing medical conditions during pregnancy is increasing every year. The disaster caused by thalidomide use to treat morning sickness in the 1960s highlighted the need to consider the teratogenicity of prescribed drugs more carefully [1][2]. In 1979, to standardize the testing of drugs for their effects during pregnancy, the United States Food and Drug Administration (FDA) formulated special grade categories (Cat.), labeled A, B, C, D and X, that define the level of risk associated with the use of a particular drug during pregnancy [3]. Each category was defined by the presence or absence of data on teratogenicity/embryotoxicity, the source of the data (animal and/or human studies), and the results of these studies (positive or negative findings) [3]. While clinicians generally agree that drugs in Cat.A and Cat.B are relatively safe, the majority of medications are classified in the more nebulous Cat.C, reflecting the lack of available risk data for most drugs [4]. For Cat.D and Cat.X drugs, data indicating a potential risk to the fetus have been acquired from both human samples and animal models, and clinicians generally agree that the use of Cat.D and Cat.X drugs during pregnancy should be limited. However, the actual teratogenic/embryotoxic effects of these agents during early embryogenesis are unknown. In addition, the above FDA categories are designed to guide drug choice before fetal exposure, rather than provide information on how to manage a pregnancy following exposure [5].

*In vitro* embryonic stem cell tests (EST) using mouse or human ES cells are currently in wide use for assessing the embryotoxic potential of chemical compounds [6][7][8][9][10]. Using the hanging drop method, embryoid bodies (EBs) with the potential to differentiate into the three fundamental germ layers can be formed and cultured for several days. To measure the cytotoxicity of a given compound, EBs are cultured with scaled doses of the drug and its LC50 (level at which 50% of EB bodies are dead) is determined. Similarly, the ID50 (level of drug resulting in 50% inhibition of terminal differentiation) is determined by counting the number of beating cardiomyocyte foci in EBs. These criteria have been used to separate compounds into 3 groups: non-embryotoxic, weakly embryotoxic, and strongly embryotoxic. However, no information about the embryonic stage affected or the molecular mechanism of a given drug has been obtained to date.

Previous studies of mouse ES cells in our laboratory have revealed that some types of chemical compounds can interfere with cell fate determination, switching differentiating ES cells from becoming cardiomyocytes to becoming neurites [11][12]. We observed a similar effect, that of inhibition of cardiomyocyte beating and induction of neurogenesis, when primitive streak formation was blocked by an acetylcholine receptor antagonist [12]. Only after the initial primitive streak is established in a blastocyst arising from a fertilized egg can formation of the fundamental three germ layers begin. Cells of the initial primitive streak migrate bilaterally and anteriorly, giving rise to the anterolateral endoderm and mesoderm, with ectoderm arising [13]. Because primitive streak formation underlies germ layer differentiation, this process is meticulously regulated. Our previous results prompted us to develop an EST that could separate drugs based on their effects on primitive streak formation. Here we report our evaluation of the teratogenicity/embryotoxicity of 166 Cat.D and Cat.X drugs as determined by our new EST. Our classification data may provide clinically useful information about the stages of pregnancy affected by these drugs, their mechanisms, and their potential teratogenicity.

## Results

### Suitability of EB differentiation as a drug screening platform

As noted above, primitive streak formation is the basic foundation of germ layer development in the mammalian embryo. This stage of embryogenesis is short in duration but particularly sensitive to teratogens [14]. We speculated that treating EBs, which have been shown to reliably mimic early embryogenesis, with a drug and monitoring its effects on primitive streak formation might allow assessment of its potential teratogenicity/embryotoxicity.

**Fig 1:**
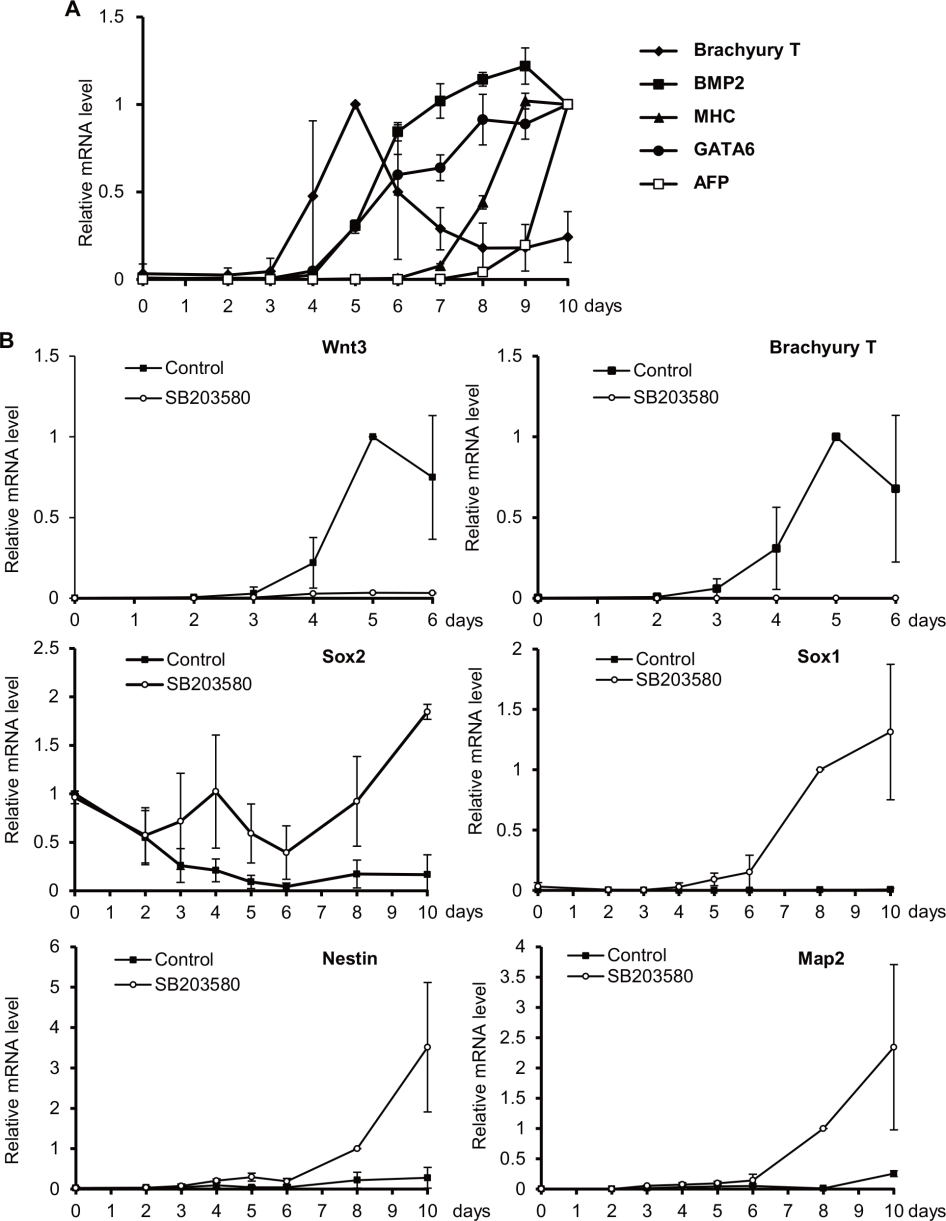
Analysis of developmental marker expression during EB culture. (A) Quantitative RT-PCR determination of relative mRNA levels of the primitive streak marker Brachyury T, mesoderm marker BMP2, endoderm marker GATA6, cardiomyogenesis marker MHC, and hepatogenesis marker AFP in untreated EBs cultured for the indicated times. Data are expressed relative to GAPDH expression and are the mean ± SEM (>30 EBs/group). (B) Relative mRNA levels for the indicated genes in EBs that were treated with DMSO or SB203580 on days 3–6 and cultured for the indicated times. Sox2, ectoderm marker; Nestin, Sox1 and Map2, neuroectoderm markers. Data were analyzed as for Fig 1A and are representative of 3 independent experiments.

To establish a time course of gene expression during EB formation in our EST, we measured the mRNA expression of various developmental markers using real-time PCR. As expected, expression of the primitive streak marker Brachyury T commenced after 3 days of hanging drop culture, peaked on day 5, and then slowly declined after day 6 (Fig 1A). In contrast, expression of the mesoderm marker BMP2 started only after day 5, as did that of the cardiomyogenesis marker MHC. A similar trend occurred for the endoderm marker GATA6 and the hepatogenesis marker AFP (Fig 1A). These results demonstrated that primitive streak formation in our EST started after day 3 and was maintained through days 4 to 6, followed by mesoderm and endoderm differentiation. By day 10, “EB beating” could be visually detected in our system, indicating the presence of terminally differentiated somatic cells such as cardiomyocytes. These data confirm that early embryogenesis proceeds normally in our EST.

We previously reported that treatment of EBs with the p38 MAPK inhibitor SB203580 strongly induced the appearance of neurites at the expense of beating cardiomyocytes [11]. Other evidence has suggested that p38 MAPK is involved in the differentiation of mesoderm from the primitive streak [15]. To confirm this hypothesis, we treated EBs with SB203580 during days 3–6 and observed suppression of mRNA expression of the primitive streak markers Wnt3 and Brachyury T, but premature induction of the ectoderm marker Sox2 that commenced after day 3 and peaked at day 4 (Fig 1B). In contrast to their complete lack of expression in normal EBs, the neuroectoderm markers Sox1 and Nestin appeared after days 5 and 6, respectively, in SB203580-treated EBs, followed by expression of the neuron marker Map2 on days 9–10 (Fig 1B). These results suggested that SB203580-mediated inhibition of primitive streak formation triggers induction of ectoderm development and initial neurogenesis. As well as constituting fresh evidence for how early embryogenesis occurs in the EB system, these gene expression data prompted us to speculate that our EB system might provide a visual means of following drug-induced cell fate changes. Since this visualization would be very convenient for high-throughput screening, we set out to test if our EST could identify drugs with detrimental effects on primitive streak formation.

### EB differentiation testing identifies four classes of Cat.D and Cat.X drugs

**Fig 2:**
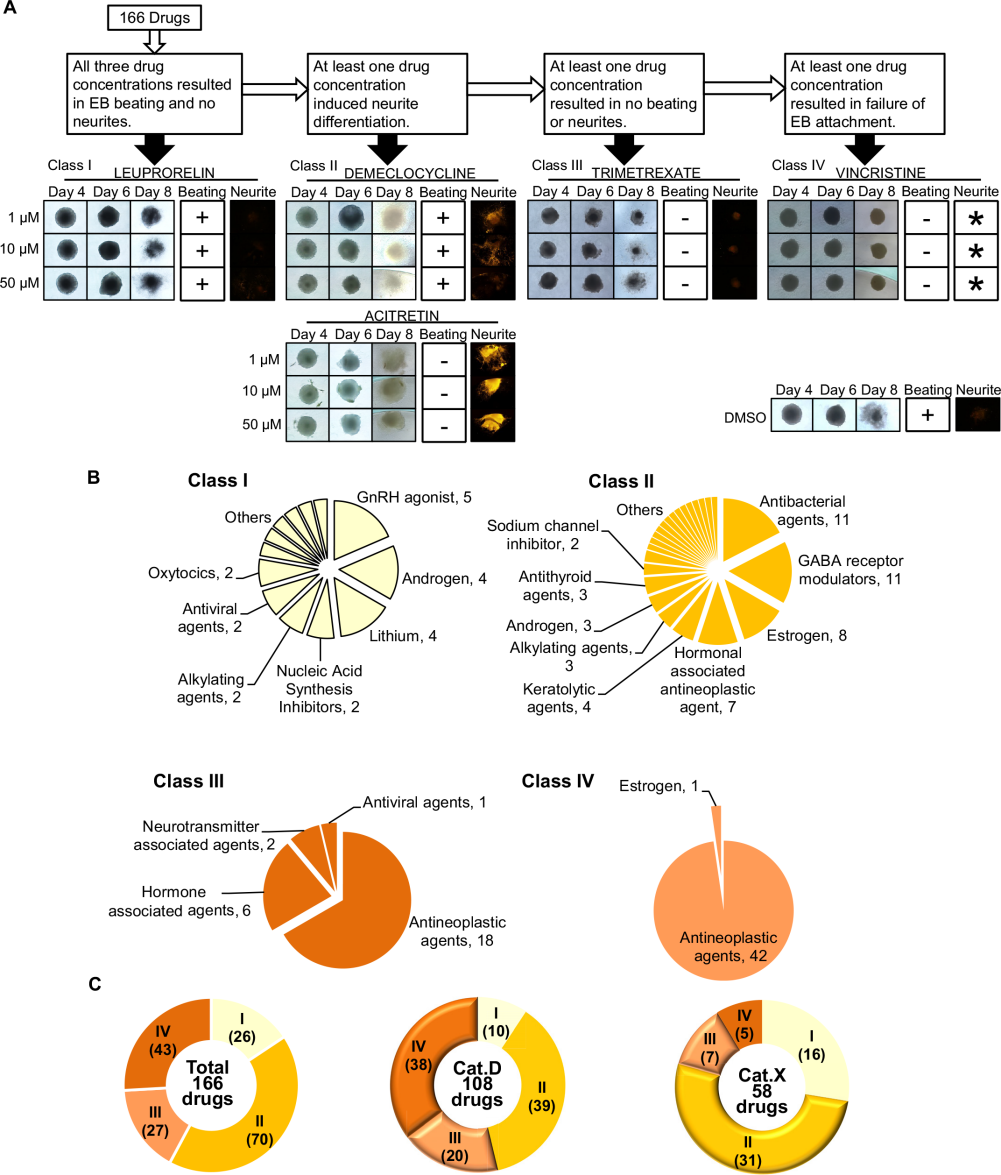
Classification of FDA pregnancy category D and X drugs into four classes of potential teratogens. (A) Scheme illustrating the growth and phenotypes of representative EBs treated with the indicated class-representative drugs for the indicated times of EB development. “Beating”, cardiomyocytes were present in the EB. “Neurite”, neuronal differentiation was observed. EBs treated with Class I drugs displayed beating and no neurites. Class II drug-treated EBs showed neurites with or without foci of beating cardiomyocytes. Class III drug-treated EBs showed neither beating nor neurites but the EBs survived. Class IV drugs resulted in dead EBs. -, EBs attached to the plate but no beating observed. *, data could not be obtained because dead EBs did not attach to the plate. Images of DMSO-treated control EBs at the same stages are also shown. (B) Pie charts of distribution of the indicated drug types among Classes I–IV. (C) Pie charts of numbers of total, Cat.D and Cat.X drugs in Classes I–IV.

To investigate the teratogenic effects of FDA-approved agents on early embryogenesis, we performed an EB differentiation screen of 166 drugs (S1 Table). Among these drugs, 108 belonged to Cat.D and 58 belonged to Cat.X. We treated mass culture plates of EBs with 3 concentrations (1 μM, 10 μM and 50 μM) of each drug during days 3–6 of culture, which is the period of primitive streak formation. On day 10, we counted numbers of beating foci in EBs and then stained the EBs with anti-β-tubulin III antibody (Ab) to detect neurites. Using 4 rounds of screening, we were able to separate the 166 drugs into 4 classes according to whether the drug-treated EBs displayed one or both or neither of these phenotypes (Fig 2A and S1 Fig). Class I drugs were those for which EBs treated with all 3 concentrations showed beating and no neurites, a phenotype comparable with that of DMSO-treated control EBs. No changes associated with differentiation or cell death were observed. Drugs in Class I included Gonadotropin-Releasing Hormone (GnRH) agonist, androgen-associated drugs, and lithium (Fig 2B). For Class II drugs, EBs treated with at least one concentration displayed neurites. Beating might or might not be present. Thus, Class II drugs, which included antibacterial agents, γ-aminobutyric acid (GABA) receptor modulators, and estrogen-associated drugs, might induce EBs to change cell fate. All Class III drugs inhibited EB cardiomyogenesis but had little or no effect on EB survival. No neurite differentiation was observed. Thus, Class III drugs, which included antiviral and some antineoplastic agents, might alter cell fate or occasionally induce cell death. In contrast, EBs treated with Class IV drugs could not grow and could not attach to a flat plate on day 8. Thus, Class IV drugs, which include many antineoplastic agents, are toxic to cells during the primitive streak period. Collation of these data revealed that, of the 166 drugs examined, 26 belonged to Class I, 70 belonged to Class II, 27 belonged to Class III, and 43 belonged to Class IV (Fig 2C). In total, about 84% of the 166 drugs tested caused EBs to show phenotypic differences from control EBs. For Cat.D drugs, 10 fell into Class I, 39 into Class II, 20 into Class III, and 38 into Class IV. For Cat.X drugs, there were 16 Class I drugs, 31 Class II drugs, 7 Class III drugs, and 5 Class IV drugs (Fig 2C). Examination of class distribution in each category showed that Cat.D drugs mainly fell into Class III or Class IV, meaning that they might induce cell death. For Cat.X drugs, most fell into Class II or Class III, meaning that they might alter cell fate. These results indicate that the majority of Cat.D and Cat.X drugs are likely to affect early embryogenesis during the primitive streak period, either by switching cell differentiation or inducing cell death. To confirm the validity of our system, we tested several Cat.A and Cat.B drugs, which are considered safe for use during pregnancy. None of these drugs had any effect on primitive streak formation (S3 Table). Thus, our EB system is indeed a sensitive means of assessing drug teratogenicity/embryotoxicity.

**Fig 3:**
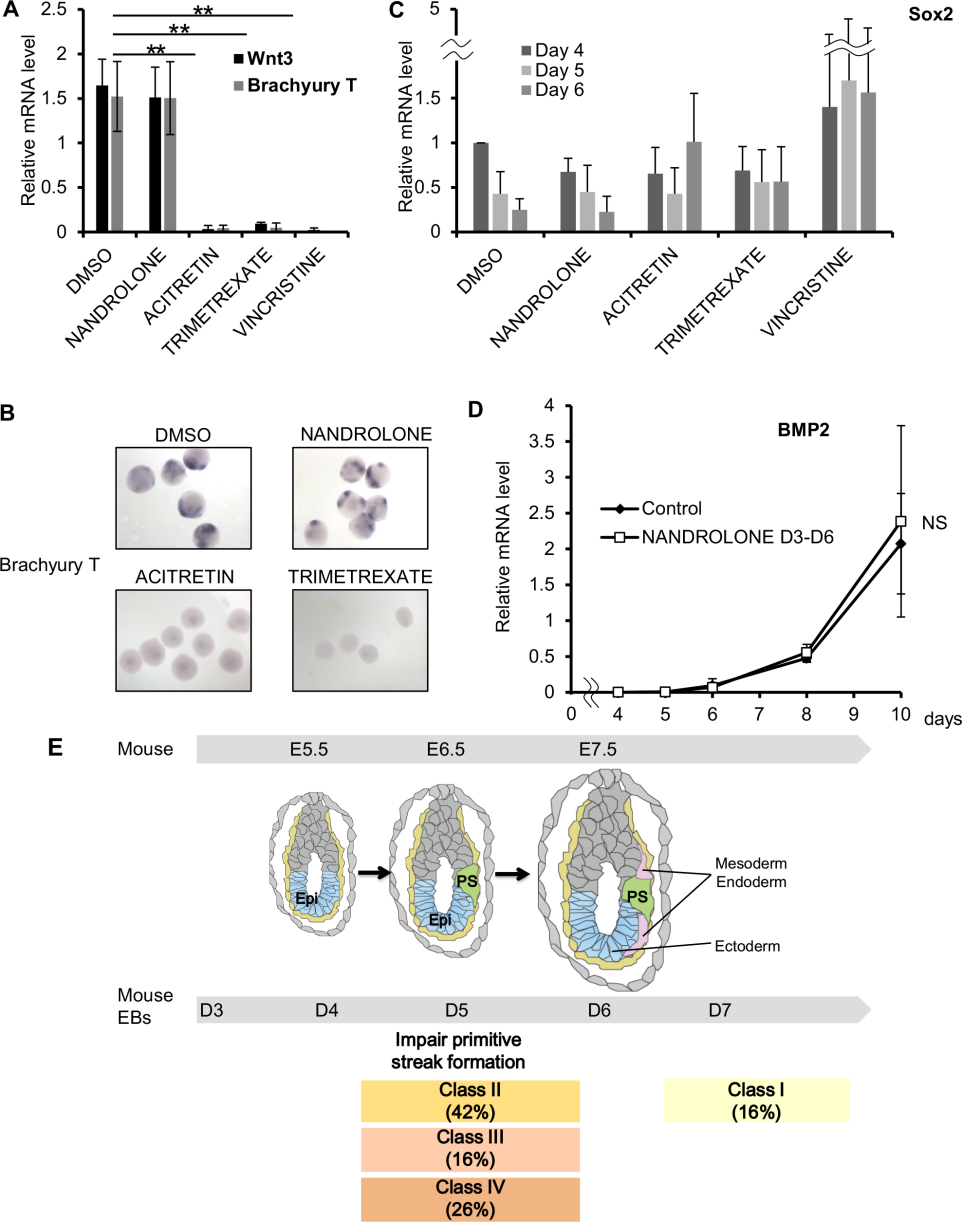
Effects of drug classes on EB gene expression patterns. (A-C) EBs were treated with the indicated class-representative drugs for days 3–6 of EB culture (>30 EB cultures/group). Samples were collected on days 4–6. DMSO, control; Nandrolone, Class I; acitretin, Class II; trimetrexate, Class III; vincristine, Class IV; SB203580, p38MAPK inhibitor. (A) Quantitative RT-PCR analysis of Wnt3 mRNA on day 6 determined as for Fig 1A. **, P<0.01. (B) *In situ* hybridization to detect Brachyury T on day 6 in EB cultures treated as indicated for days 3–6. Results are representative of 3 independent experiments (15 EBs/group). (C) Quantitative RT-PCR analysis of the indicated mRNAs on the indicated days determined as for Fig 1B. (D) Quantitative RT-PCR analysis of Bmp2 mRNA in EB cultures that were treated with DMSO (control) or nandrolone on days 3–6. Data were analyzed as for Fig 1A. NS, not significant. (E) Top: Illustration of mouse embryonic and germ layer development at the indicated embryonic days (E). Epi, epiblast; PS, primitive streak. Bottom: Illustration of days (D) of mouse EB culture indicating the percentage of each drug class acting at a particular stage of culture.

To further confirm the accuracy of our drug classification scheme based on effects on primitive streak formation, we chose one drug typical of each class and examined mRNA expression of relevant developmental markers. We evaluated: nandrolone, which is an anabolic steroid belonging to Class I; acitretin, which is a retinoic acid receptor agonist belonging to Class II; trimetrexate, which is a folic acid antagonist belonging to Class III; and vincristine, an antineoplastic agent belonging to Class IV. We found that mRNA levels of the primitive streak markers Wnt3 and Brachyury T in EB cultures were suppressed by acitretin, trimetrexate or vincristine treatment but not by nandrolone (Fig 3A). *In situ* hybridization using a probe for Brachyury T showed no significant changes in nandrolone treated-EBs compared to DMSO-treated controls, but lower levels of Brachyury T hybridization in EBs treated with acitretin or trimetrexate (Fig 3B). In addition, trimetrexate-treated EBs were smaller in size than controls. These data indicated that both acitretin and trimetrexate inhibit primitive streak formation, and that trimetrexate also induces cell death in EBs. EBs treated with vincristine were too severely damaged to maintain their structure during *in situ* hybridization, precluding a valid result. These data are in line with our drug classification system and support its utility.

To specifically determine the effect of each of our drug classes on ectoderm development, drug-treated EBs were collected on days 4–6 and levels of Sox2 mRNA were examined using SB203580 treatment as a positive control for suppression. We observed that Sox2 mRNA levels in DMSO-treated EBs were initially high but decreased with time, as they did in nandrolone-treated EBs (Fig 3C). Acitretin-treated EBs showed a decrease in Sox2 mRNA on day 5 but then an increase on day 6. Because Sox2 is a marker of undifferentiated cells, trimetrexate-treated EBs showed constant Sox2 expression. In vincristine-treated EBs, Sox2 levels appeared to change randomly. These results are also in line with our drug classification system, in that the Class I drug nandrolone had no effect on ectoderm development, while the Class II drug acitretin induced differentiation, and the Class III drug trimetrexate prevented differentiation. To further prove that the Class I drug nandrolone had no effect on primitive streak formation, we determined mRNA levels of the mesoderm marker Bmp2 on days 4–10 of EB culture. As expected, Bmp2 expression levels did not change with nandrolone treatment (Fig 3D), confirming that nandrolone does not prevent mesoderm differentiation.

Based on our data, we propose a model (Fig 3E) describing the murine developmental stages affected by our drug classes and the potential mechanisms of teratogenicity of these agents. Class II, III and IV drugs can impinge on primitive streak formation, either by altering cell differentiation or inducing cell death, and so are potentially teratogenic. In contrast, Class I drugs do not impair primitive streak formation and so are likely safe for use during early pregnancy.

### Evaluation of Class II drug function on early embryogenesis *in vivo*

**Fig 4:**
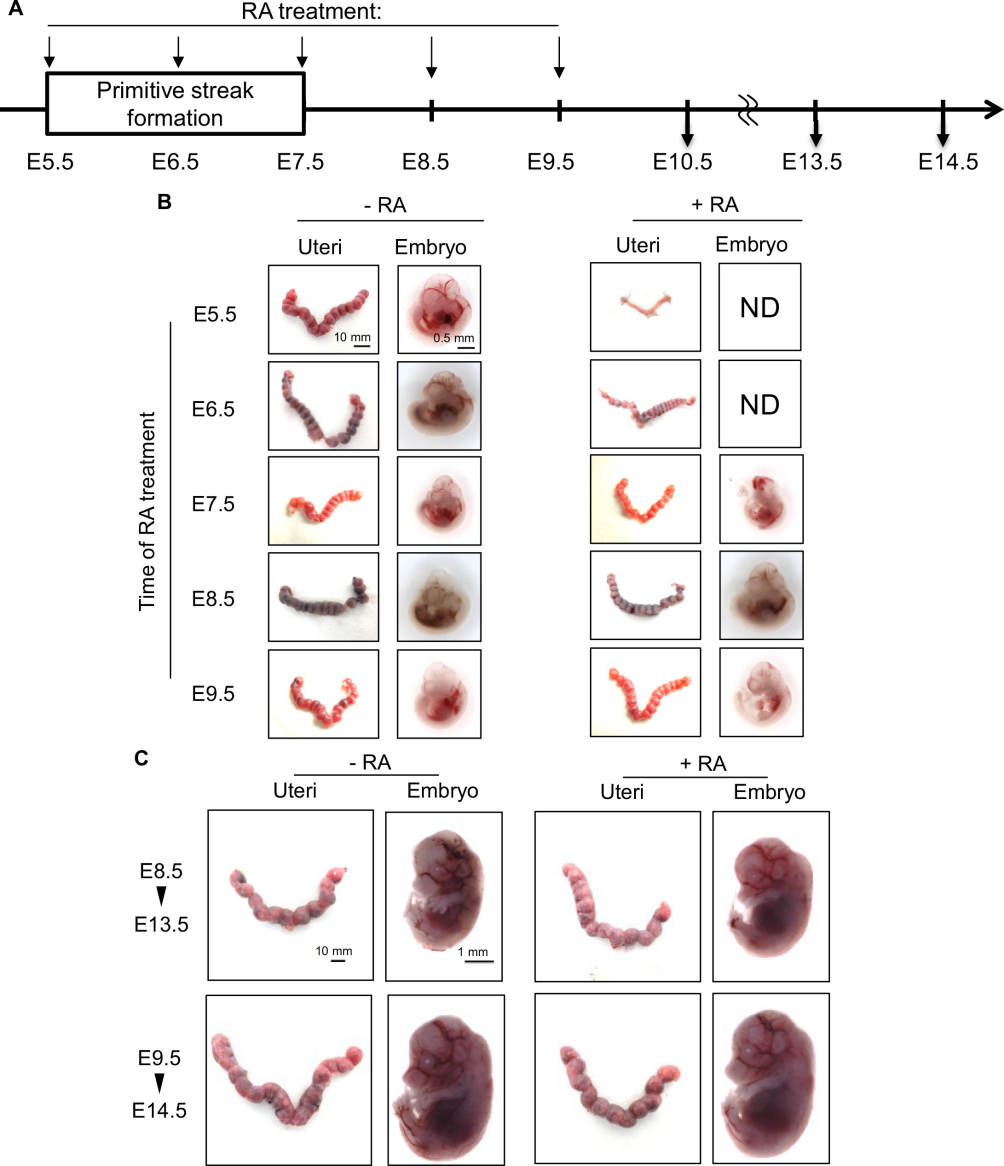
*In vivo* effects of retinoic acid on early mouse embryogenesis. (A) Scheme illustrating the experimental timeline during which pregnant mice received intravenous injection of 25 mg/kg RA at E5.5, E6.5, E7.5, E8.5 or E9.5. Mice were sacrificed on E10.5, E13.5 or E14.5 and macroscopic effects on uteri and embryo development were determined. (B) Representative images of morphological changes observed in embryos in the uteri of mice that were treated with RA (+) or not (-) at the indicated stages and examined at E10.5. ND, not detected. (C) Representative images of morphological changes observed in embryos in the uteri of mice that were treated with RA at E8.5 and examined at E13.5, or treated at E9.5 and examined at E14.5.

To obtain *in vivo* data validating our *in vitro* results acquired using EBs, we compared the effects of retinoic acid (RA) on EBs and early mouse embryos *in utero*. Our *in vitro* work showed that all four RA derivatives (tazarotene, acitretin, etretinate, isotretinoin) were Class II drugs with strong detrimental effects on primitive streak formation (S1 Fig). To investigate this effect *in vivo*, we injected RA into pregnant mice at E5.5, E6.5, E7.5, E8.5 or E9.5 via the tail vein and sacrificed the animals at E10.5, E13.5 or E14.5 (Fig 4A). All embryos in mice treated with RA at E5.5 and E6.5 were resorbed, and all those in mice treated with RA at E7.5 were morphologically abnormal (Fig 4B). However, embryos in mice treated with RA at E8.5 and E9.5 had normal morphology even when examined at E13.5 or E14.5 (Fig 4B and 4C). These results indicate that primitive streak formation is sensitive to RA *in vivo*, and confirm that our *in vitro* EST produces results consistent with *in vivo* outcomes.

### Dissection of the effects of benzodiazepines on early embryogenesis

**Fig 5:**
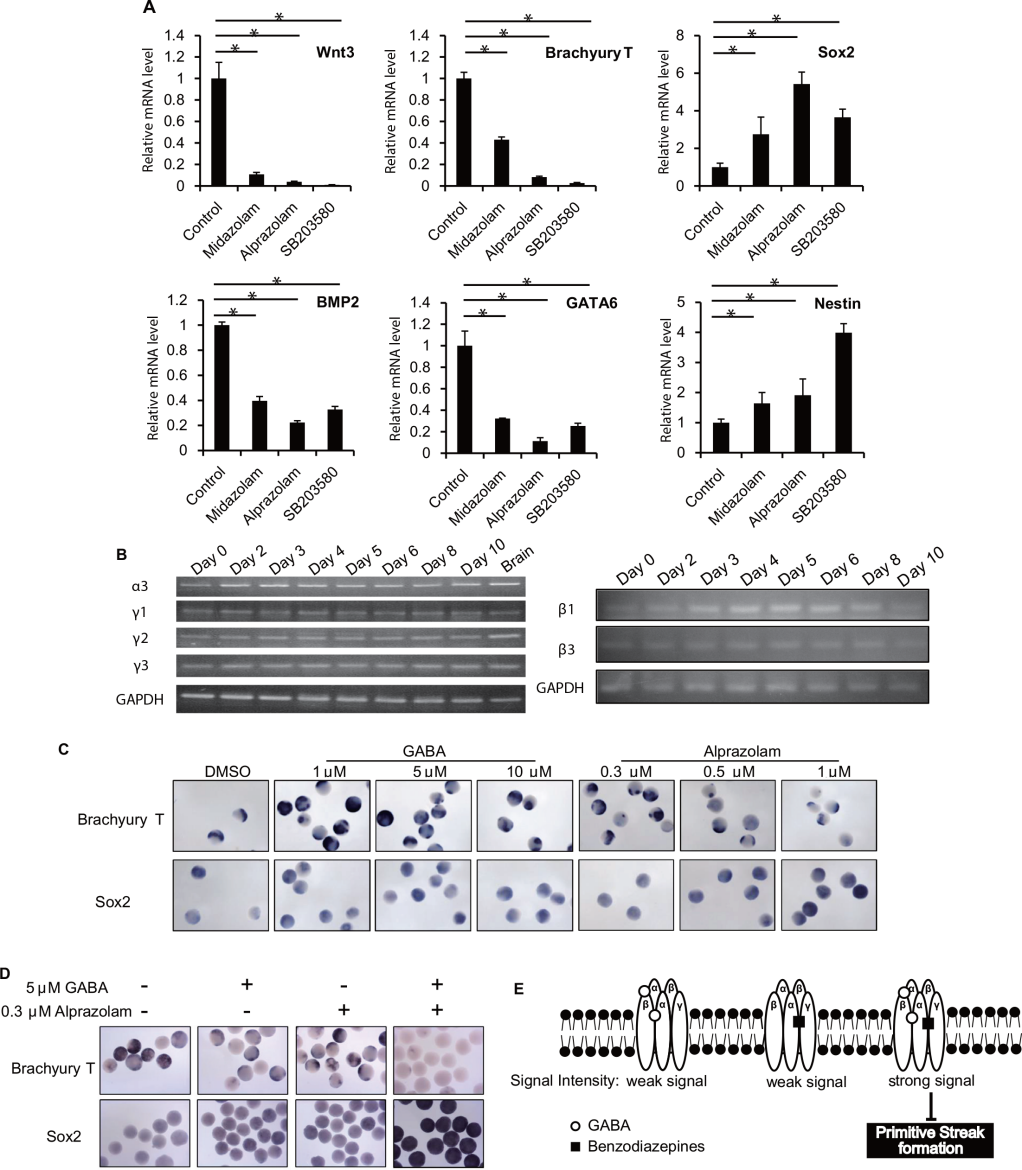
Teratogenicity of benzodiazepines. (A) Quantitative RT-PCR determination of mRNA levels of the indicated genes in EB cultures that were treated for days 3–6 with DMSO (control) or the indicated drugs (>30 EBs/group) and evaluated on day 6. Wnt3 and Brachyury T, primitive streak markers; Sox2, ectoderm marker; BMP2, mesoderm marker; GATA6, endoderm marker; Nestin, neuroectoderm marker. Data were analyzed as for Fig 1A. *, P<0.05. (B) RT-PCR analysis of expression of the indicated GABA receptor subunits in untreated EBs on the indicated days of culture. Data are representative of 3 independent experiments (>30 EBs/group). (C) *In situ* hybridization to detect Brachyury T and Sox2 in EBs on day 6 after GABA or alprazolam treatment at the indicated concentrations (15 EBs/group). (D) *In situ* hybridization to detect Brachyury T and Sox2 in EBs that were treated for 6 days with alprazolam and/or GABA as indicated (20 EBs/group). (E) A proposed model of how BZ may affect primitive streak formation. Treatment with GABA or BZ alone induces only weak signals that cannot inhibit primitive streak formation. However, a combination of BZ plus GABA works synergistically and may induce a strong signal that suppresses primitive streak formation, impairing embryogenesis.

In our EST-based drug classification system, Class II drugs can change cell fate because they are likely to inhibit primitive streak formation and then prematurely induce ectoderm differentiation (Fig 3A and 3B). A notorious teratogen and member of Class II is the benzodiazepine (BZ) family of drugs (S1 Fig). To examine the effects of BZs on primitive streak formation in more detail, we treated over 30 EBs/group with the BZs alprazolam or midazolam, and analyzed mRNA levels of various differentiation markers. Compared to DMSO-treated control EBs, BZ-treated EBs showed decreased levels of Wnt3 and Brachyury T mRNA expression but an increase in Sox2 mRNA (Fig 5A). Similarly, Bmp2 and GATA6 mRNA expression levels were reduced by BZs but Nestin mRNA was increased. These data confirm that not only is primitive streak formation inhibited by BZs but that ectoderm differentiation is prematurely induced, mesoderm and endoderm formation are inhibited, and neuroectoderm starts to develop. Thus, the mechanism underlying the teratogenicity of BZs may be their deleterious effects on primitive streak formation.

The molecular targets of BZs are GABA A-type (GABAA) receptors, which are mainly expressed on the surfaces of inhibitory synapses in the central nervous system. GABAA receptors are chloride ion channels usually constructed of two α, two β and one γ subunits [16][17]. To investigate whether GABAA receptor function is indeed important during primitive streak formation, we determined mRNA levels of various isoforms of GABAA receptor subunits in EBs at days 0–10 of development. We first examined GABAA receptor subunit isoforms and found that the mRNAs for these subunits were constantly expressed during days 3–6 of EB development, the period of primitive streak formation (Fig 5B). Secondly, to define whether the GABAA receptor ligand GABA had an effect on primitive streak formation similar to that of BZs, we treated EBs with GABA or alprazolam during days 3–6 and performed *in situ* hybridization on day 6 to detect expression of Brachyury T and Sox2. We found that GABA treatment did not inhibit Brachyury T expression even at a concentration of 10 μM, whereas alprazolam demonstrated dose-dependent inhibition of Brachyury T expression starting at 1 μM (Fig 5C).

BZs operate through a positive allosteric mechanism, and a previous report showed that treatment of BZ plus GABA *in vitro* had a synergistic effect on current induction [17]. To investigate the effects of such combined treatment on early embryogenesis, we treated EBs with 5 μM GABA plus 0.3 μM alprazolam and assayed Brachyury T and Sox2 expression by *in situ* hybridization. Neither alprazolam nor GABA treatment alone altered Brachyury T and Sox2 expression in this assay, but the combination treatment inhibited Brachyury T expression while inducing Sox2 (Fig 5D). These results indicate that GABA treatment enhances the inhibitory effects of BZs on primitive streak formation. A model illustrating a mechanism for this synergy is shown in Fig 5E. The binding of GABA or BZ alone to a GABA receptor does not induce a signal of sufficient intensity to inhibit primitive streak formation. However, a GABAA receptor that is simultaneously activated by both GABA and BZ at the appropriate concentrations may generate a signal strong enough to derail primitive streak formation, leading to potential teratogenicity/embryotoxicity.

## Discussion

This study classified FDA pregnancy Cat.D and Cat.X drugs into 4 classes according to their mechanisms and effects assayed using a novel EB-based EST. We also used our EB system to show that BZs, which are GABA receptor modulators, can inhibit primitive streak formation in a manner enhanced by GABA co-treatment. Our work demonstrates that our EST is useful for both high-throughput drug screening and investigating mechanisms of drug teratogenicity.

The p38 MAPK signaling pathway is involved in many cellular processes, including inflammation and cell differentiation, survival and death. During embryonic development, the p38 inhibitor SB203580 blocks both mesoderm and endoderm differentiation from the primitive streak [15][18]. We have shown that SB203580 treatment inhibits mRNA expression of the primitive streak markers Wnt3 and Brachyury T but induces premature expression of neurogenesis markers, implicating p38 MAPK in the initial stages of primitive streak formation in the EB system. Because SB203580 treatment resulted in a phenotypic switch from cardiomyocyte beating to neurite development, we hypothesized that drug-induced changes to cell differentiation could be an appropriate basis for high-throughput screening of drugs for teratogenicity/embryotoxicity. Previous ESTs examining the embryotoxic effects of chemicals have been based mainly on assessments of the morphology of beating cardiomyocytes or on cytotoxic effects on differentiating mouse cells in culture [7][10][19]. A more recent EST used a reporter gene assay to determine the teratogenicity of drugs by analyzing the expression of differentiation markers such as Lhx1, Goosecoid (GSC), Foxa2, Eomes and FGF8 in drug-treated mouse EBs [9]. In our EST, we detect not only the inhibitory effects of a drug on EB cell differentiation and cardiomyogenesis but also the induction of cell death or neurogenesis (Fig 2A). Moreover, our EST is easily performed, provides a visual translation of changes to cell fate, and yields additional information on the gestational stage at which a drug acts.

GnRH antagonists have been previously shown to decrease embryo implantation by reducing the invasiveness of cytotrophoblasts [20]. However, in our study, we found that GnRH antagonists belonged to Class I, meaning that their effects on primitive streak formation and EB survival were minimal. Hence, our results suggest that GnRH antagonists exert their inhibitory effects on a much later stage of embryogenesis or other process during pregnancy. Our Class II drugs included various antibacterial agents and GABA receptor modulators. Tetracyclines are one of the most widely used types of antibacterial drugs and are also infamous teratogens [21]. GABA receptor modulators, which include BZs, are often used to treat extreme anxiety and other mental illnesses. Taken at high doses, BZs can induce intrauterine growth retardation (IUGR), cleft lip, and facial features resembling those of Fetal Alcohol Syndrome (FAS) [22]. In our study, all BZs tested belonged to Class II (Fig 2B), indicating that their teratogenic mechanism may stem from their inhibition of primitive streak formation.

Our Class III and Class IV drugs, which included antineoplastic agents, induced cell death. A previous comparison of mouse and human studies of the effects of embryonic exposure to the folic acid antagonist methotrexate revealed a significant species difference. While no mouse studies showed an increase in malformations at methotrexate dose levels of <20 mg/kg, human case reports indicated that methotrexate exposure induced severe teratogenicity unless used at a concentration of <10 mg/week [23]. In our study, all three folic acid antagonists tested fell into Class III, meaning that they induced a lower level of cell death during primitive streak formation than Class IV drugs. This evidence indicates that the teratogenic effects of folic acid antagonists are likely stage- and concentration-specific. Our Class IV drugs included many types of highly embryotoxic agents, such as the tubulin modulator vincristine. In mice, vincristine has severe embryotoxic and teratogenic activity that peaks on day 7 after blastocyst treatment [24]. In our EST, vincristine was highly cytotoxic to EBs even at <1 μM, and other tubulin modulators showed the same phenotype (S1 Fig). These results confirm that most anticancer drugs are toxic to undifferentiated cells and can lead to embryotoxicity and teratogenicity.

Both in the United States and in many other countries, the FDA special grade category is the most widely used tool for evaluating drug safety during pregnancy [4]. However, this scheme determines whether a drug can be used during pregnancy based on case reports with limited information and in the absence of a safety monitoring mechanism [3][25]. In our study, we used our novel EST to evaluate 166 Cat.D and Cat.X drugs, which are suspected teratogens. Among these drugs, ~84% fell into Class II, Class III or Class IV, meaning that they act during the primitive streak period and affect cell differentiation or induce cell death (Fig 3A–3D). Our EST data were corroborated by our analysis of the effects on embryos of treatment of pregnant mice with a Class II drug (Fig 4), confirming that our *in vitro* EST results are consistent with *in vivo* outcomes. Thus, our drug classification system provides information on when and how these agents work as teratogens, knowledge that should be highly useful for clinicians considering drug prescriptions for their pregnant patients. In addition, our results indicate that our EST has the potential to become a reliable tool for screening new drugs for possible teratogenicity by evaluating whether they target primitive streak formation.

Our findings also shed light on the ongoing controversy over whether BZs are teratogenic. The incidence of BZ use during pregnancy is unclear and is estimated to vary from 1–3% to 40% of all pregnant women in different populations [26]. There have been several cases of BZ teratogenicity reported [27][28], but other studies have found no evidence of ill effects of BZ use on the fetus [29][30]. In our study, BZs had their greatest effects on primitive streak formation when used in combination with GABA (Fig 5A–5D). Thus, the GABA concentration in a given embryo, which would depend on its embryonic stage and/or microenvironment, might determine the degree of BZ teratogenicity and result in conflicting data in different studies. Our EST efficiently revealed this potential molecular mechanism of embryonic damage, making our test even more suitable for high-throughput screening of new drugs for teratogenicity/embryotoxicity.

## Materials and Methods

### Cell culture

Feeder cell-independent E14K murine ES cells were cultured on gelatin-coated dishes in Dulbecco’s modified Eagle’s medium (Gibco) containing 15% bovine calf serum (BSC, Equitech-Bio), 0.1% 2-mercaptoethanol (Sigma), and 1000 U/ml leukemia inhibitory factor (LIF, propagation medium), as described previously [11][12] [31].

### Teratogen classification

All drugs chosen from FDA pregnancy categories D and X were supplied as 10 mM DMSO stock solutions by the Chemical Biology Screening Center of Tokyo Medical and Dental University. Suspension cultures of ES cells (3000 cells/well) were plated in low cell adhesion 96-well plates (PrimeSurface-U, Sumitomo Bakelite Co., Ltd.) and allowed to form EBs. For drug screening, EB cultures were treated from days 3 to 6 with DMSO (control) or 1 μM, 10 μM or 50 μM of each drug. On day 6, treated EBs were transferred to gelatin-coated plastic plates and cultured until day 10. Cardiomyogenesis was evaluated by counting beating foci under a light microscope on day 10. EBs were then stained with anti-β-tubulin III Ab to assess neuronal differentiation (see below).

### Immunofluorescence staining

As described previously [12], EBs on day 10 were fixed in 4% paraformaldehyde (PFA)/phosphate-buffered saline (PBS) for 2 h at 4°C, followed by washing with PBS. After preincubation with blocking solution (5% bovine serum albumin/PBS/0.1% Triton-X) for 1 h, EBs were incubated overnight at 4°C with a 1:1000 dilution of anti-β-tubulin III (Tuj-1, Covance) as the primary Ab. After washing with PBS/0.1% Triton-X, EBs were incubated for 1 h at room temperature (RT) with a 1:1000 dilution of Cy3-conjugated secondary Abs (ThermoFisher), 5 μM AlexaFluor488-conjugated phalloidin (Invitrogen), and 1 μg/ml Hoechst 33342 (Molecular Probes), followed by washing with PBS/0.1% Triton-X.

### Reverse transcriptase-polymerase chain reaction (PCR) and real-time PCR analysis

RNA isolation and RT-PCR were performed as previously described [12]. Briefly, ES cells or EBs were collected and immediately suspended in Tri Reagent (Molecular Research Center), and RNA was extracted according to the manufacturer’s instructions. Total RNA (2 μg) and oligo-d(T) primers (500 ng; see S2 Table for sequences) were used to synthesize cDNAs at 42°C for 90 min using Superscript III RNase H reverse transcriptase (Invitrogen) according to the manufacturer’s instructions. Quantitative real-time RT-PCR reactions were performed using the Chromo4 real-time detection system (Bio-Rad).

### Whole mount *in situ* hybridization

Whole mount *in situ* hybridization was performed as previously described [32] using antisense DIG-labeled probes and primers (see S2 Table for sequences).

### Statement of ethical animal experimentation

All procedures were performed in accordance with a protocol approved by the Tokyo Medical and Dental University Animal Care Committee (Permit Number: 0160227C2). All experiments were conducted so as to minimize pain and discomfort.

### Mice

Pregnant ICR mice (Nihon SLC) were reared under the normal 12 h light/dark cycle. The day of insemination was designated as embryonic day (E) 0. Pregnant female mice received 25 mg/kg of all-trans RA (Sigma) via intravenous tail vein injection at E5.5, E6.5, E7.5, E8.5 or E9.5. Control mice were injected with 100 μl DMSO (Sigma). At E10.5, E13.5 or E14.5, injected mice were sacrificed and uteri and embryos were analyzed for morphological changes under a light microscope.

### Statistical analyses

At least 3 independent trials were conducted of all experiments, which involved at least three independent experimental replicates per data point. Data are presented as the mean ± standard deviation. Statistical significance was determined using the Student’s t-test. Values of P<0.05 were considered to be statistically significant.

## Supporting Information

S1 Fig Scheme and raw data for the classification of Cat.D and Cat.X drugs in S1 Table. Mouse ES cells were plated in U-type 98 well plates and EBs were allowed to form. After 2 days in culture, EBs were treated with 3 concentrations of each FDA pregnancy Cat.D or Cat.X drug until day 6. Treated EBs were transferred to flat bottom 98 well plates and cultured until day 10 without drugs. Photographs of EBs under a light microscope were taken on days 4, 6 and 8. On day 10, EBs were visually evaluated for cardiomyocyte beating and immunostained to detect neurites.
(PDF)

S1 Methods Description of method used to test Cat.A and Cat.B drugs for teratogenicity and/or embryotoxicity using our modified EST. (DOCX)

S1 Table List of 166 FDA-approved Cat.D and Cat.X drugs screened for teratogenicity and/or embryotoxicity using our modified EST. (XLSX)

S2 Table List of primer sequences for gene expression studies. (XLSX)

S3 Table List of 15 FDA-approved Cat.A and Cat.B drugs screened for teratogenicity and/or embryotoxicity using our modified EST. (XLSX)
