# Supplementary material for: A Modified Murine Embryonic Stem Cell Test for Evaluating the Teratogenic Effects of Drugs on Early Embryogenesis
Source: PLoS One. 2015 Dec 18;10(12):e0145286. doi: 10.1371/journal.pone.0145286 (PMC4686177; doi:10.1371/journal.pone.0145286)
Supplement: S1 Fig — Mouse ES cells were plated in U-type 98 well plates and EBs were allowed to form. After 2 days in culture, EBs were treated with 3 concentrations of each FDA pregnancy Cat.D or Cat.X drug until day 6. Treated EBs were transferred to flat bottom 98 well plates and cultured until day 10 without drugs. Photographs of EBs under a light microscope were taken on days 4, 6 and 8. On day 10, EBs were visually evaluated for cardiomyocyte beating and immunostained to detect neurites. (PDF) [file pone.0145286.s001.pdf]

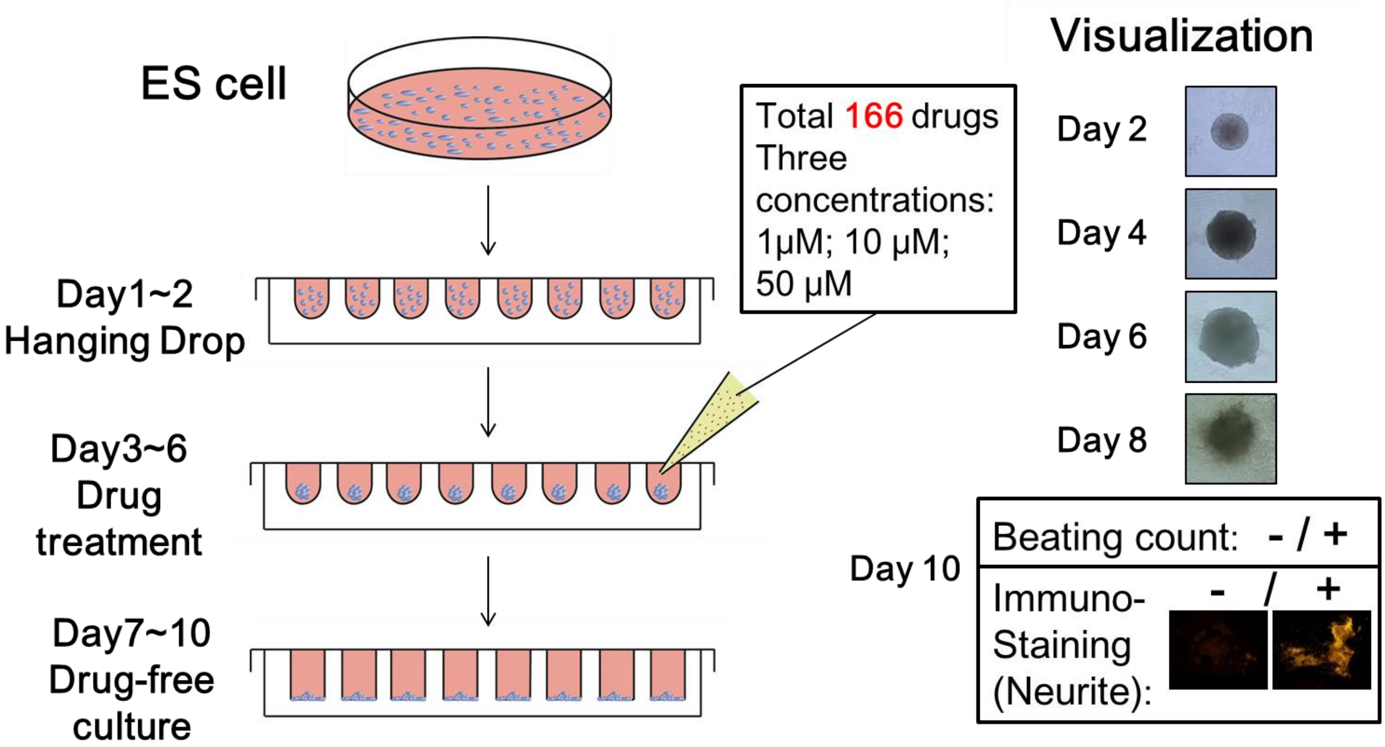

Figure S1

Screening of FDA pregnancy category D and X drugs

Mouse ES cells were plated in U-type 98 well plates and EBs were allowed to form. After 2 days in culture, EBs were treated with 3 concentrations of each FDA pregnancy Cat.D or Cat.X drug until day 6. Treated EBs were transferred to flat bottom 98 well plates and cultured until day 10 without drugs. Photographs of EBs under a light microscope were taken on days 4, 6 and 8. On day 10, EBs were visually evaluated for cardiomyocyte beating and immunostained to detect neurites.

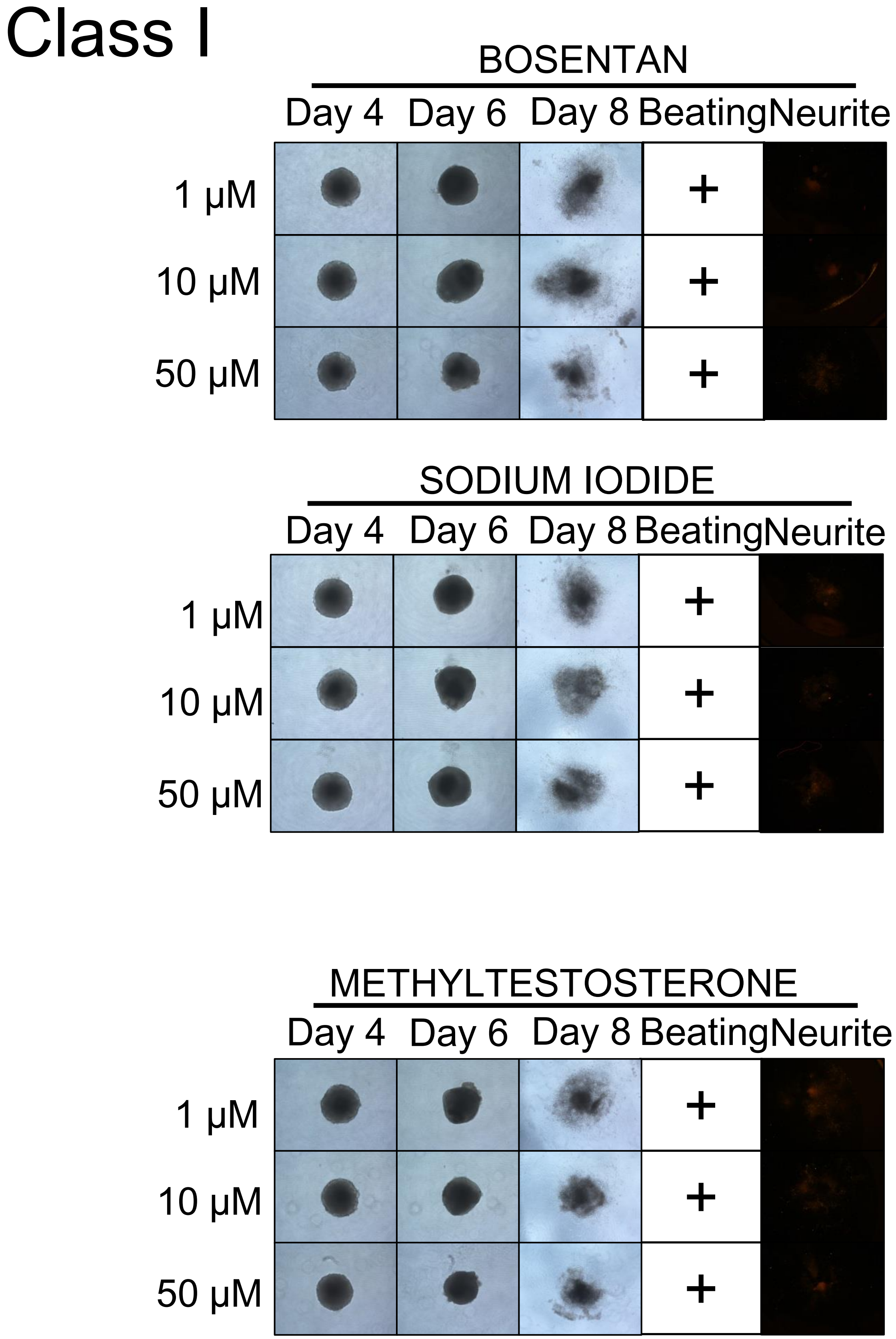

Yu.et al Fig. S1

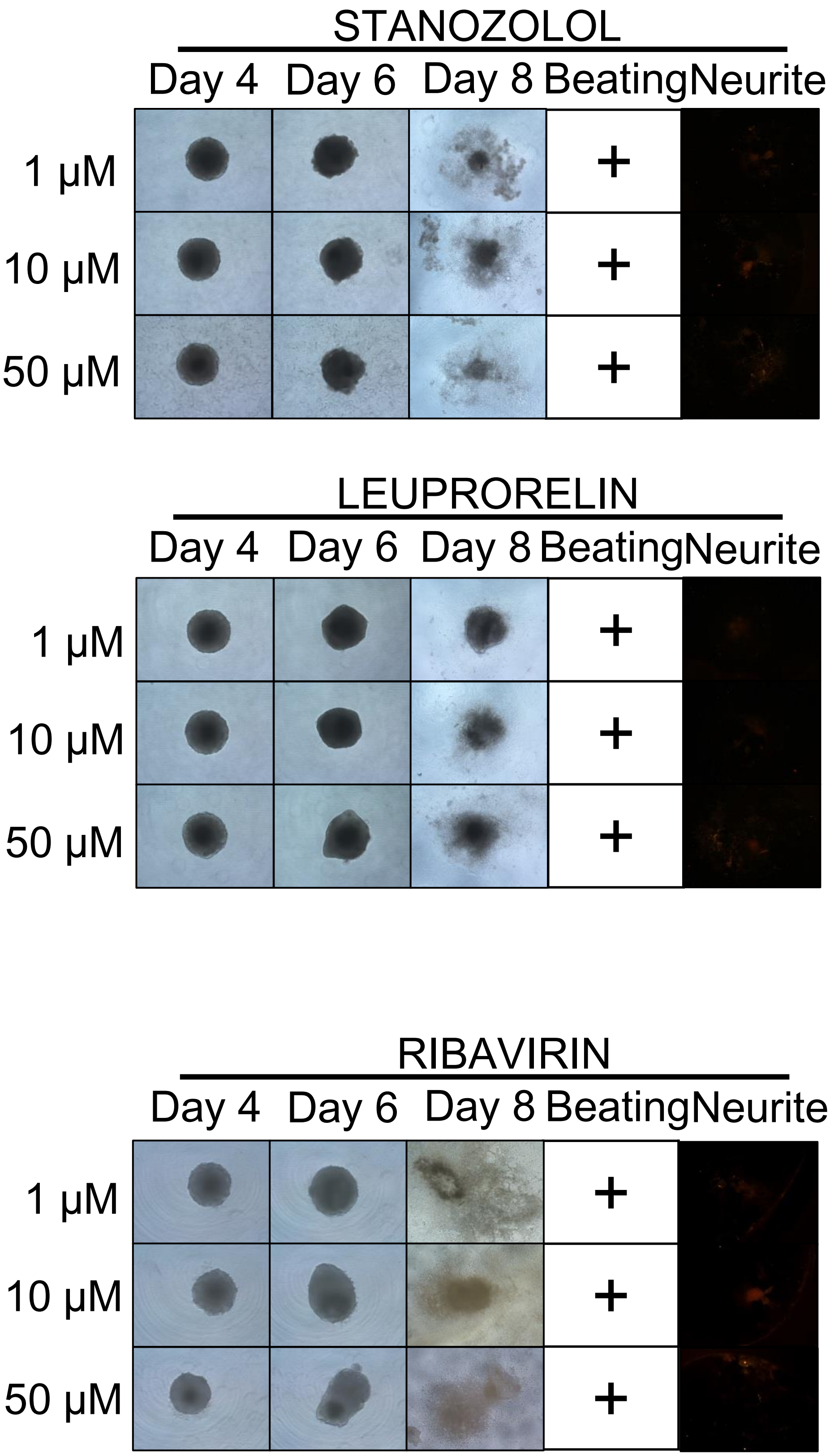

Yu.et al Fig. S1

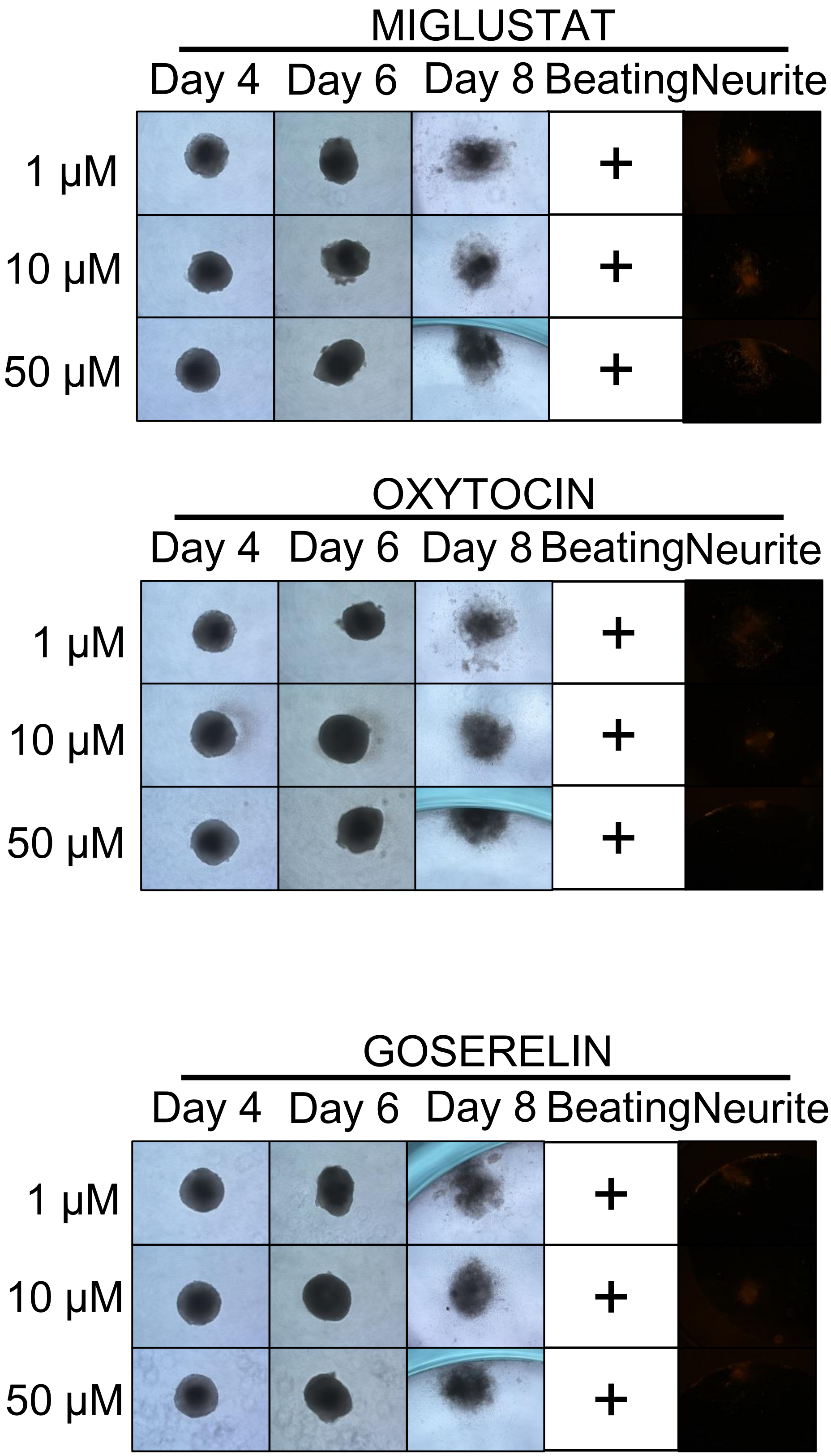

Yu.et al Fig. S1

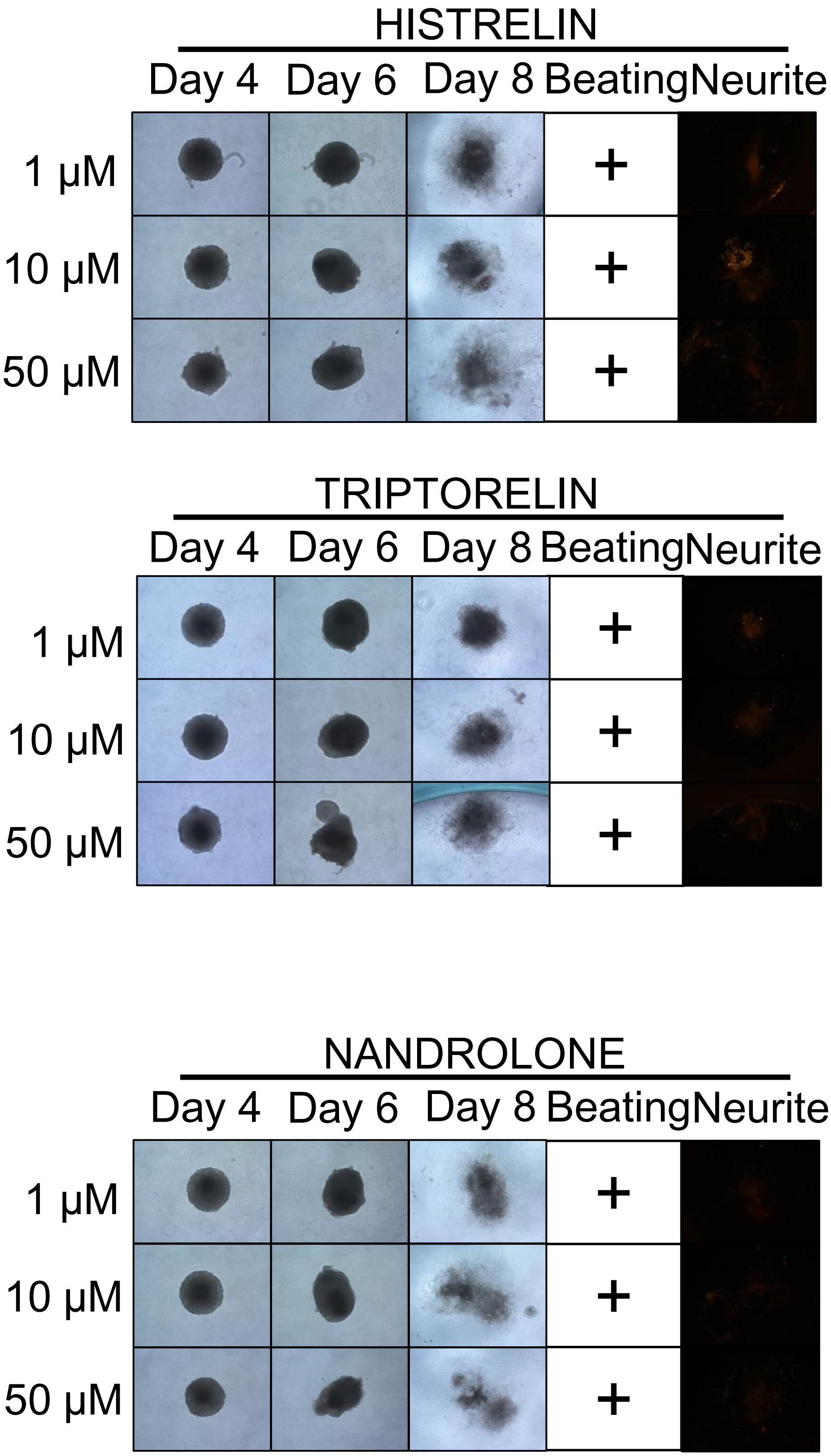

Yu.et al Fig. S1

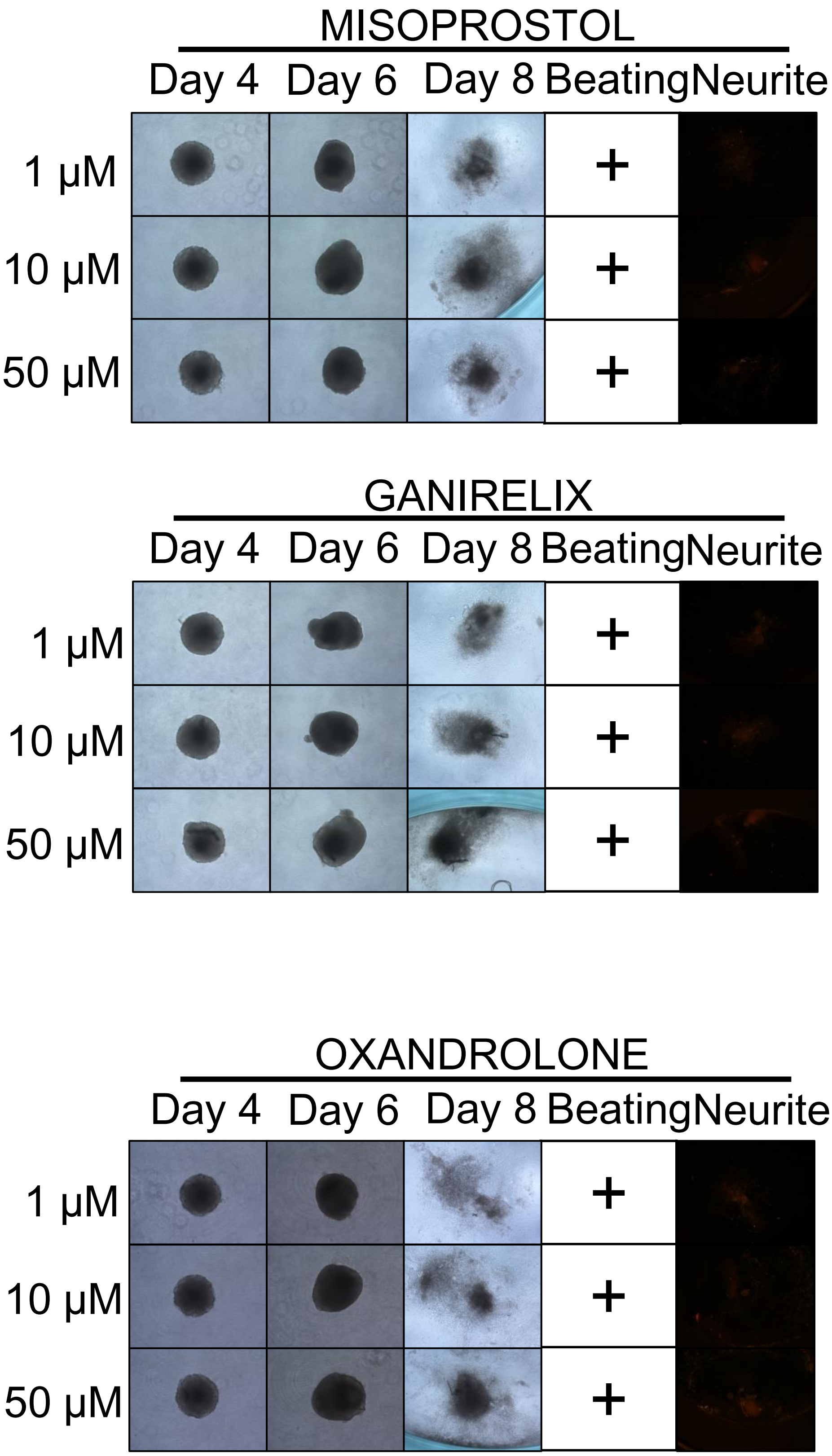

Yu.et al Fig. S1

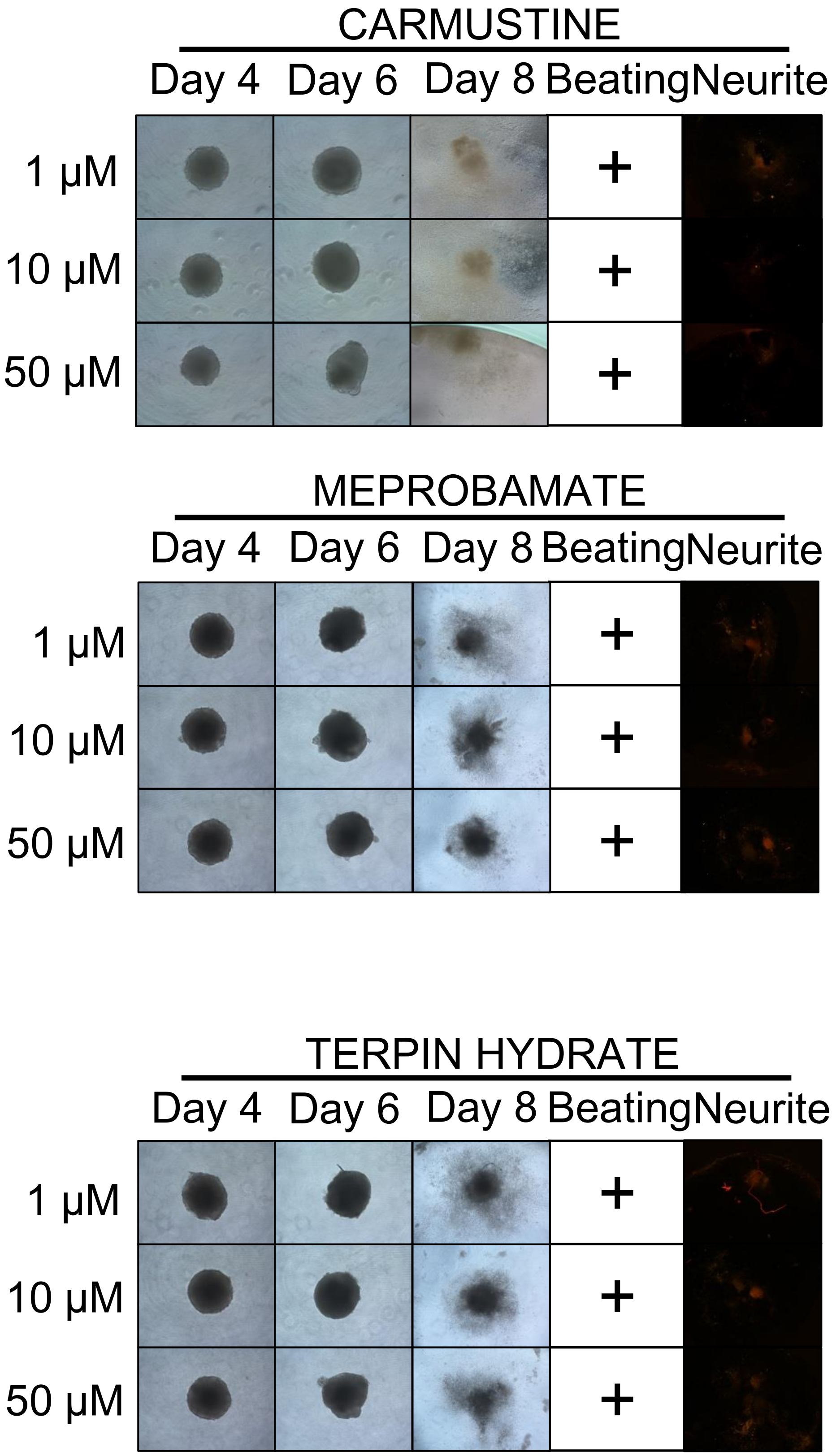

Yu.et al Fig. S1

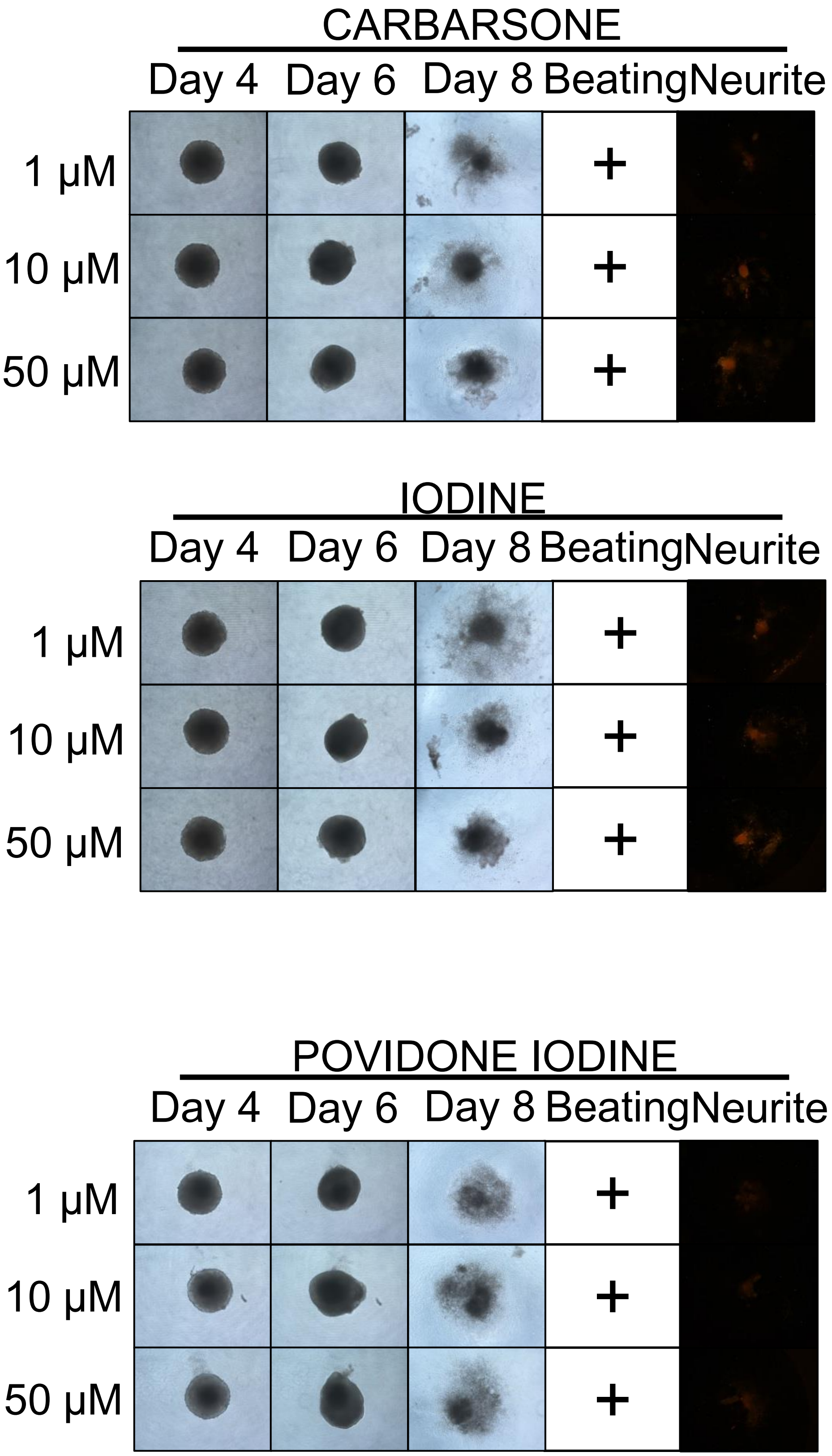

Yu.et al Fig. S1

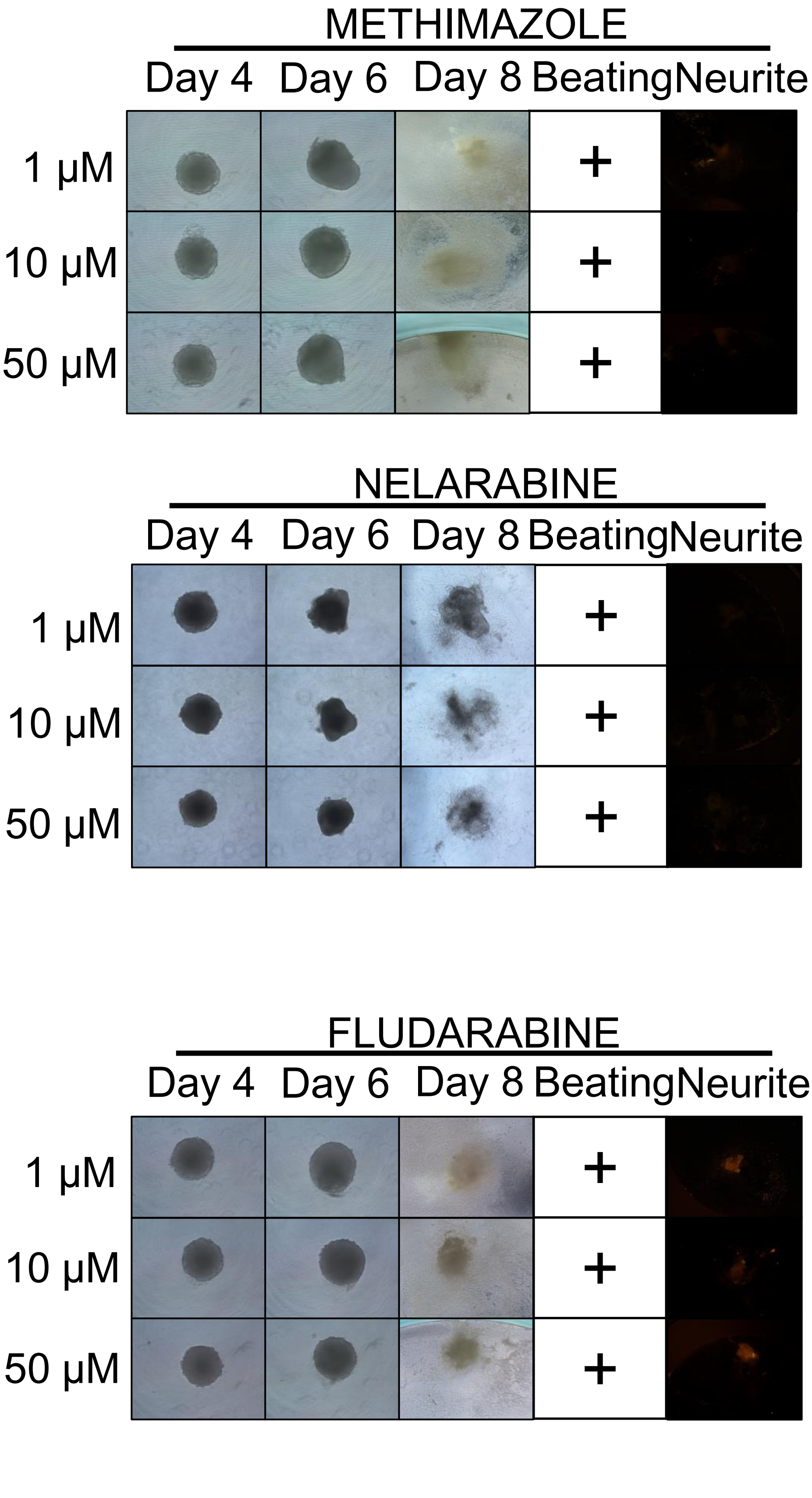

Yu.et al Fig. S1

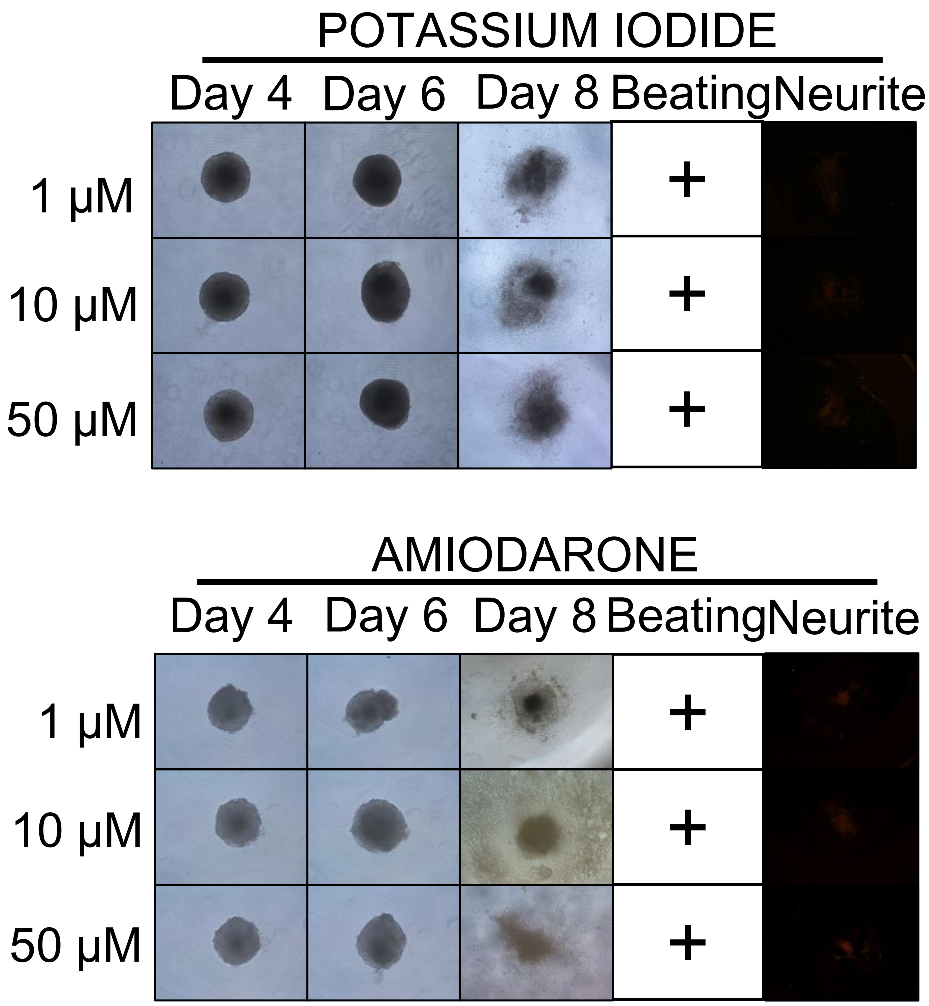

Yu.et al Fig. S1

Class II

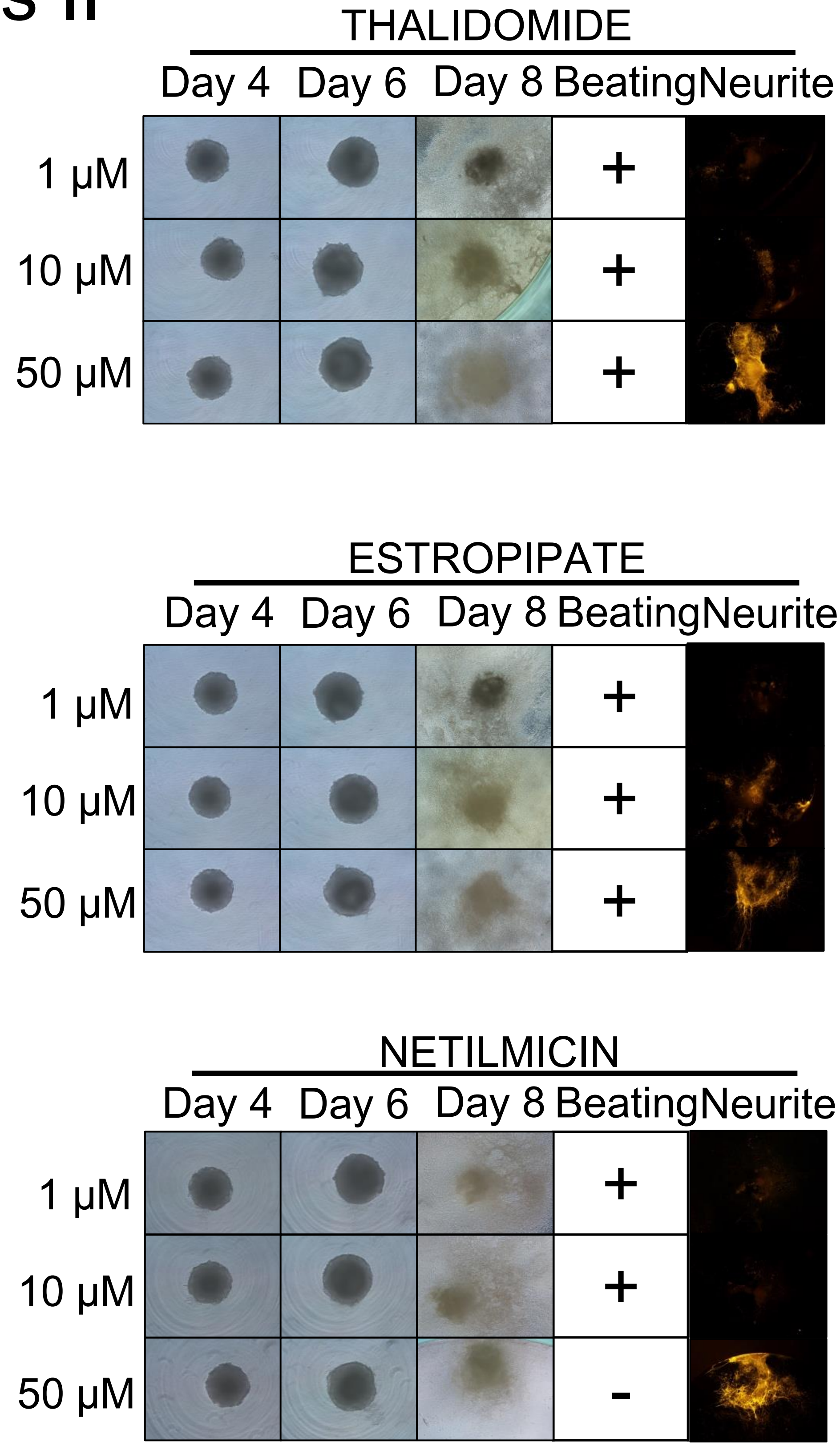

Yu.et al Fig. S1

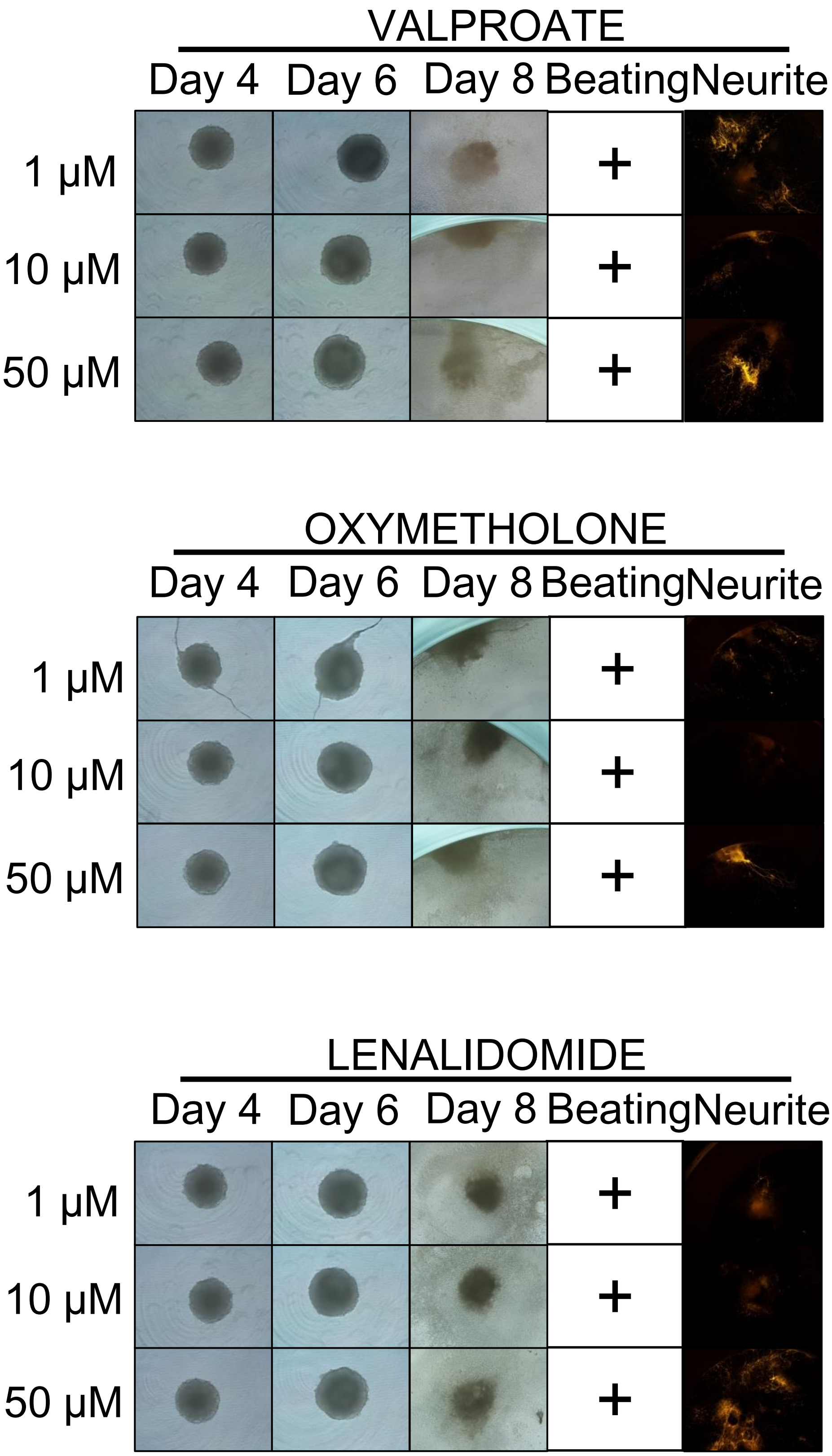

Yu.et al Fig. S1

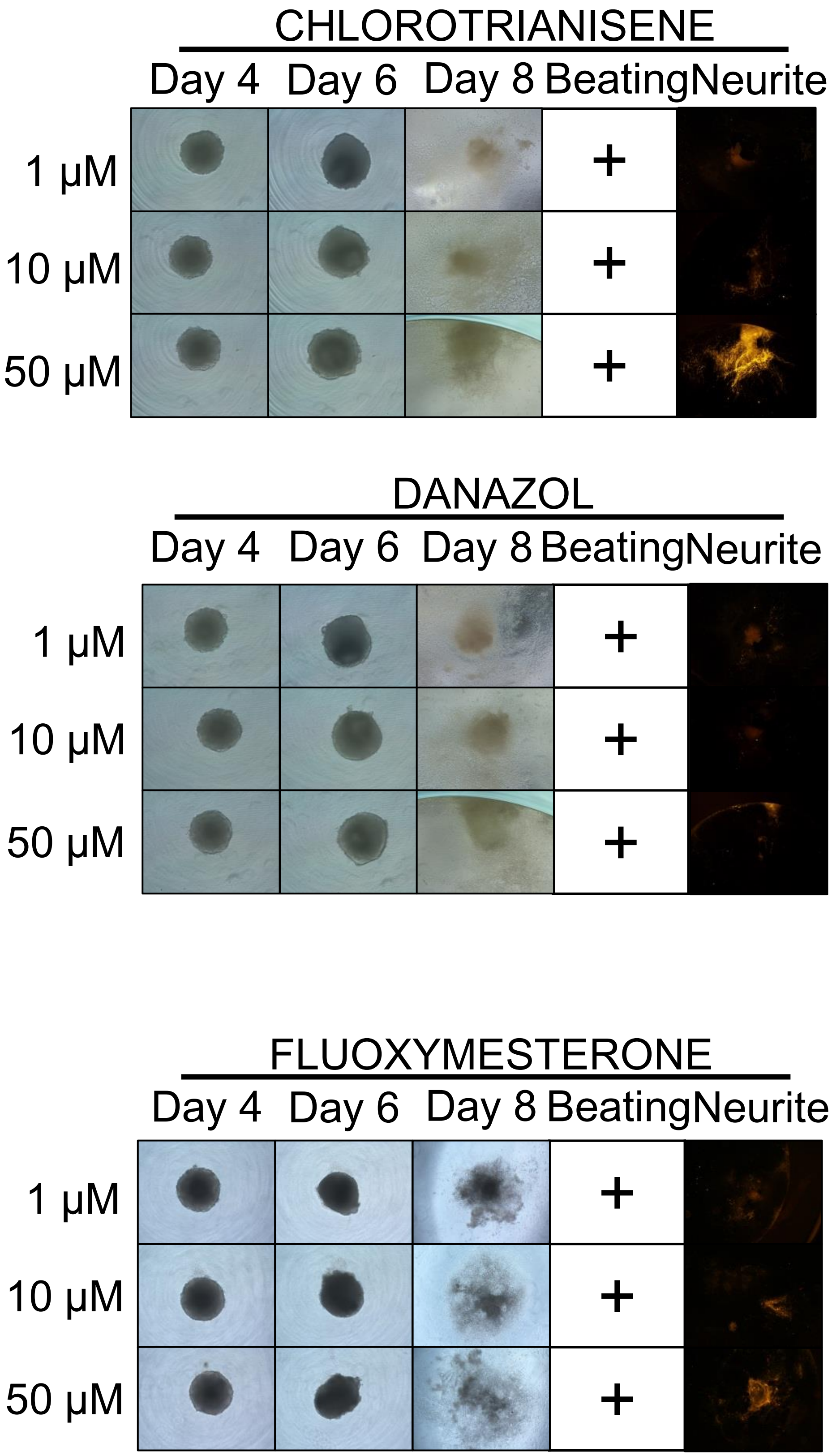

Yu.et al Fig. S1

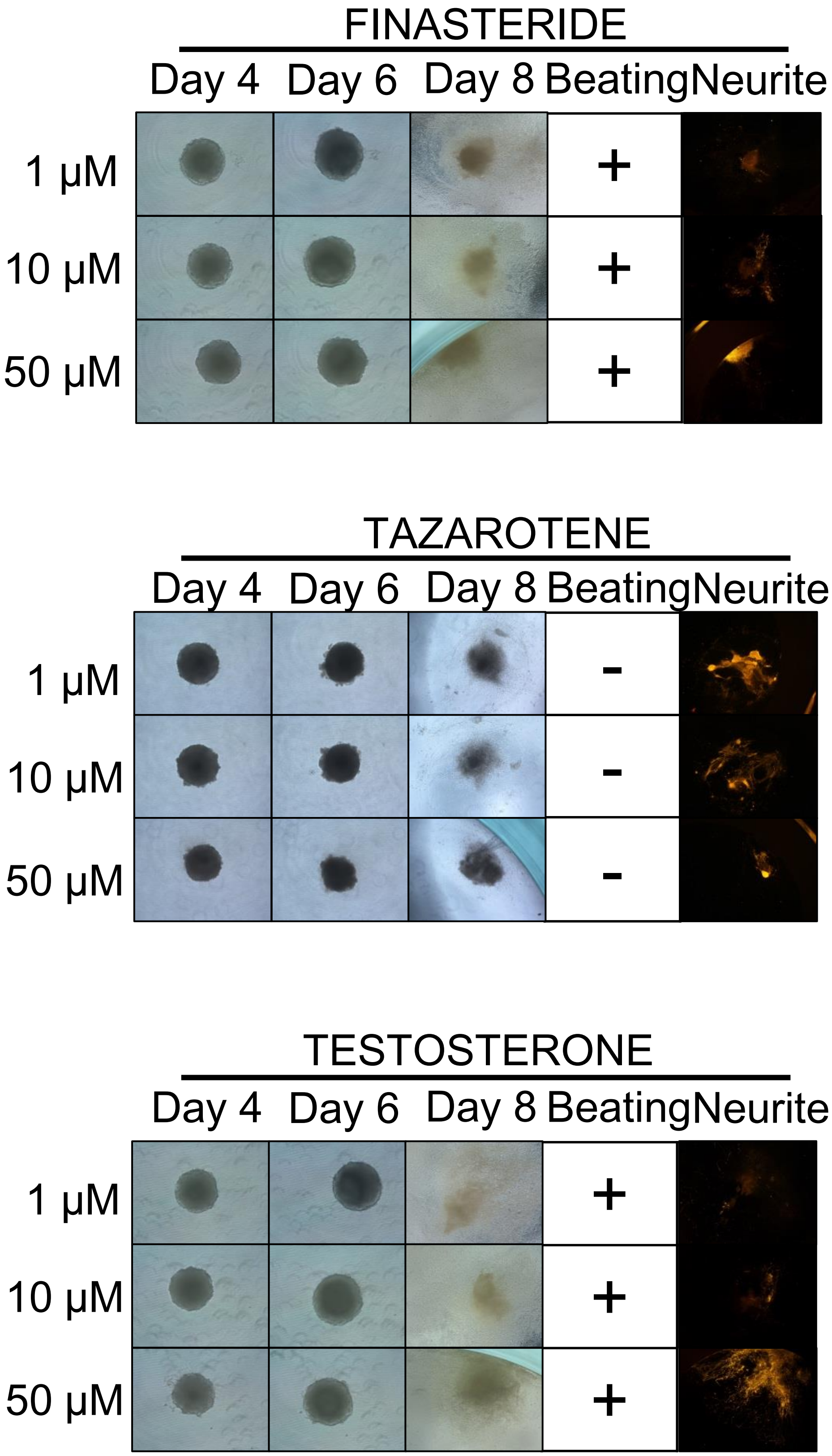

Yu.et al Fig. S1

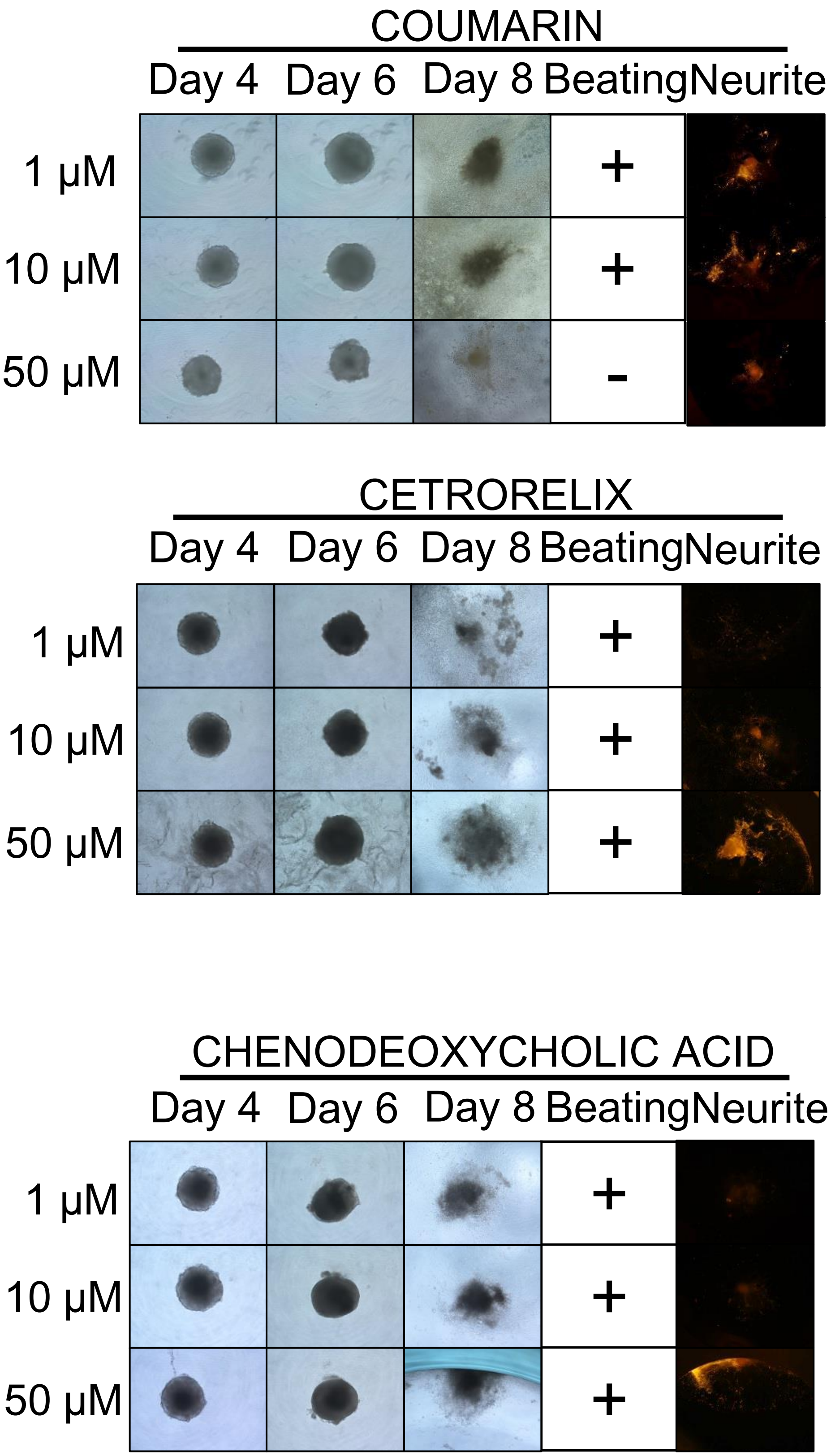

Yu.et al Fig. S1

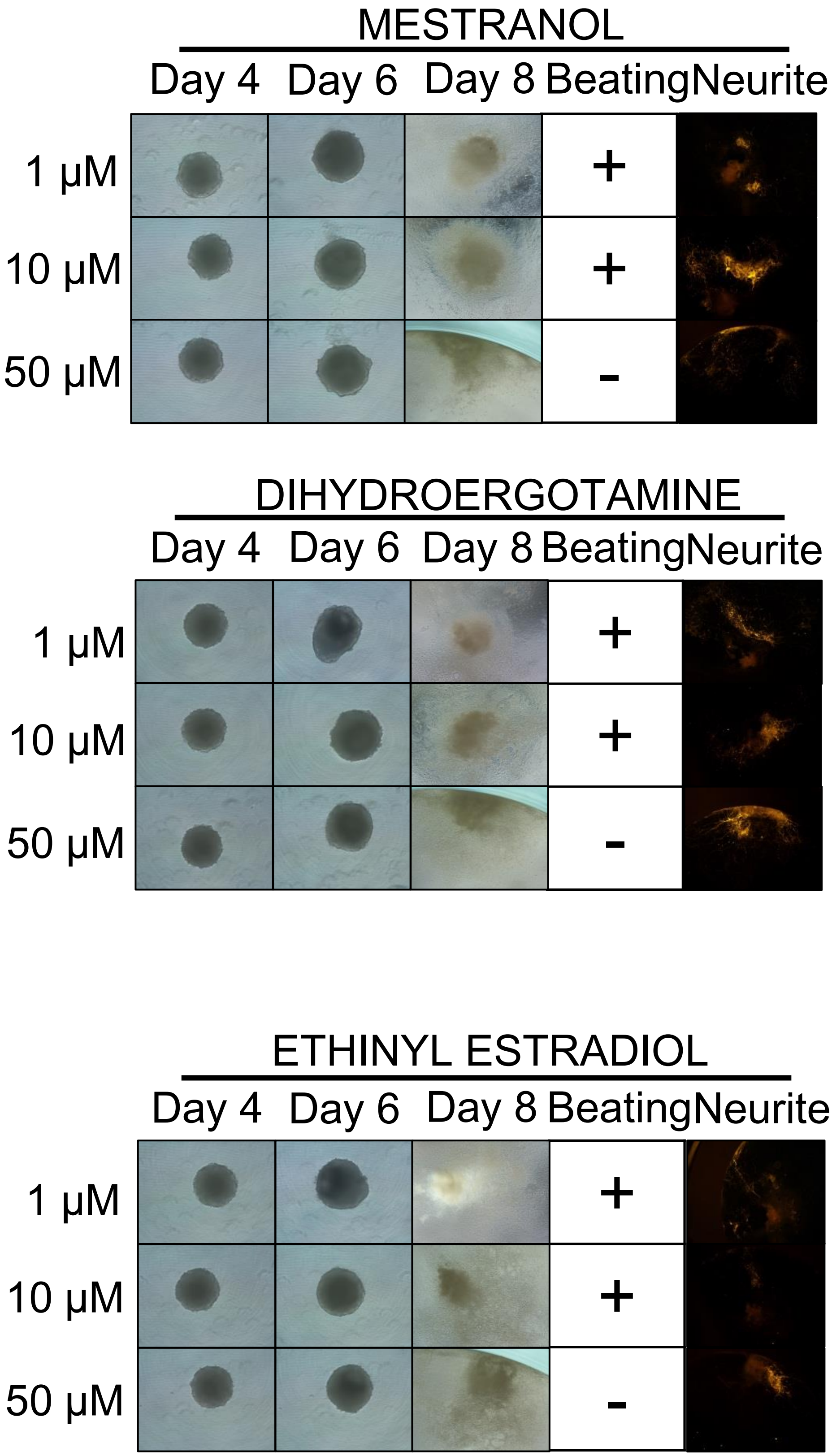

Yu.et al Fig. S1

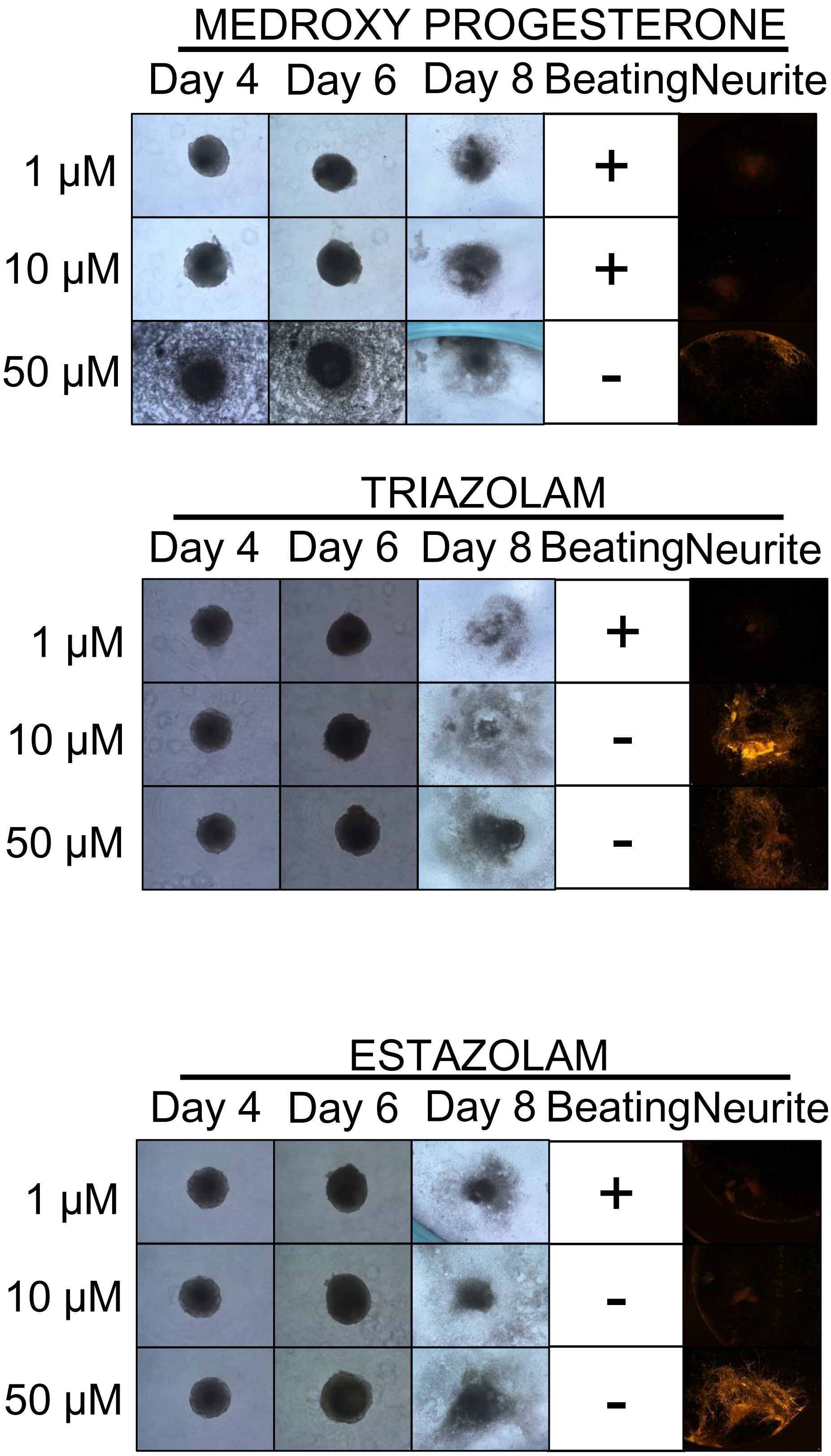

Yu.et al Fig. S1

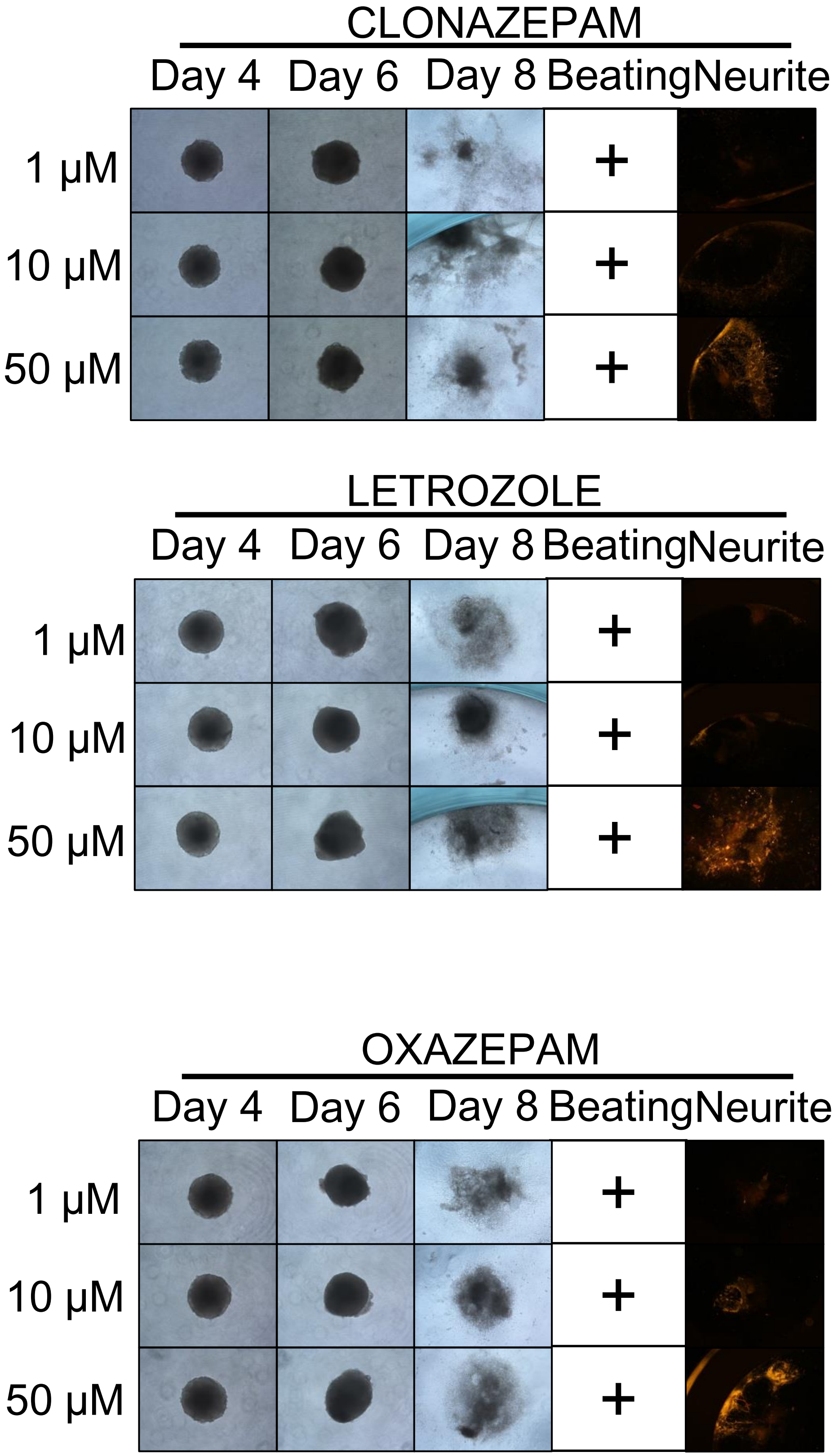

Yu.et al Fig. S1

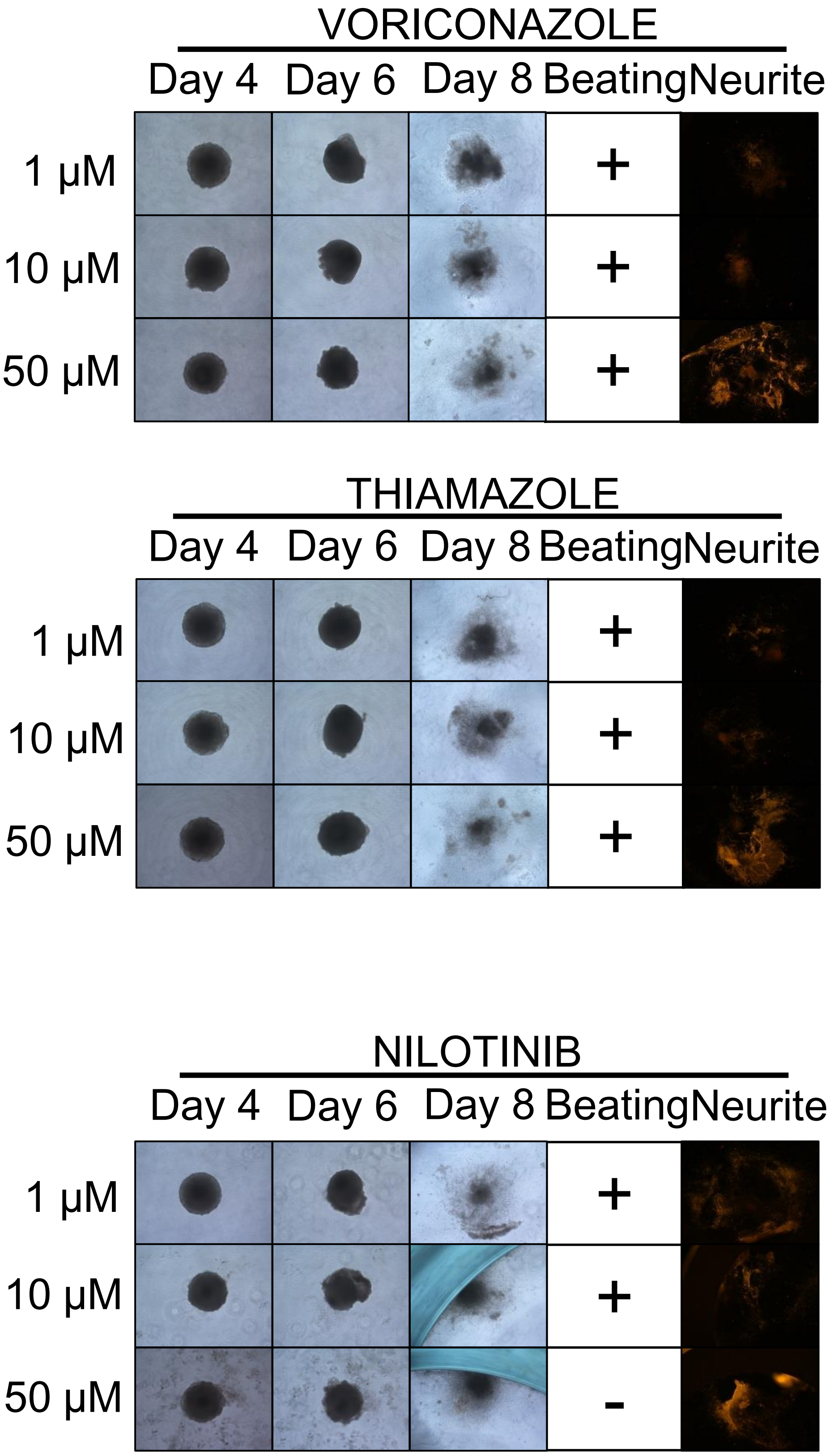

Yu.et al Fig. S1

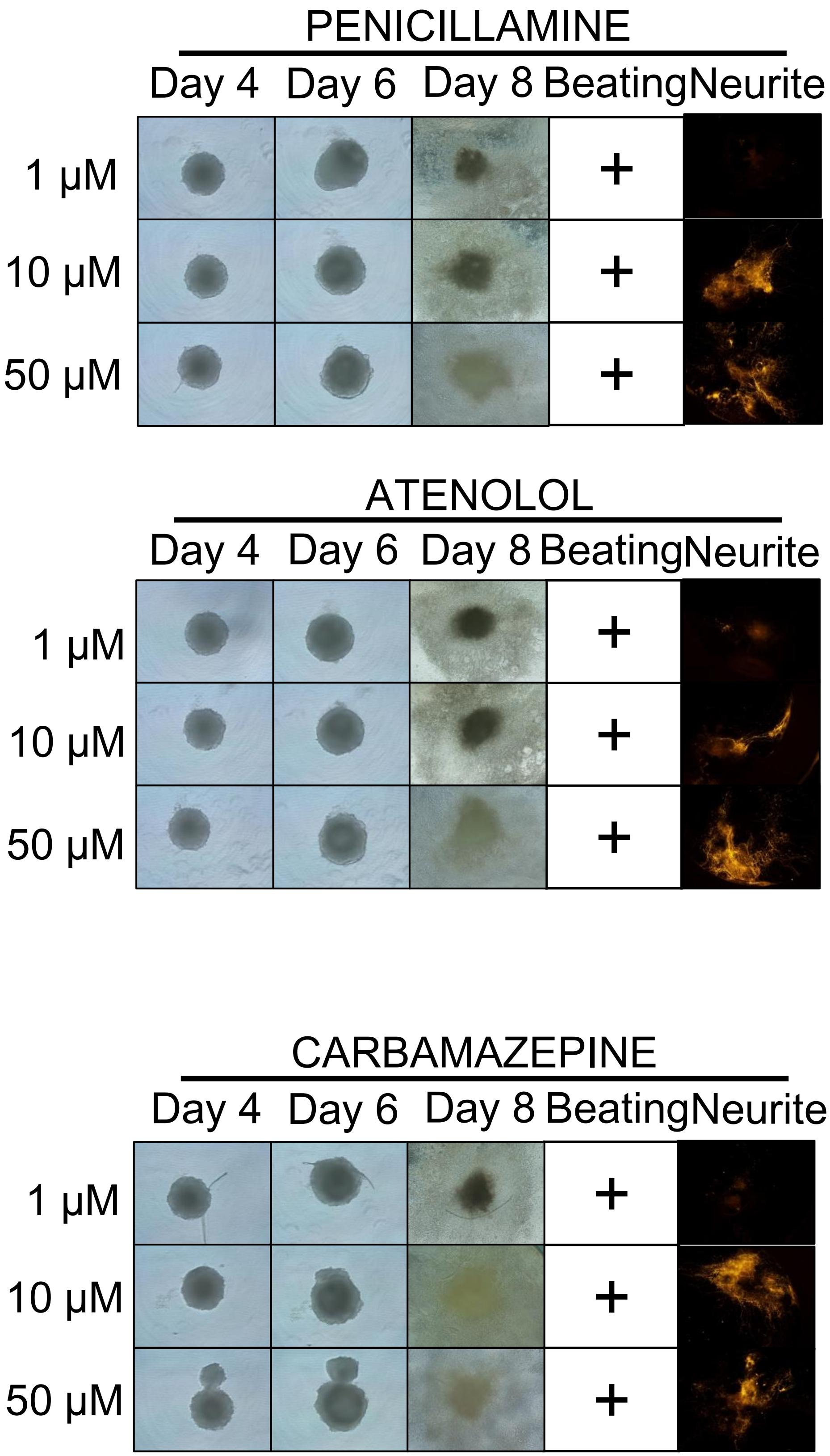

Yu.et al Fig. S1

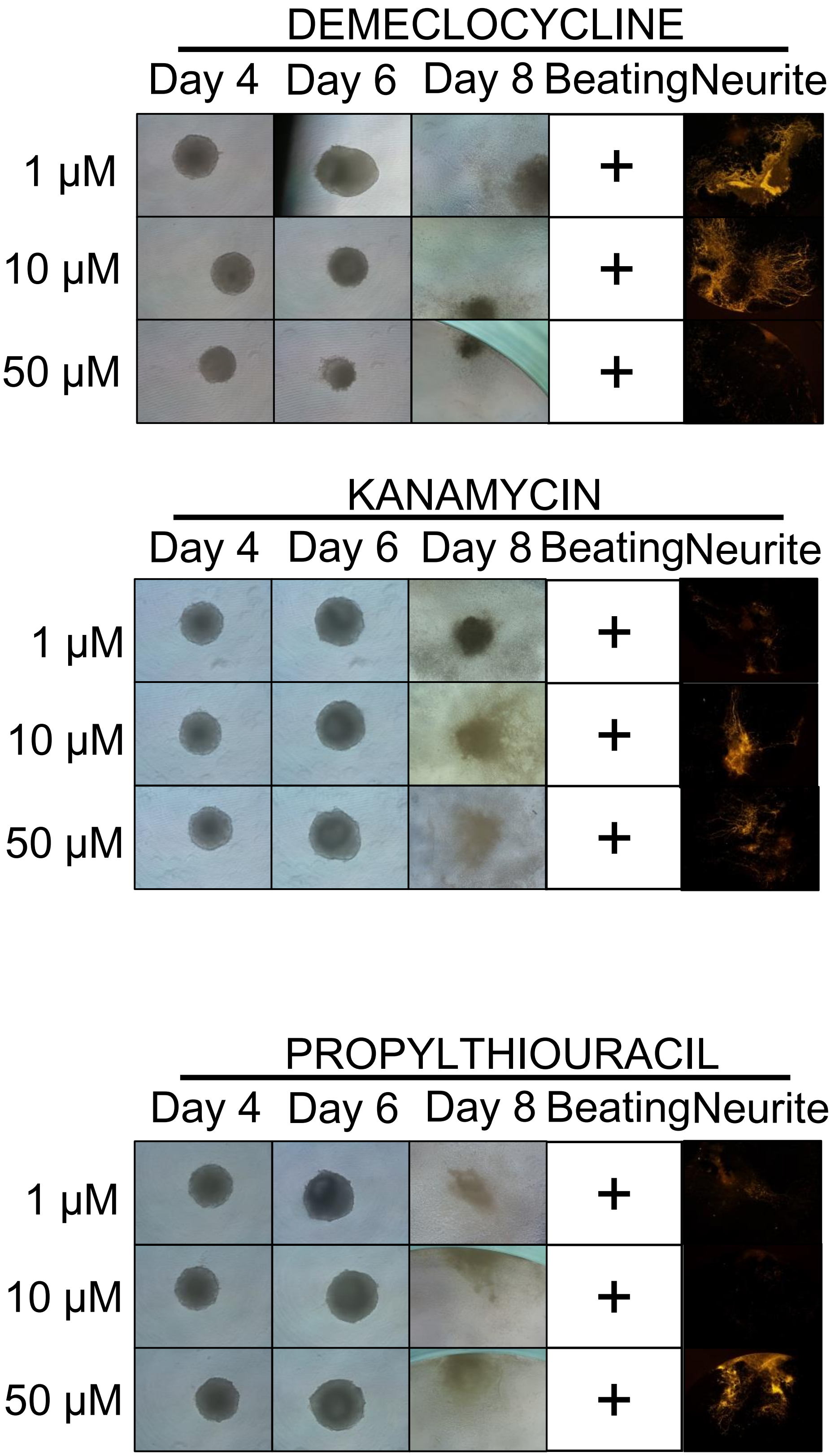

Yu.et al Fig. S1

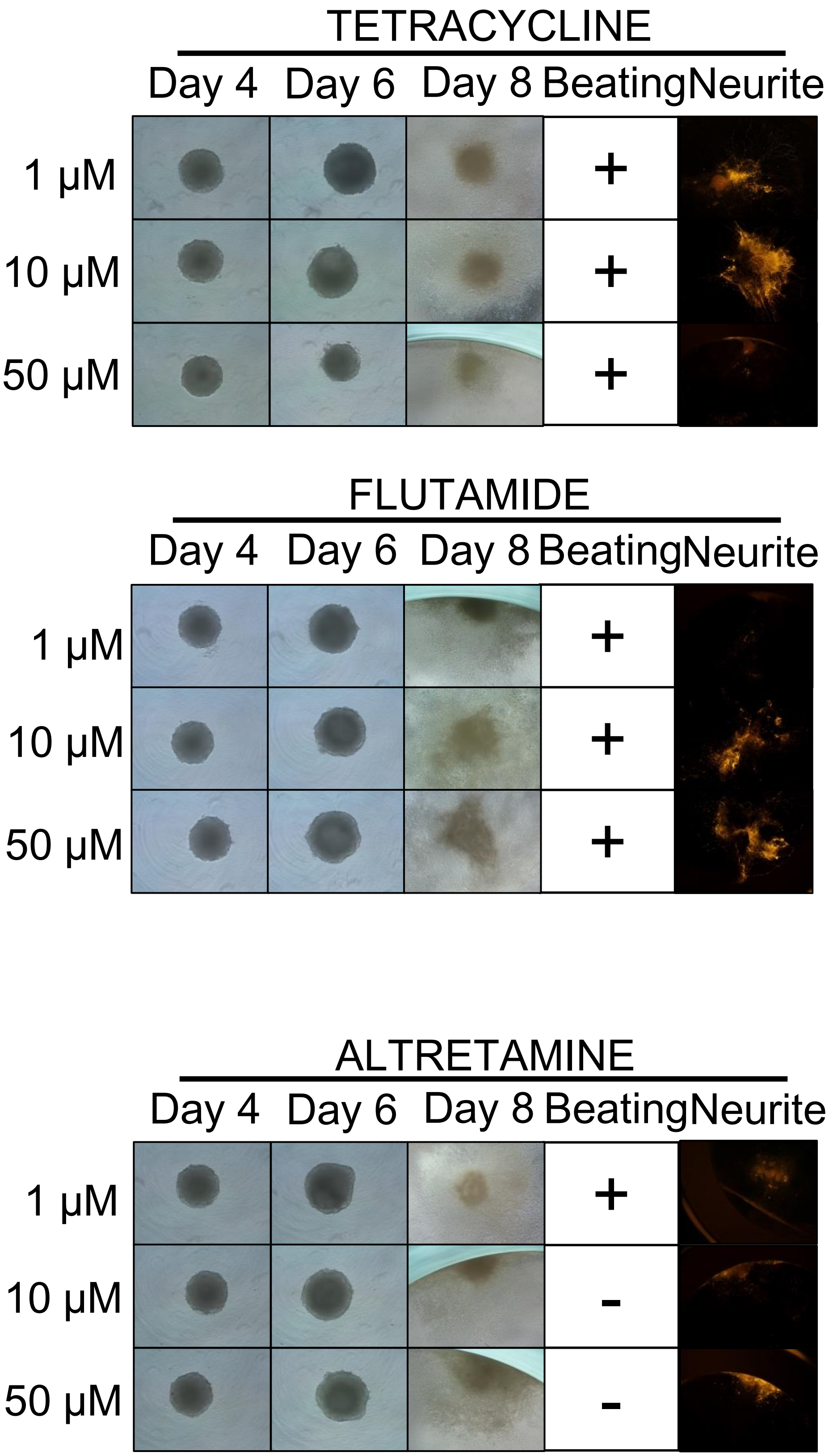

Yu.et al Fig. S1

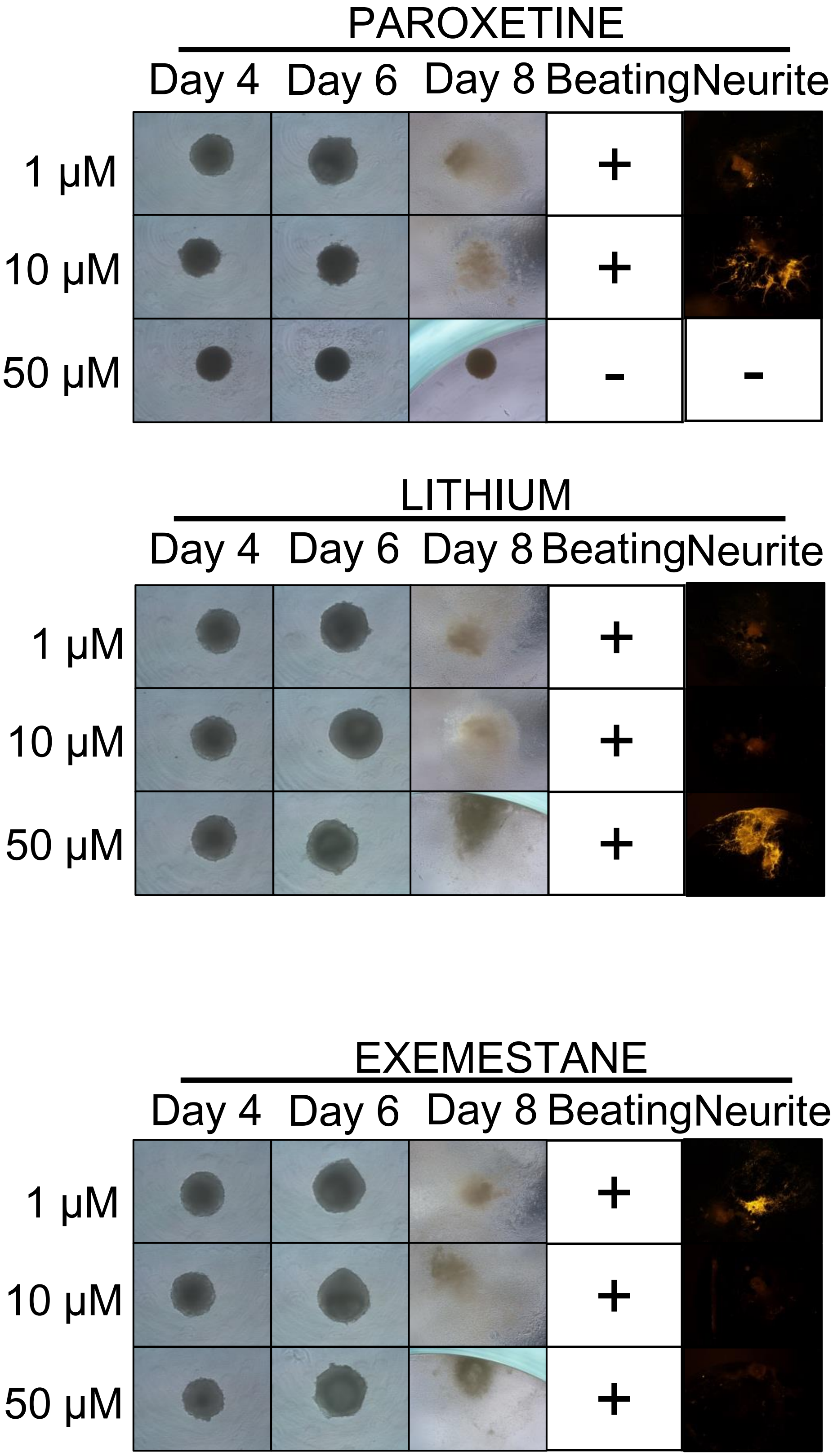

Yu.et al Fig. S1

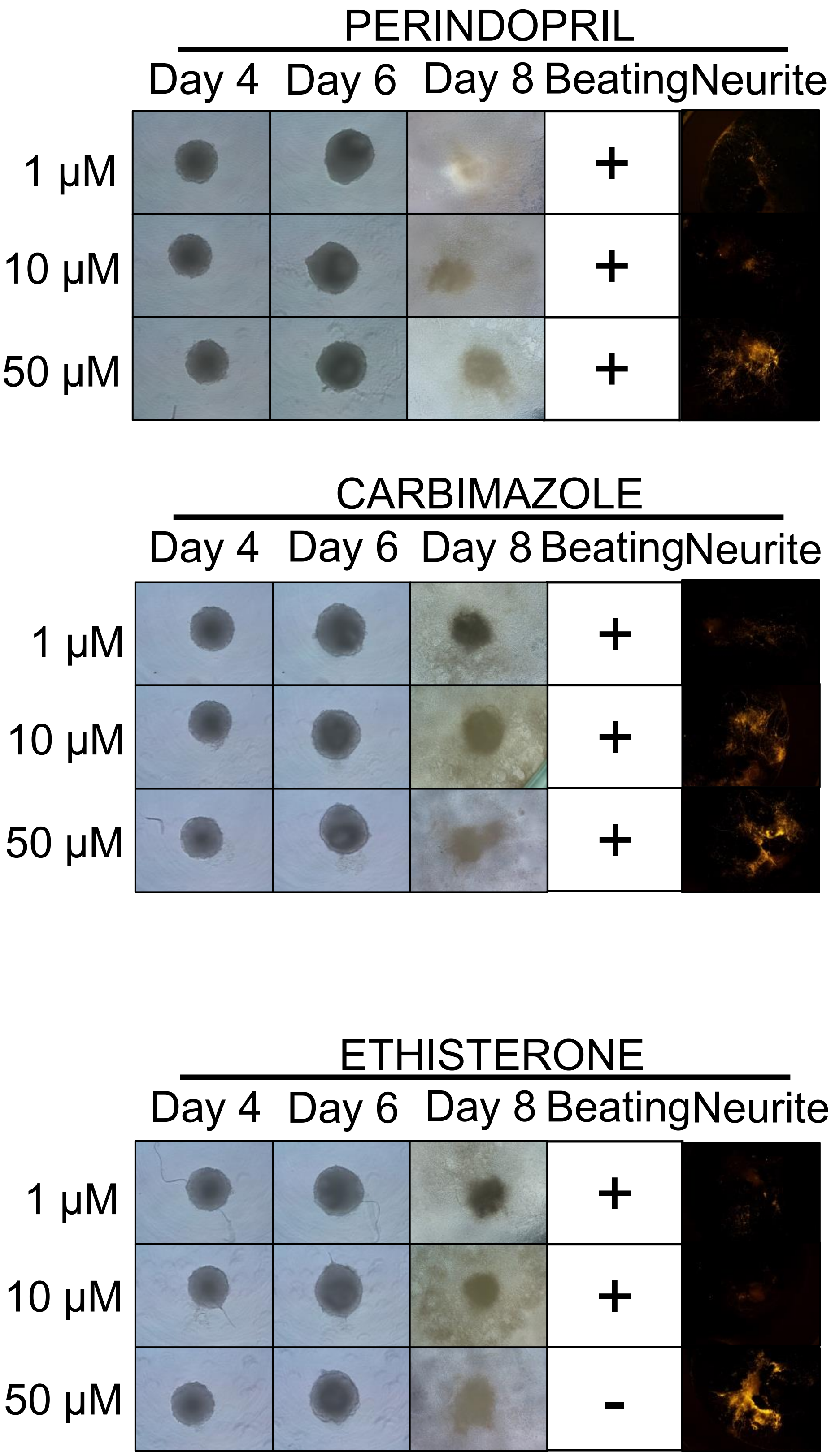

Yu.et al Fig. S1

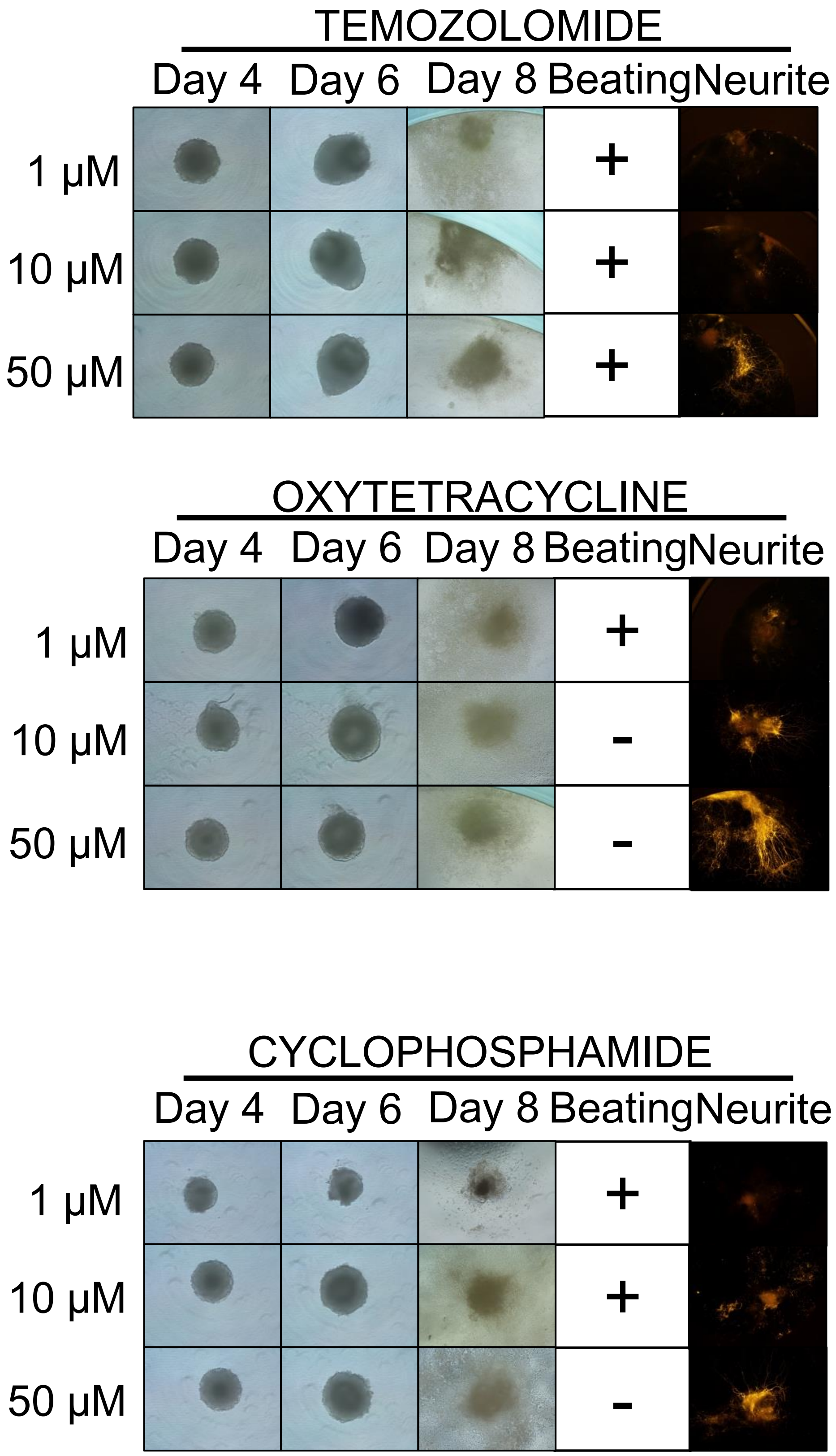

Yu.et al Fig. S1

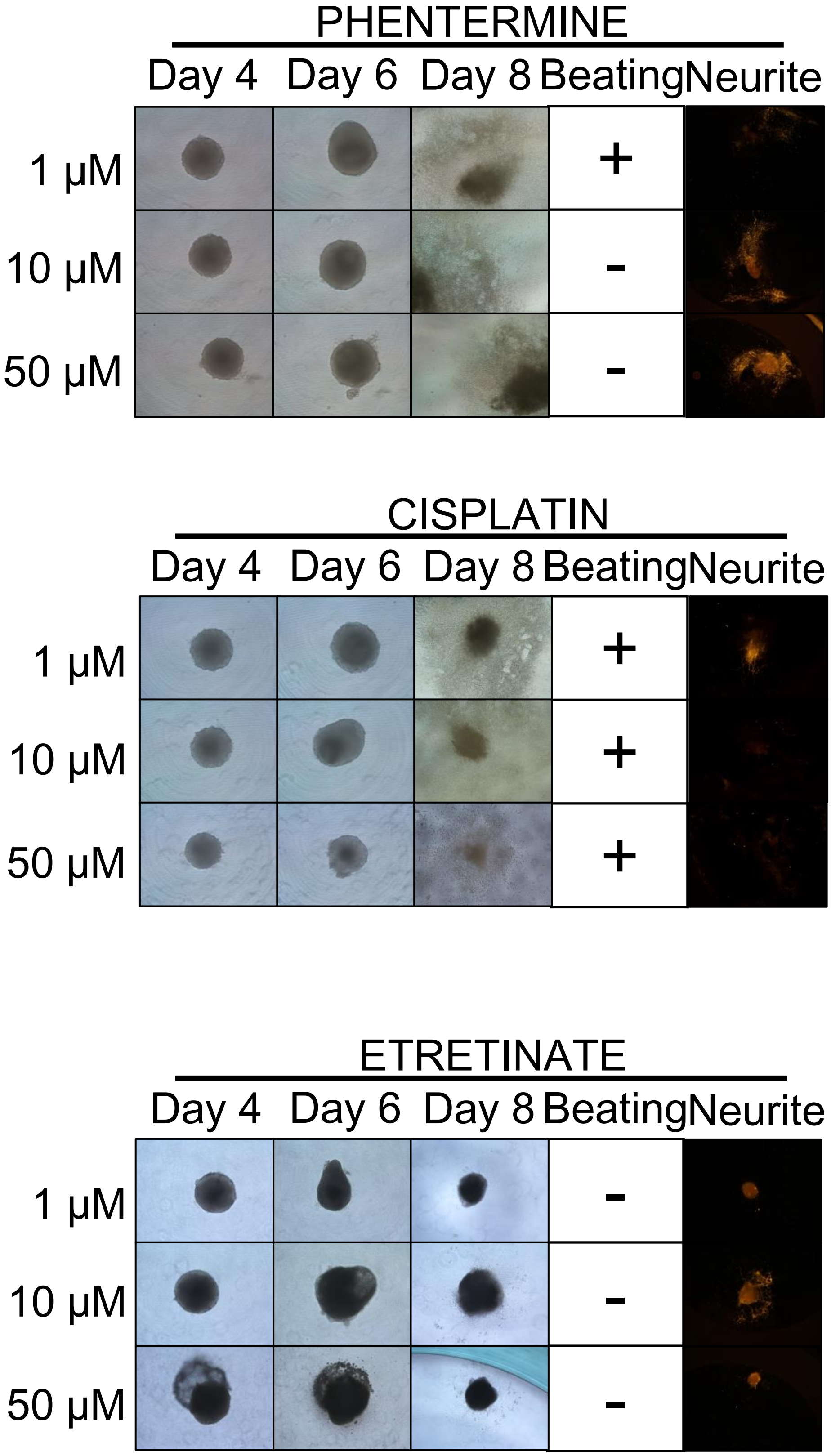

Yu.et al Fig. S1

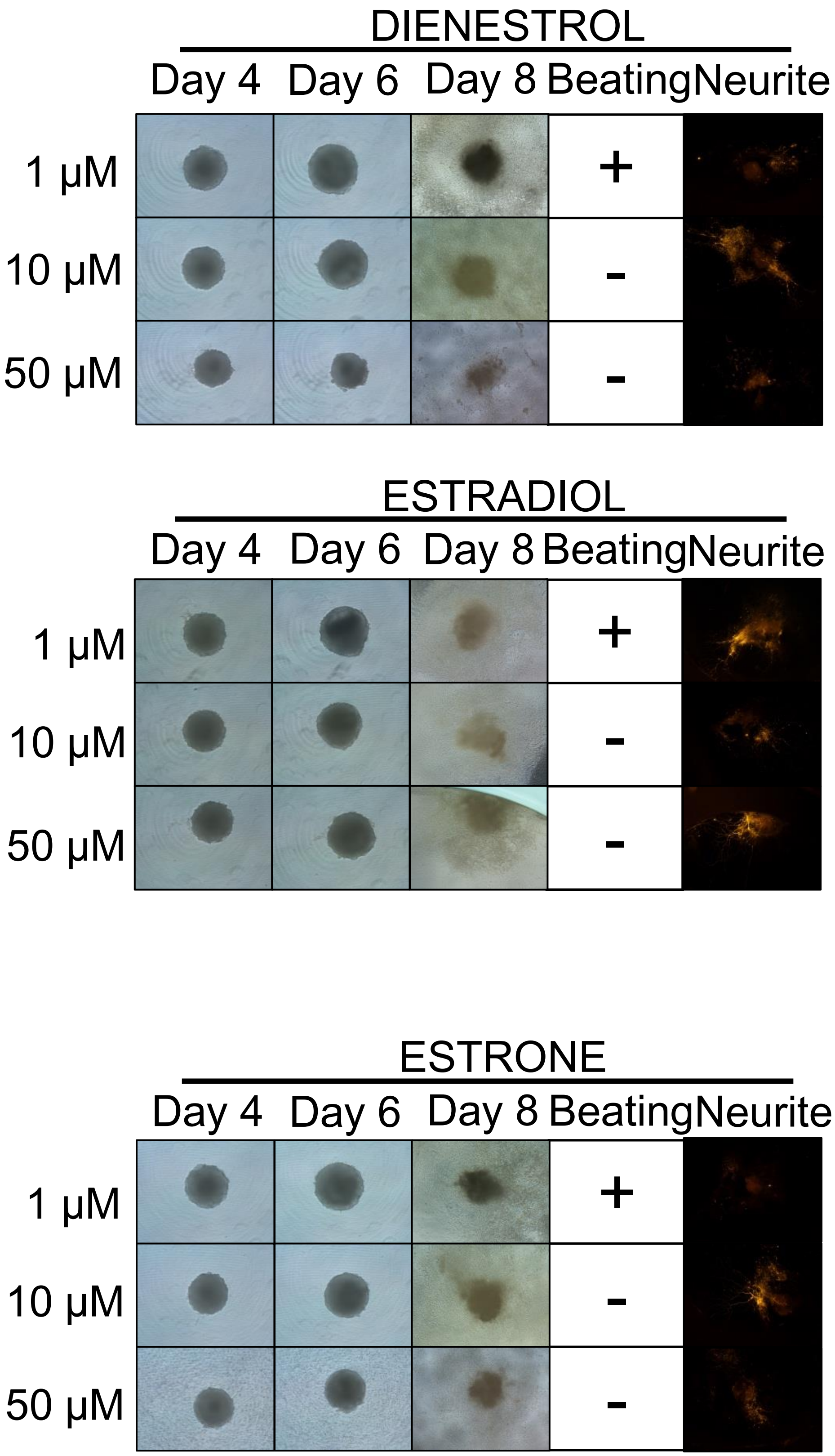

Yu.et al Fig. S1

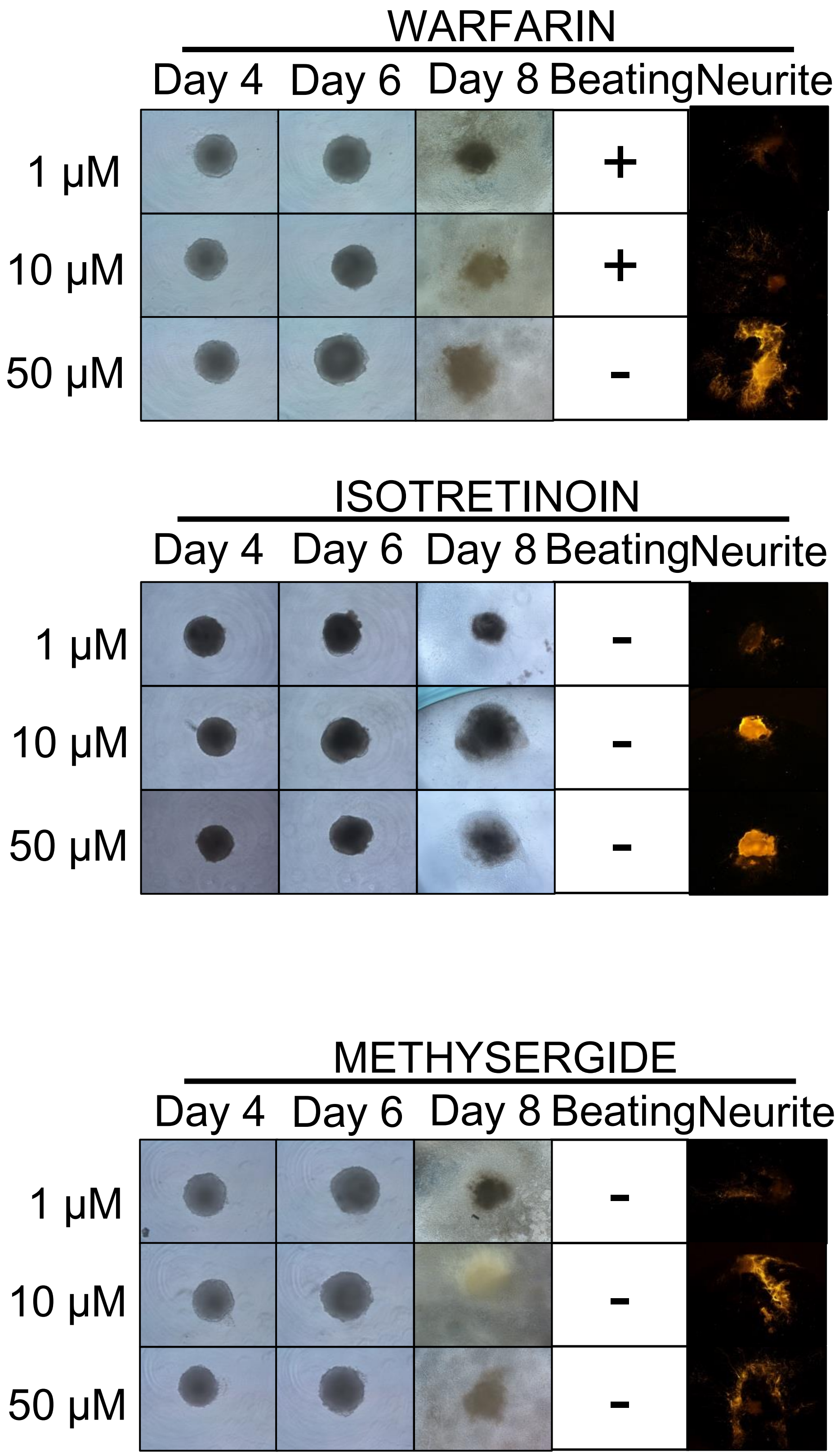

Yu.et al Fig. S1

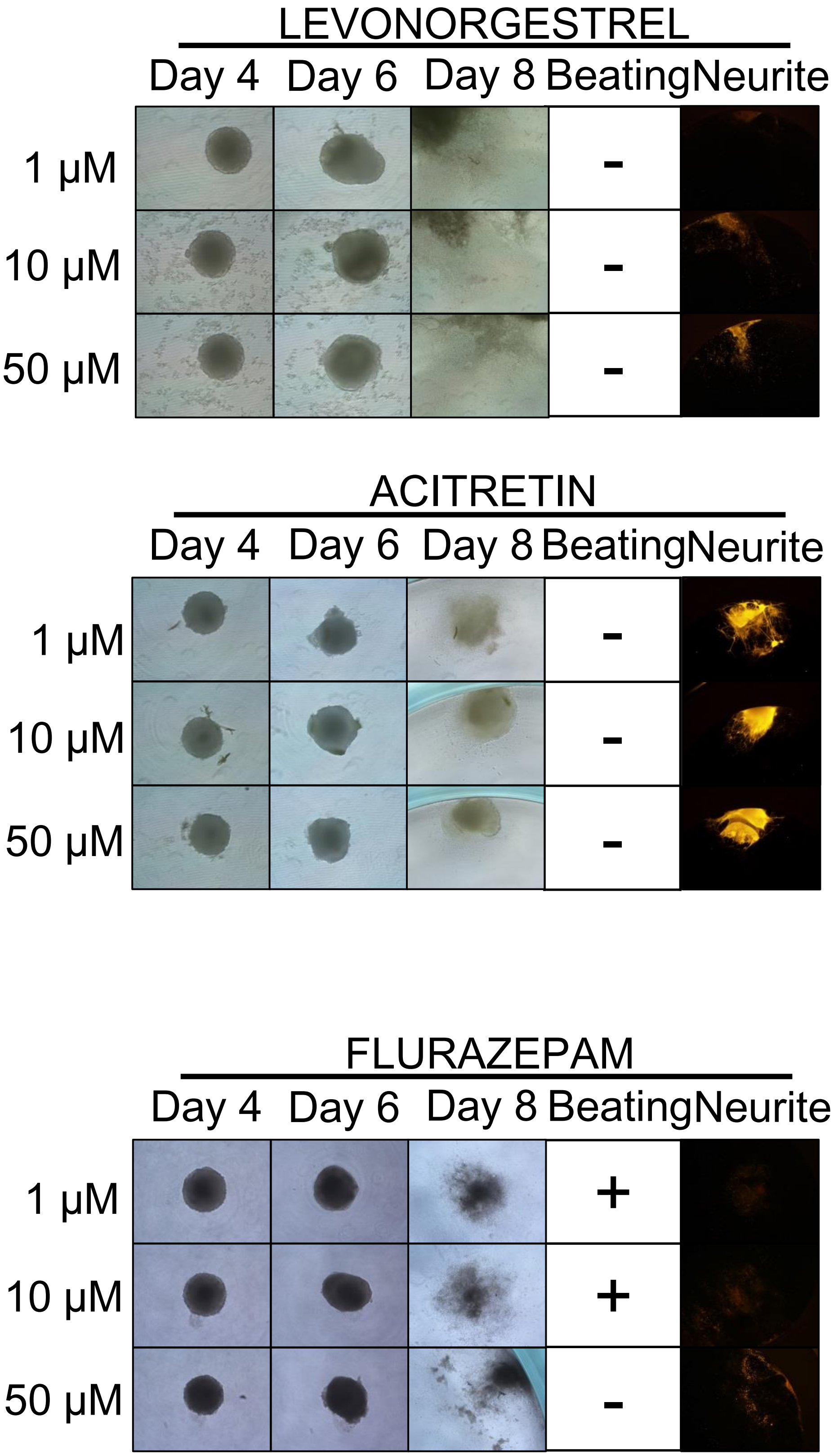

Yu.et al Fig. S1

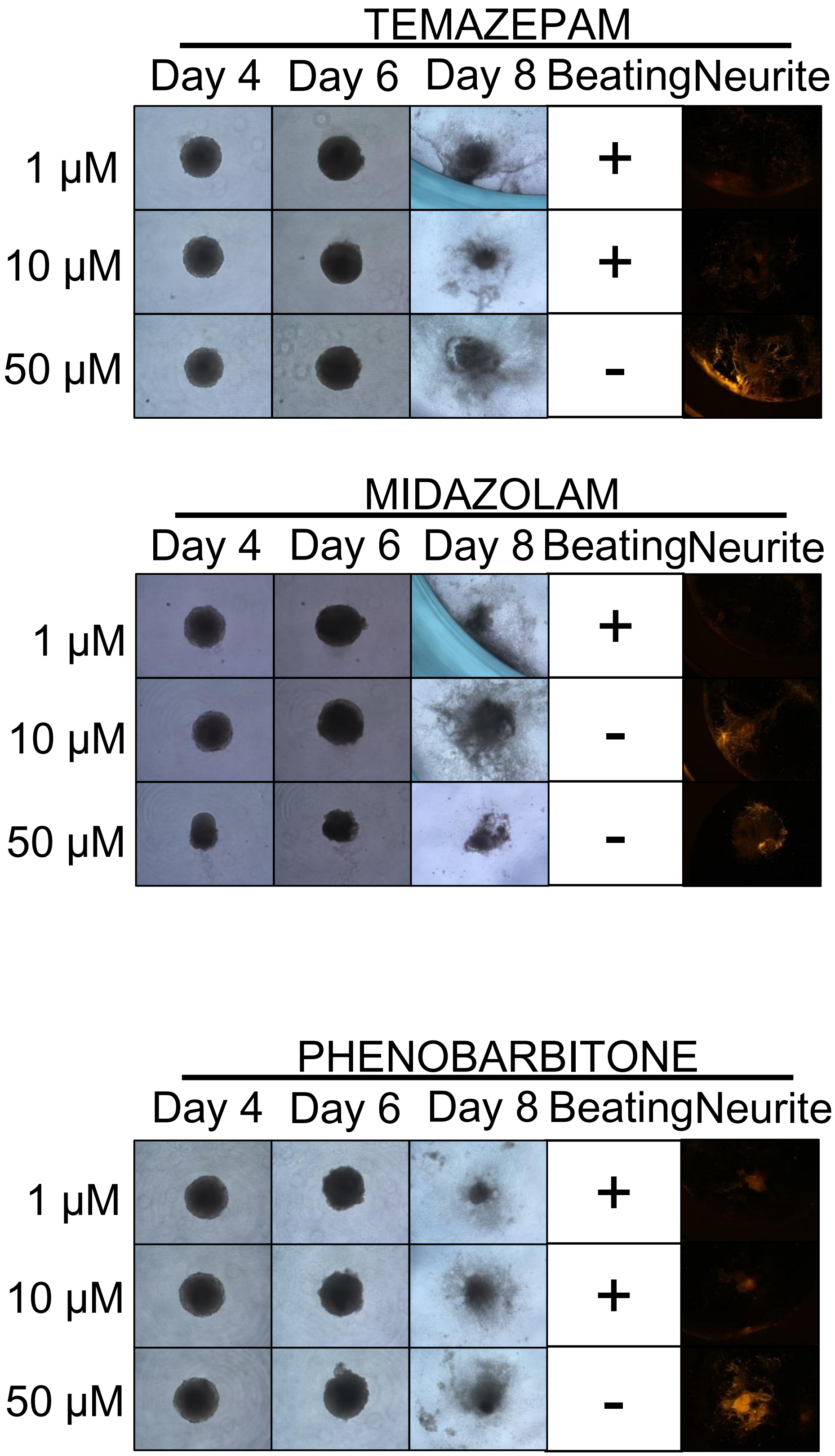

Yu.et al Fig. S1

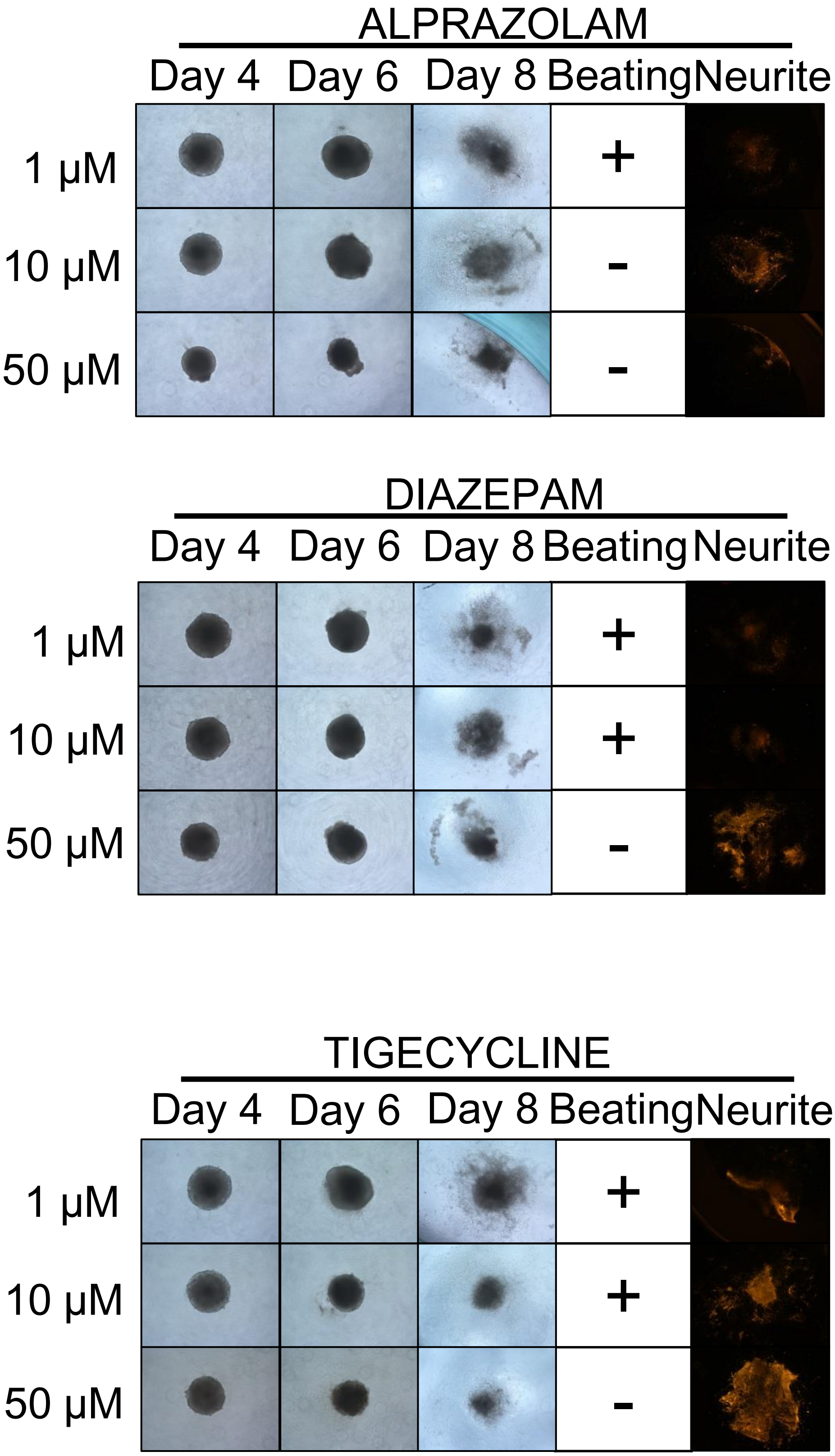

Yu.et al Fig. S1

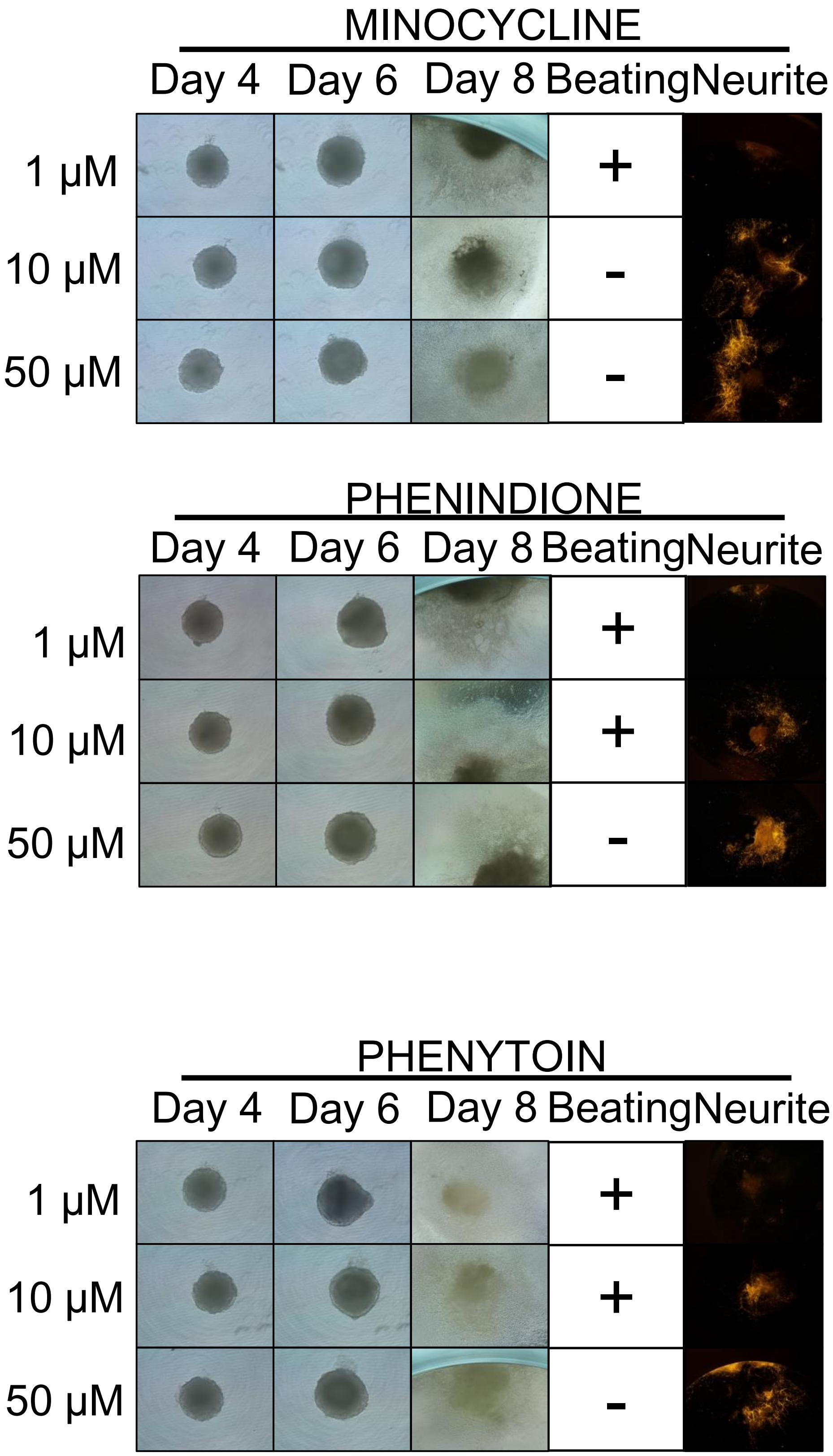

Yu.et al Fig. S1

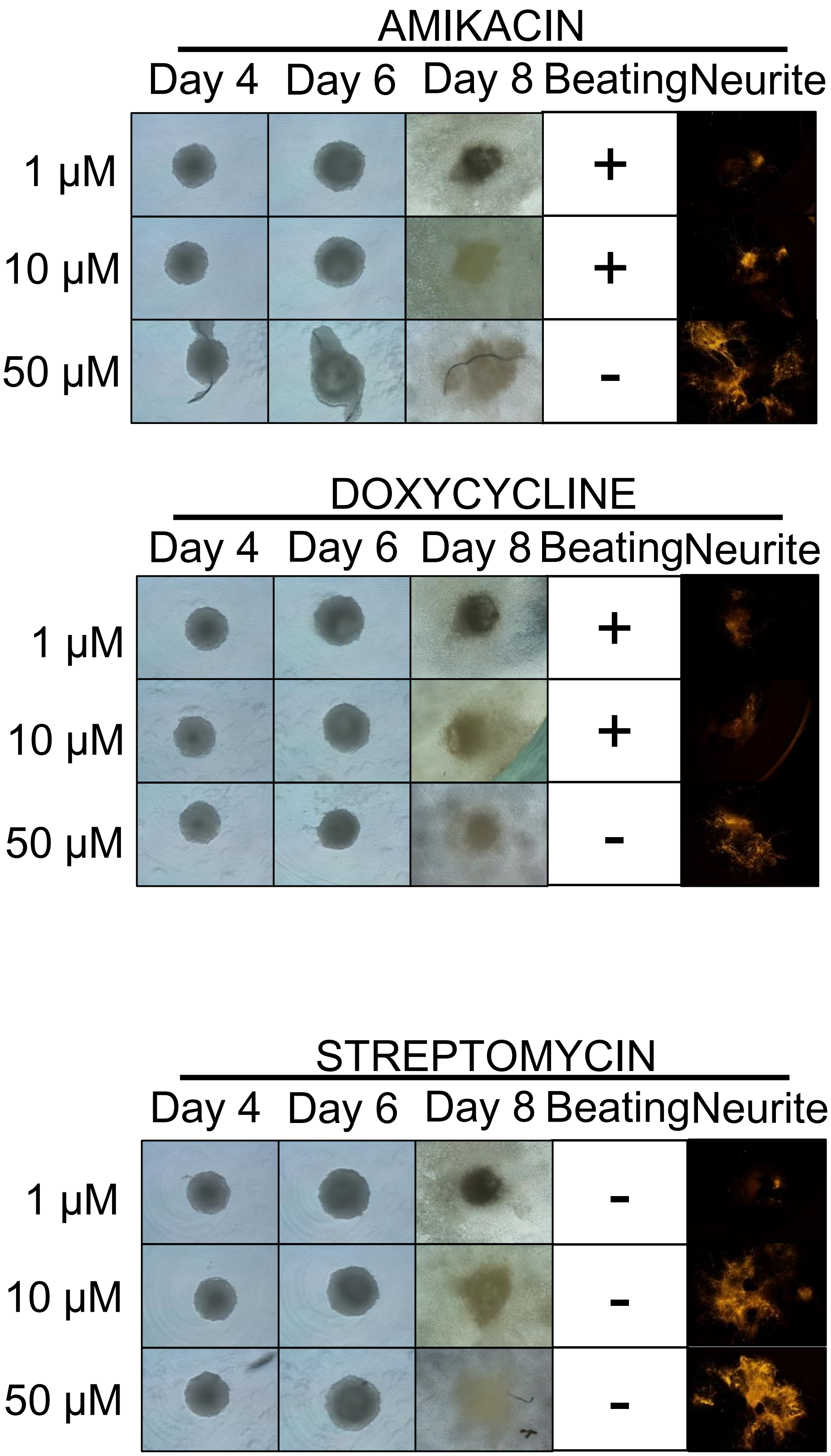

Yu.et al Fig. S1

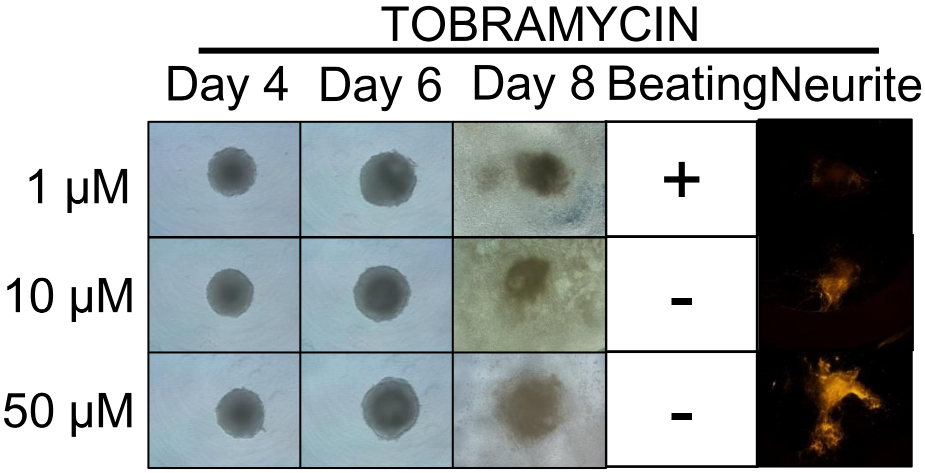

Yu.et al Fig. S1

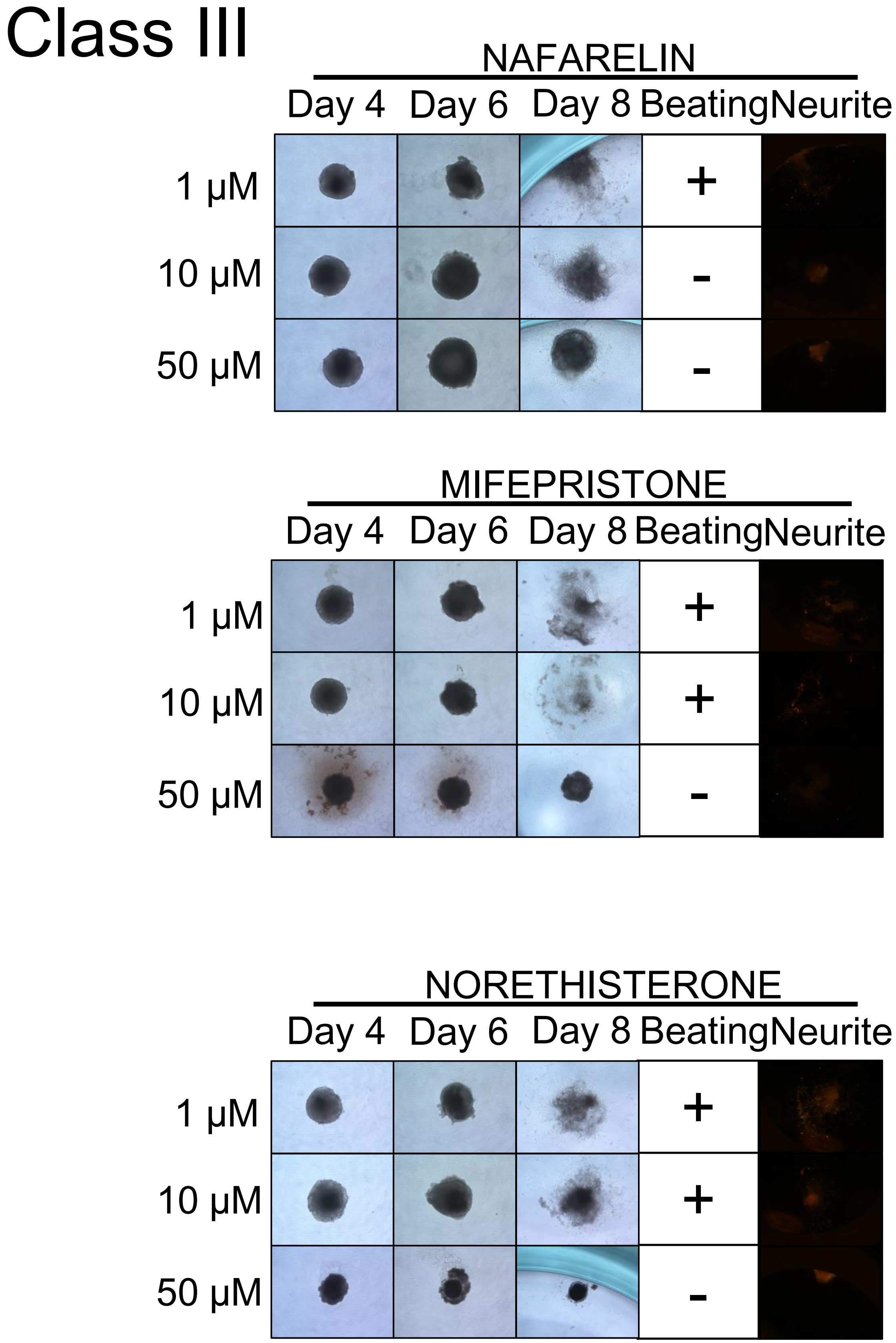

Yu.et al Fig. S1

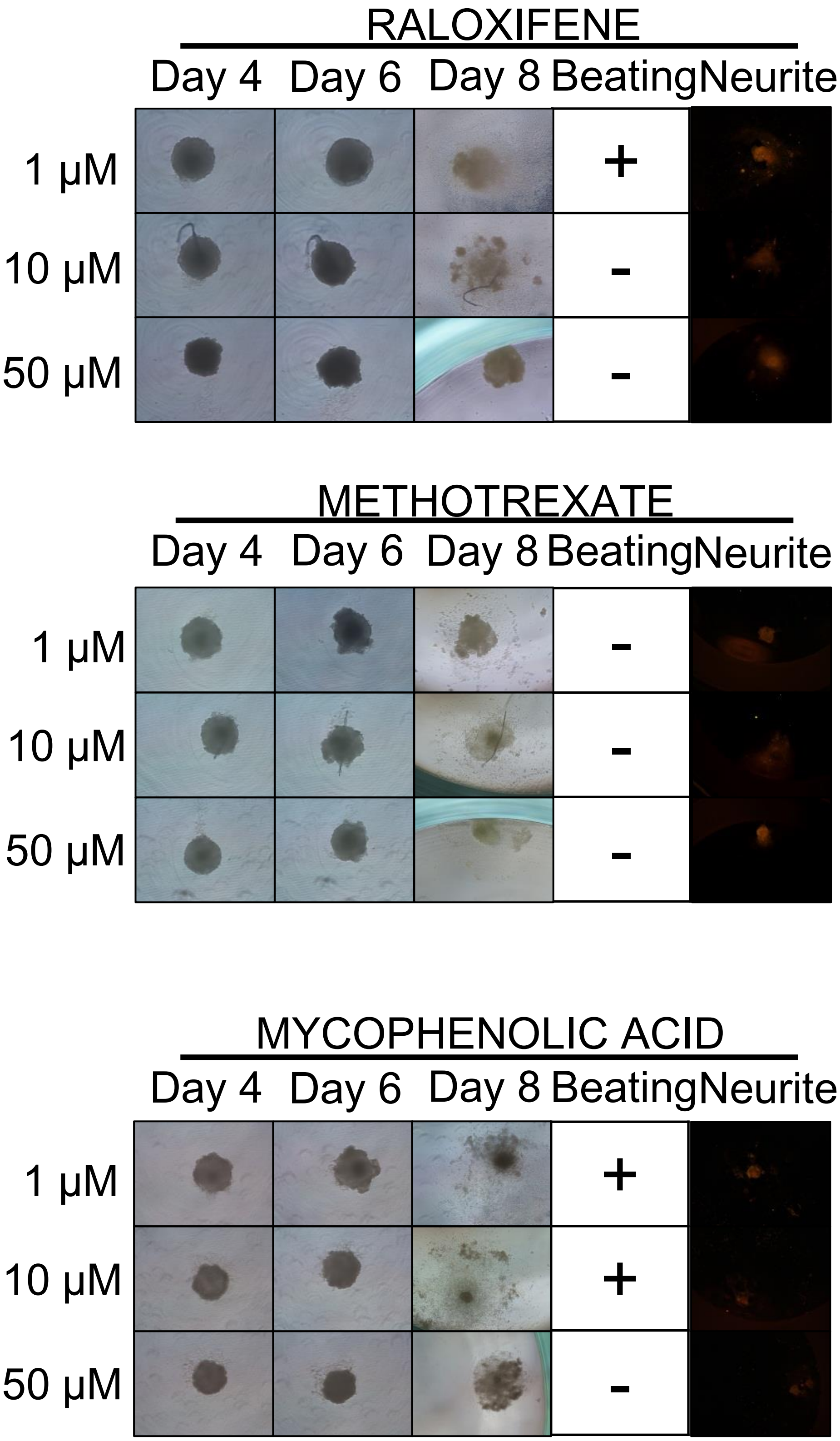

Yu.et al Fig. S1

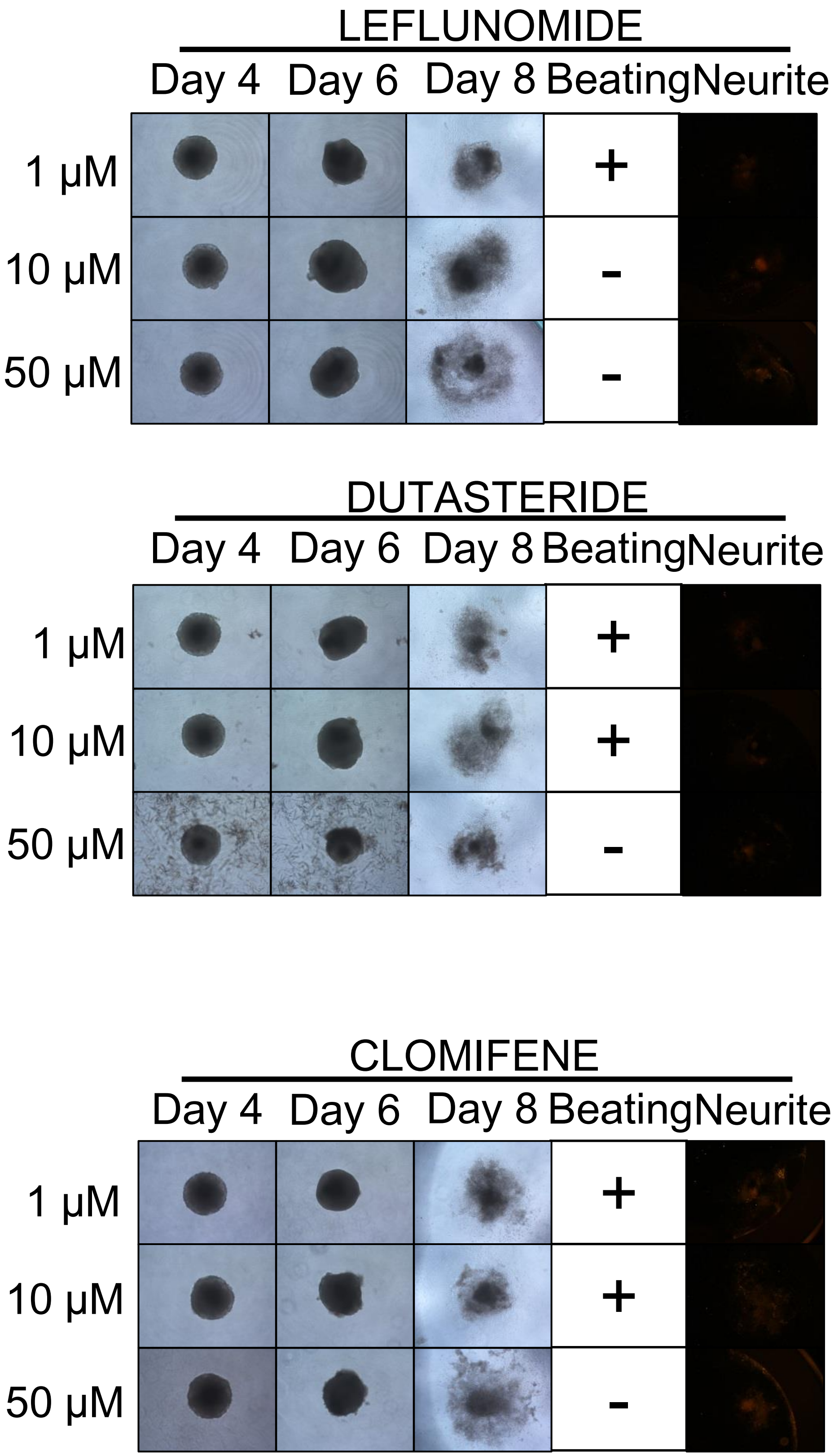

Yu.et al Fig. S1

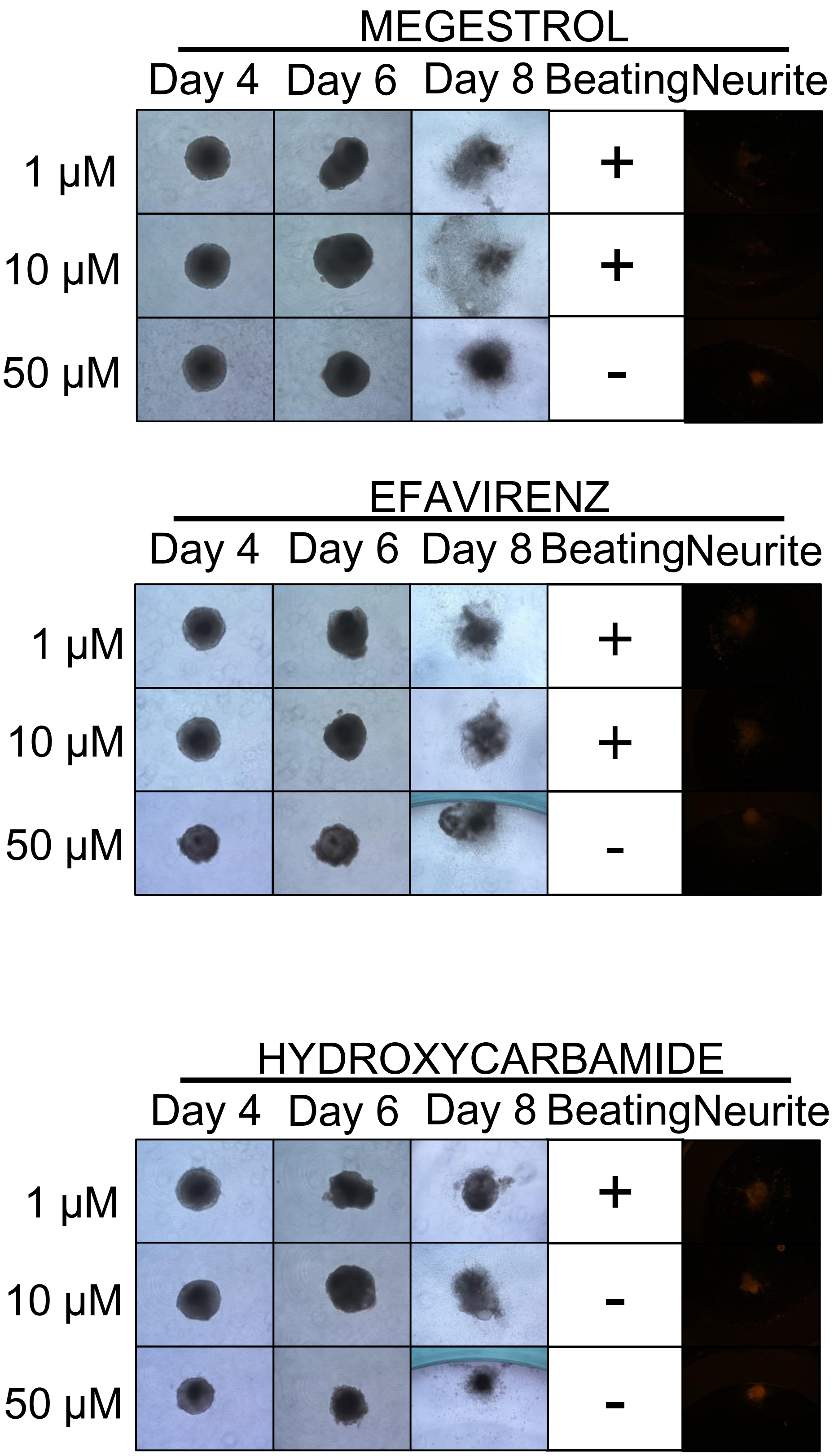

Yu.et al Fig. S1

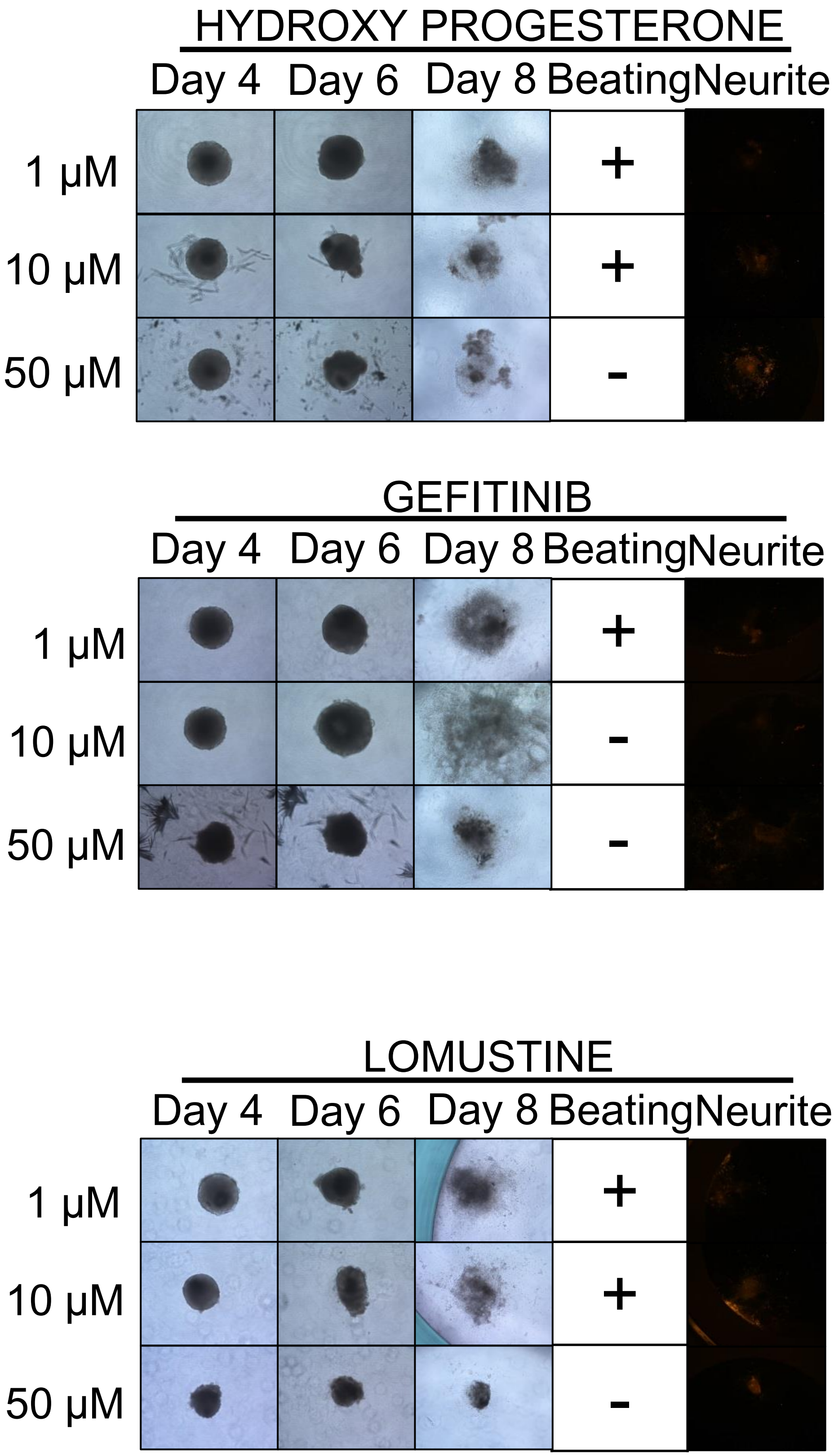

Yu.et al Fig. S1

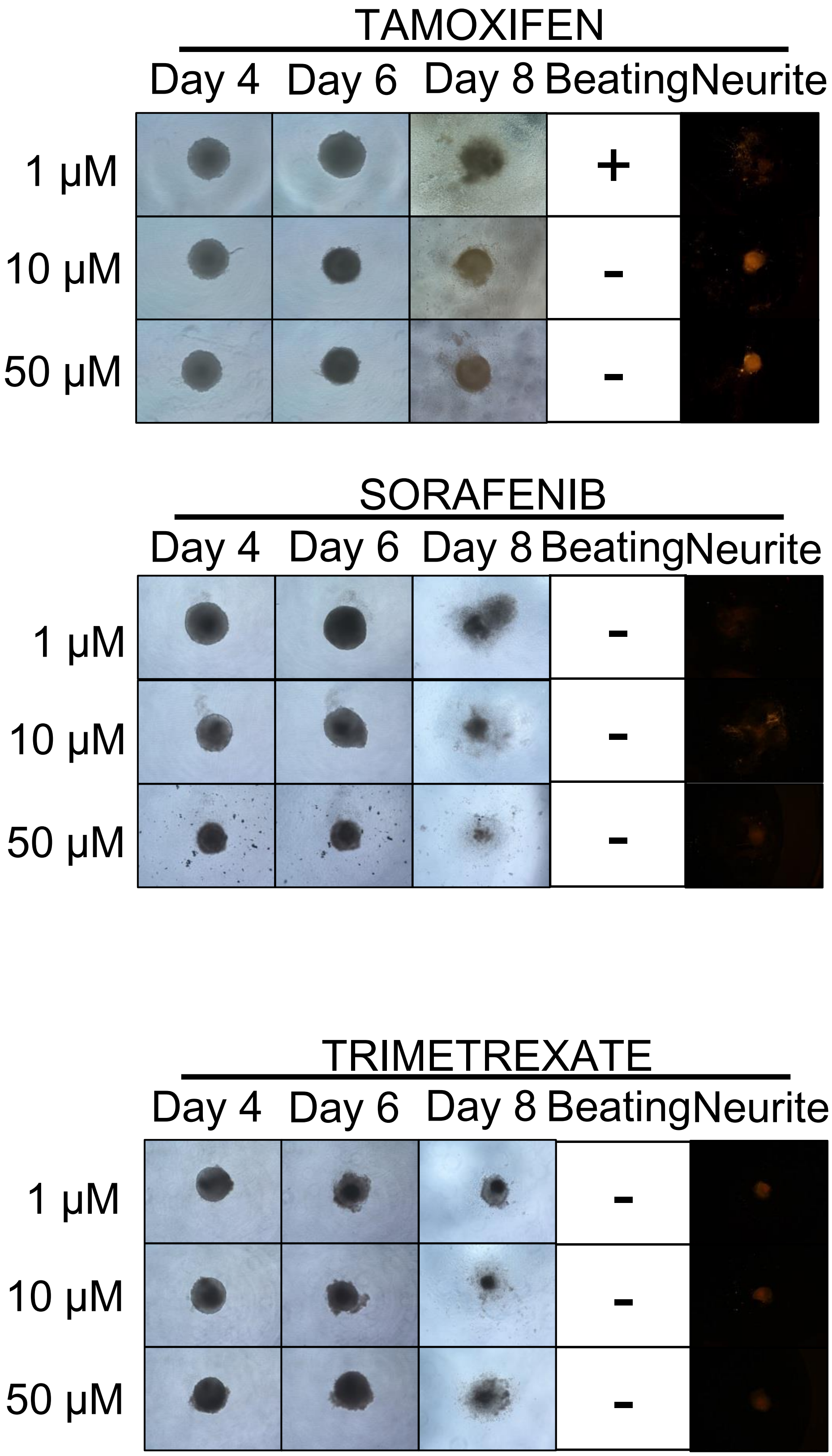

Yu.et al Fig. S1

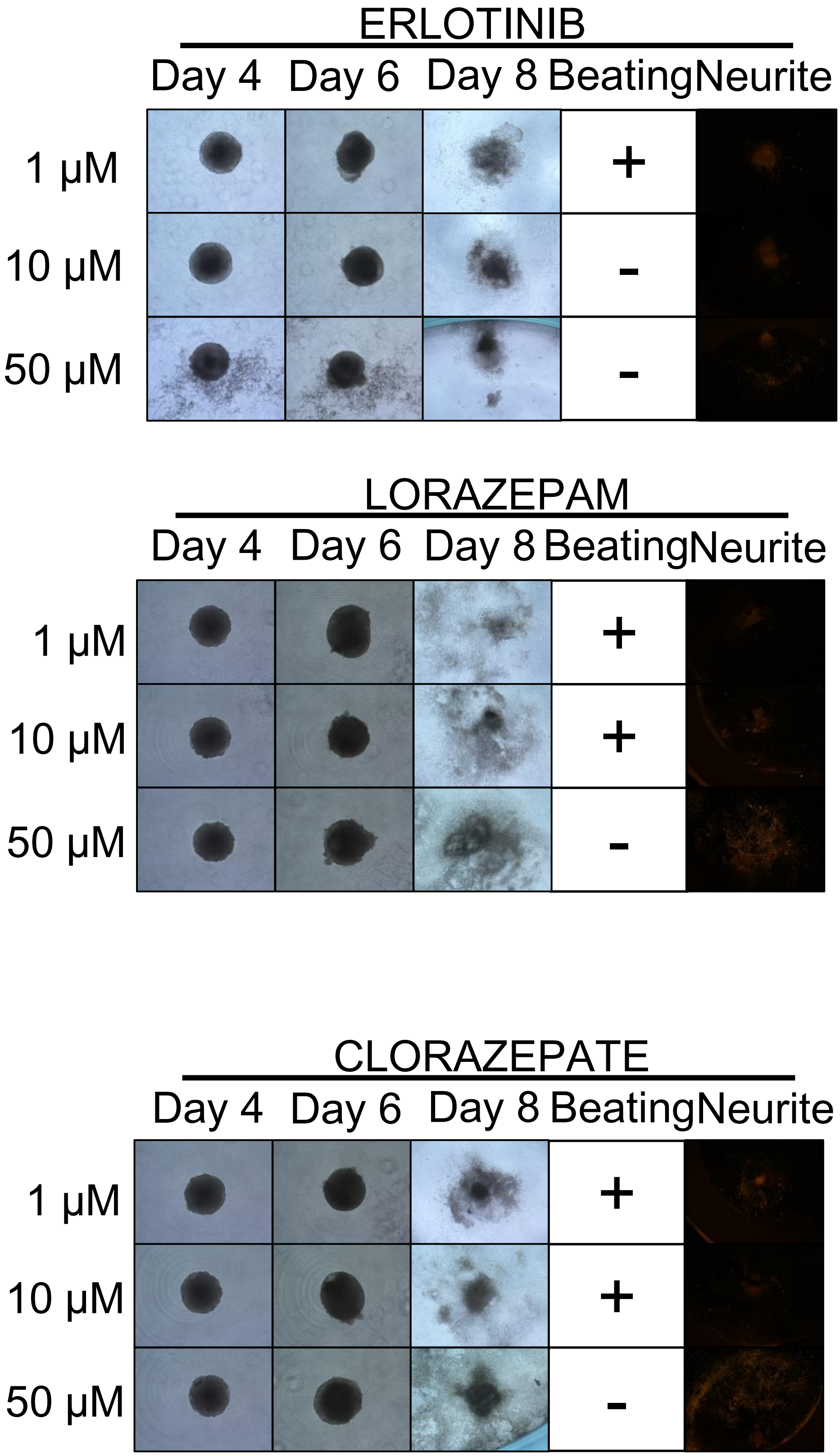

Yu.et al Fig. S1

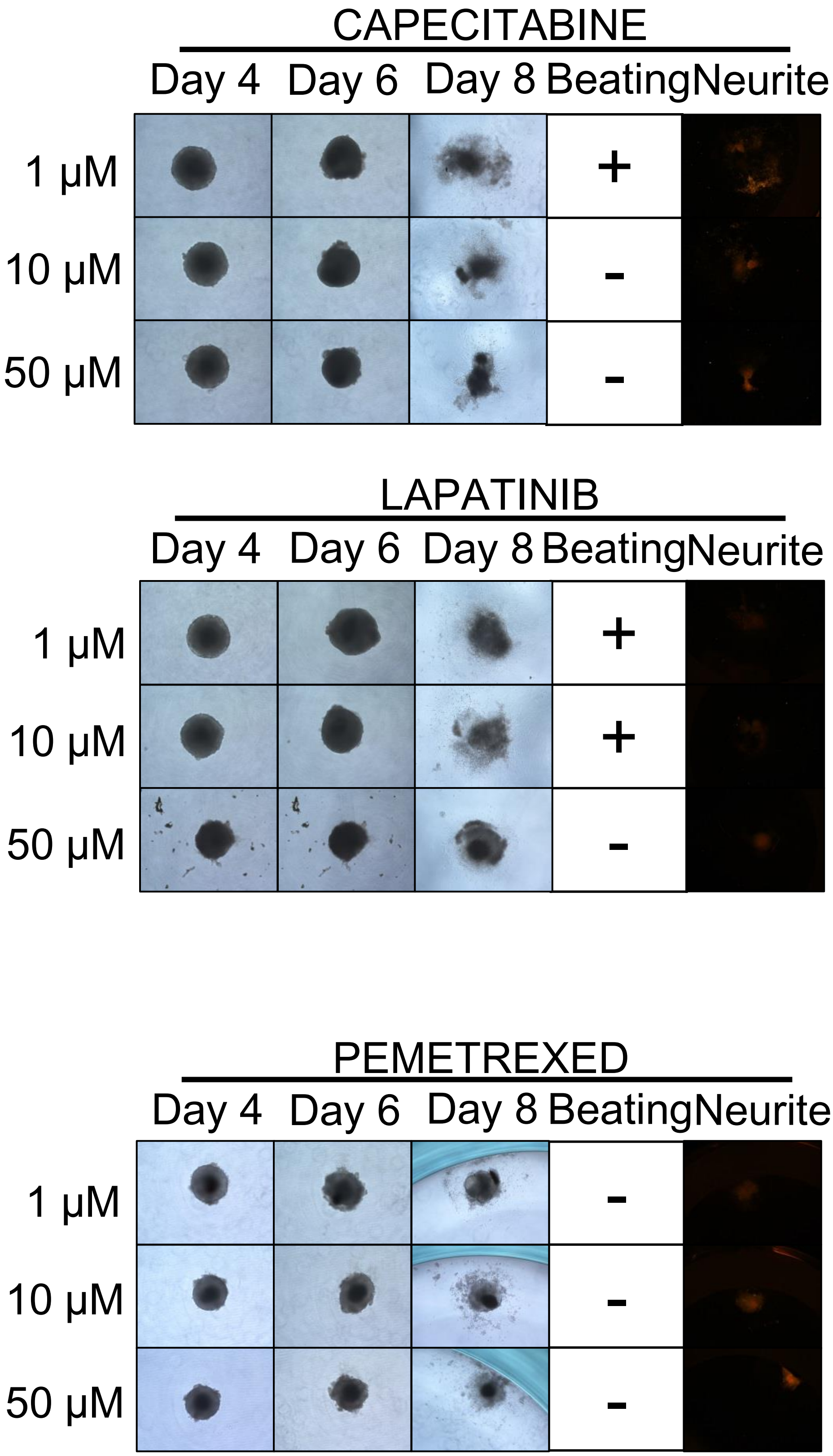

Yu.et al Fig. S1

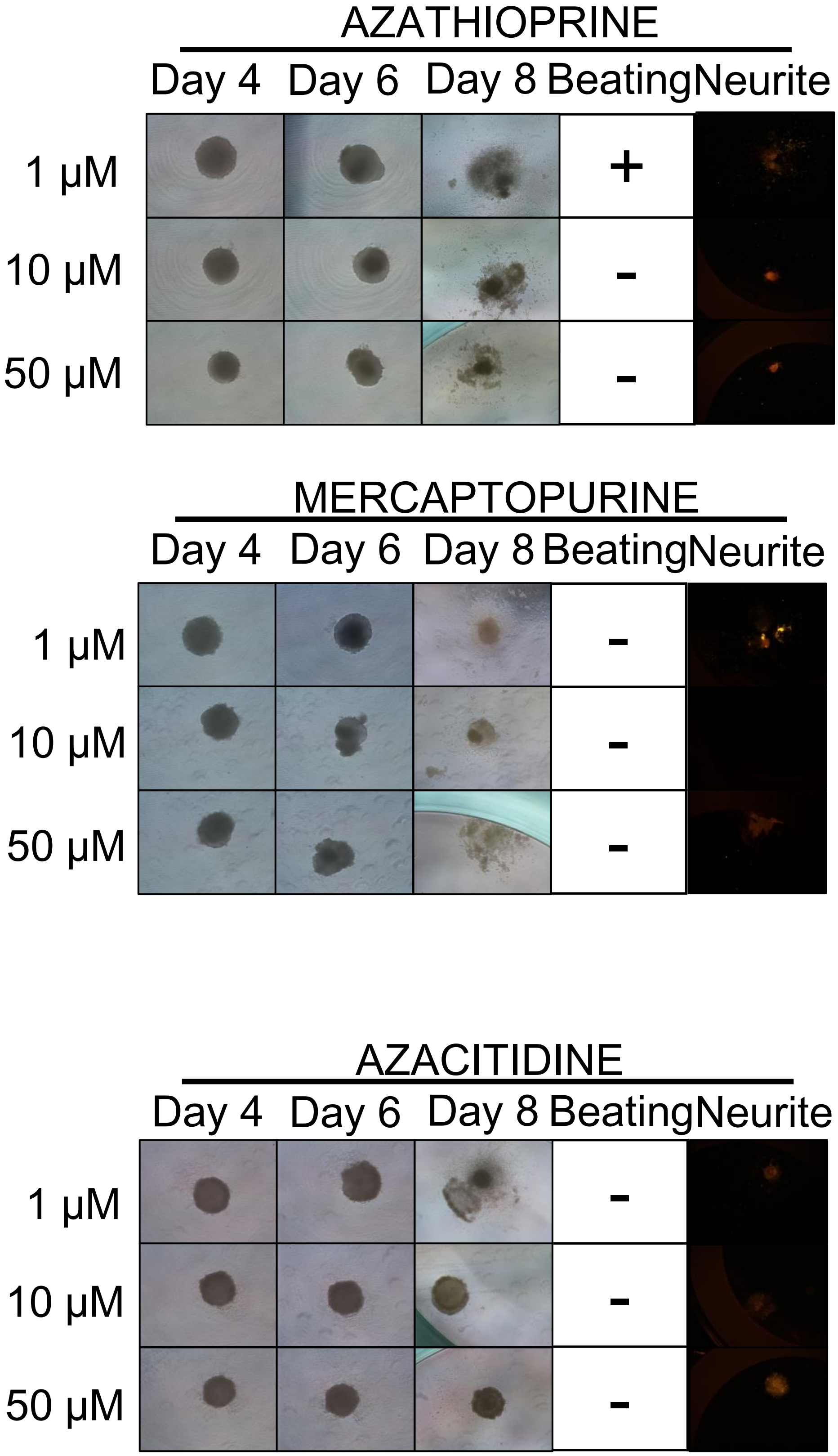

Yu.et al Fig. S1

Class IV

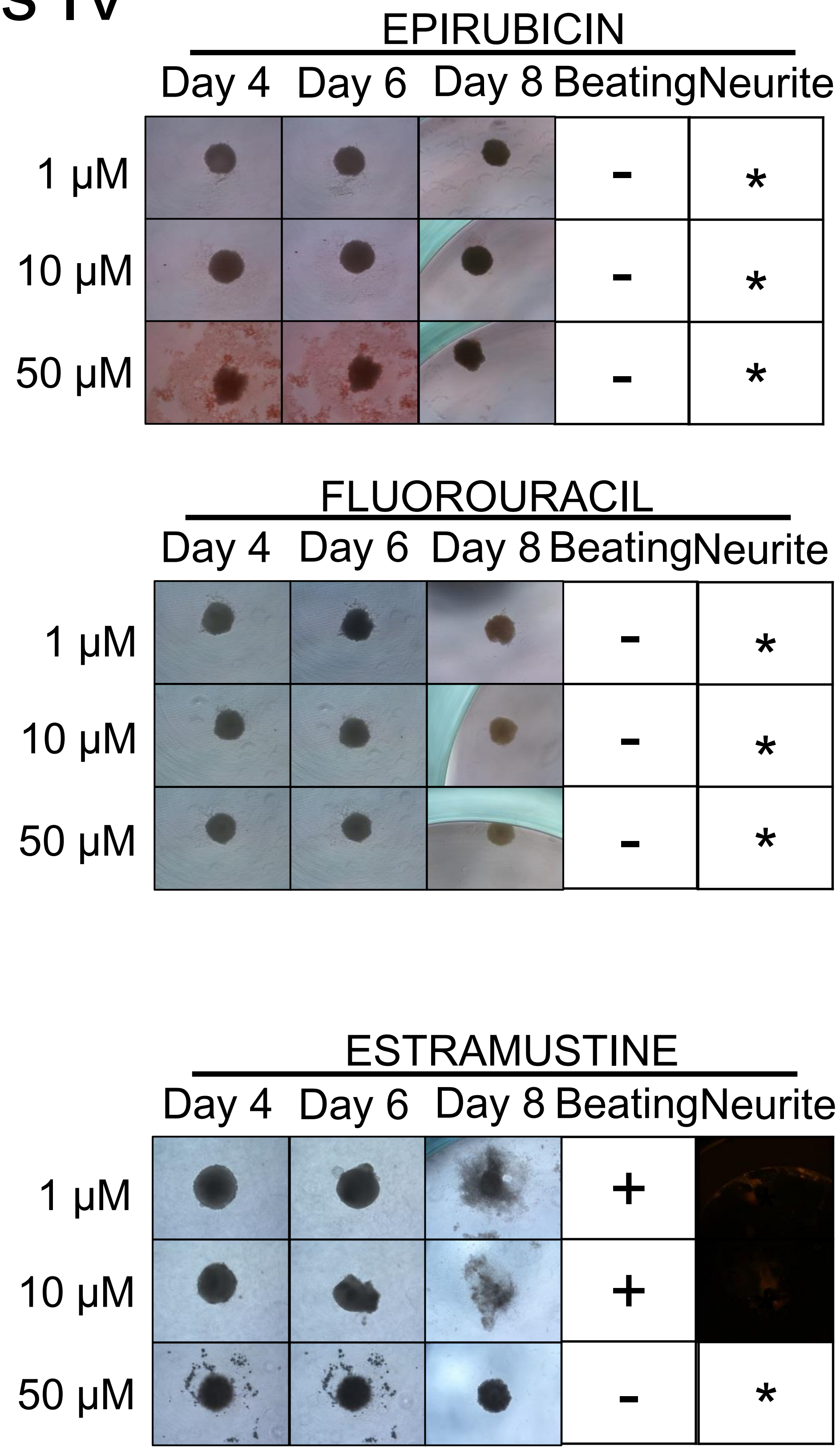

Yu.et al Fig. S1

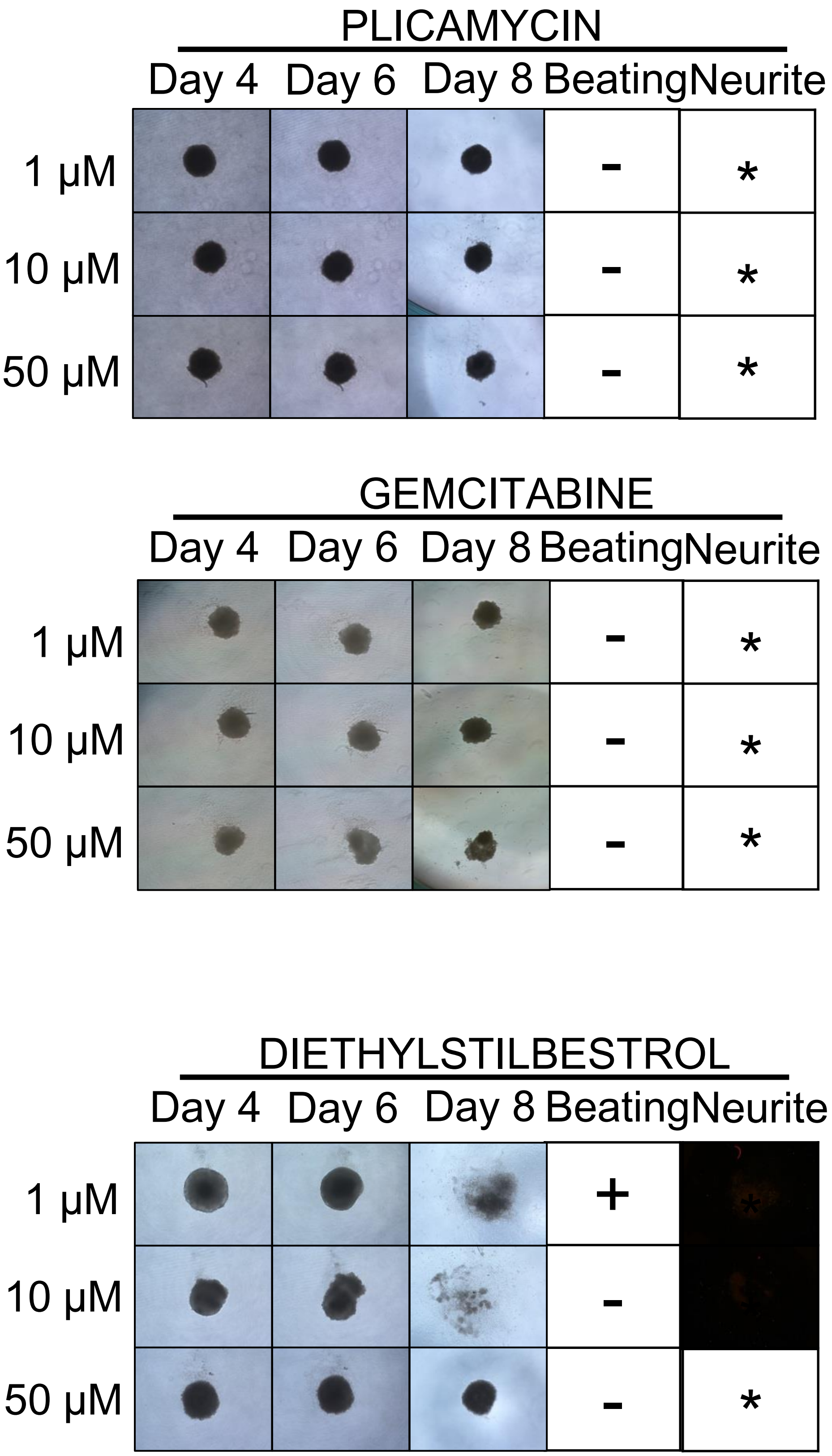

Yu.et al Fig. S1

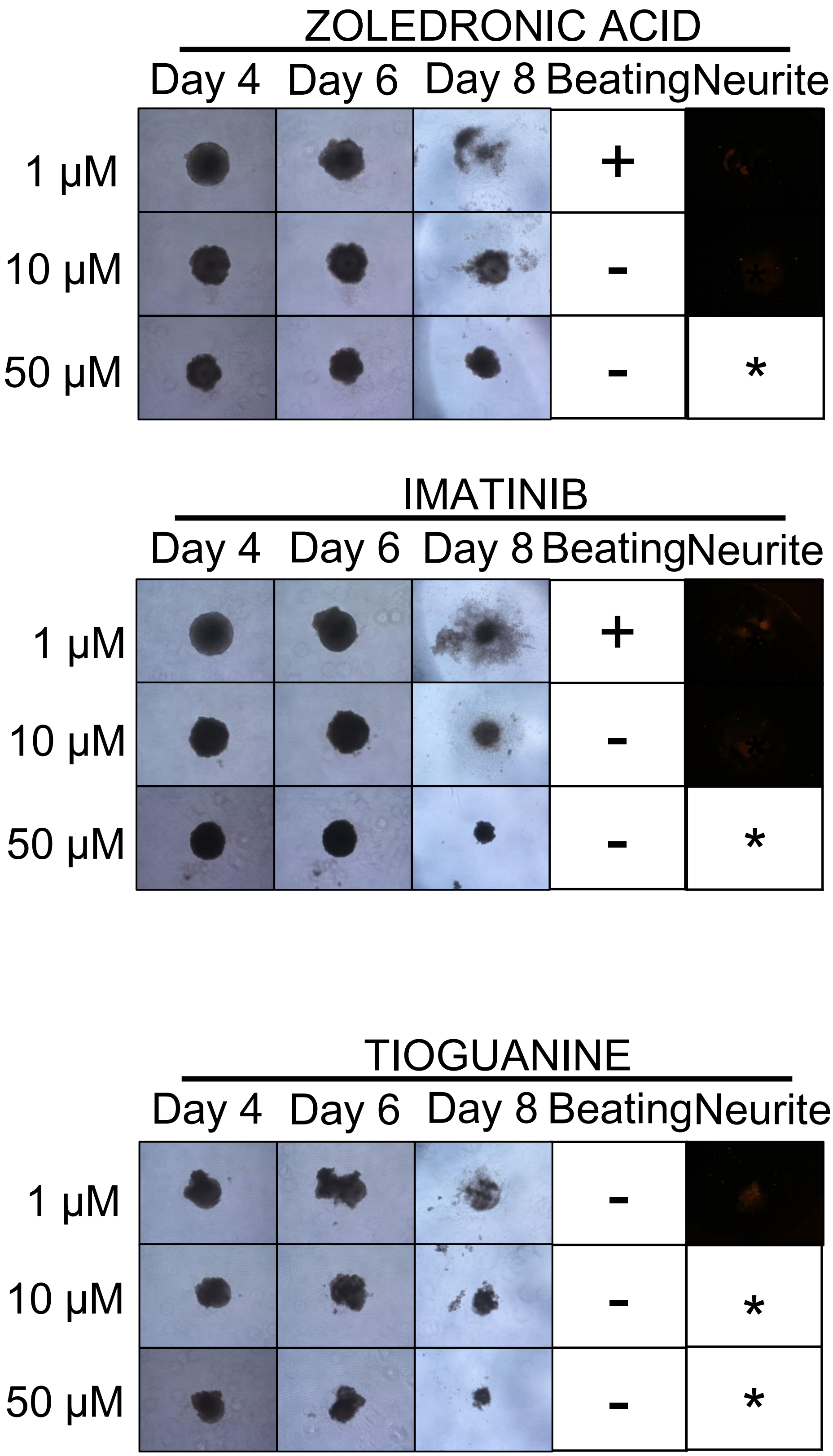

Yu.et al Fig. S1

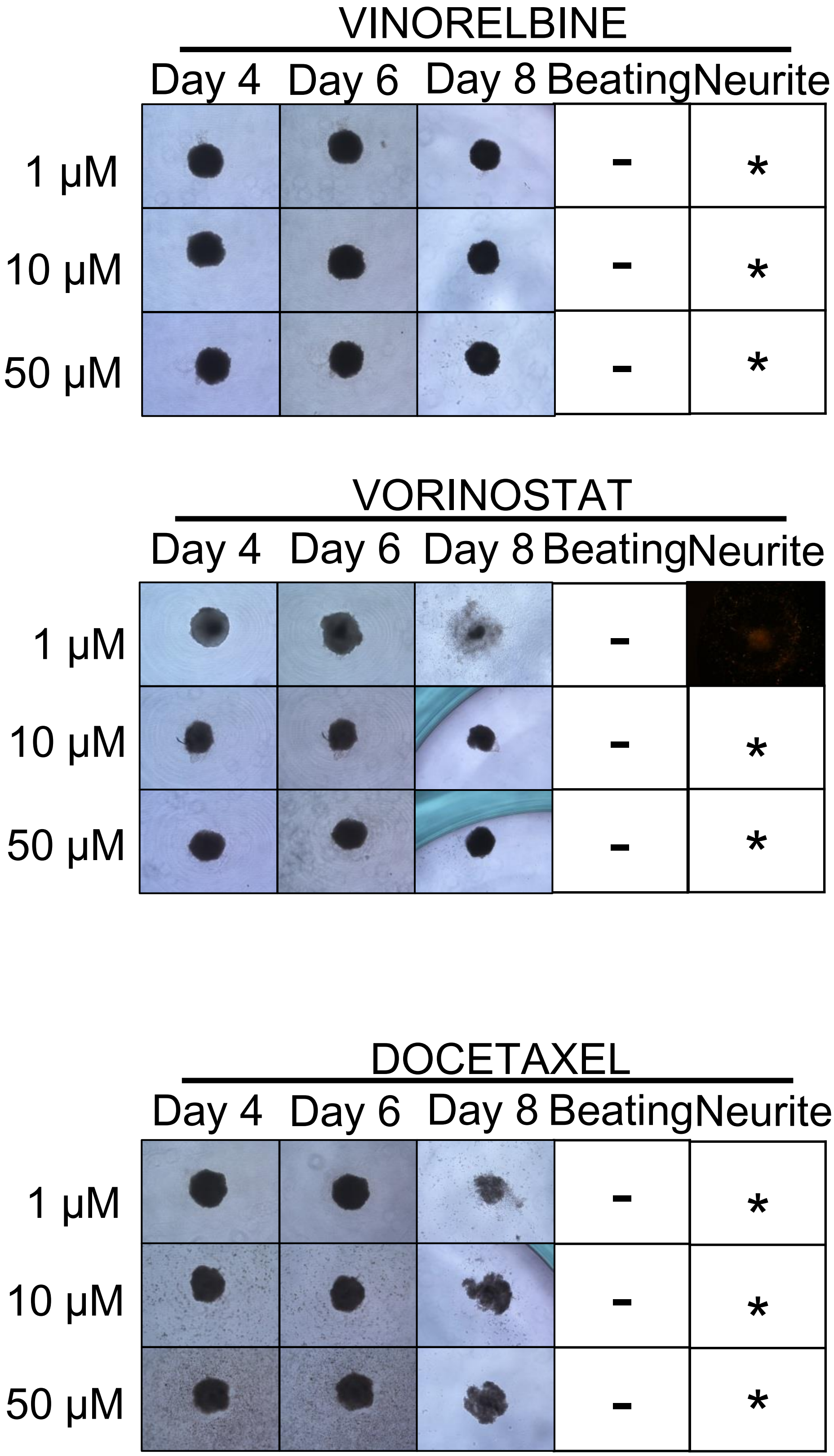

Yu.et al Fig. S1

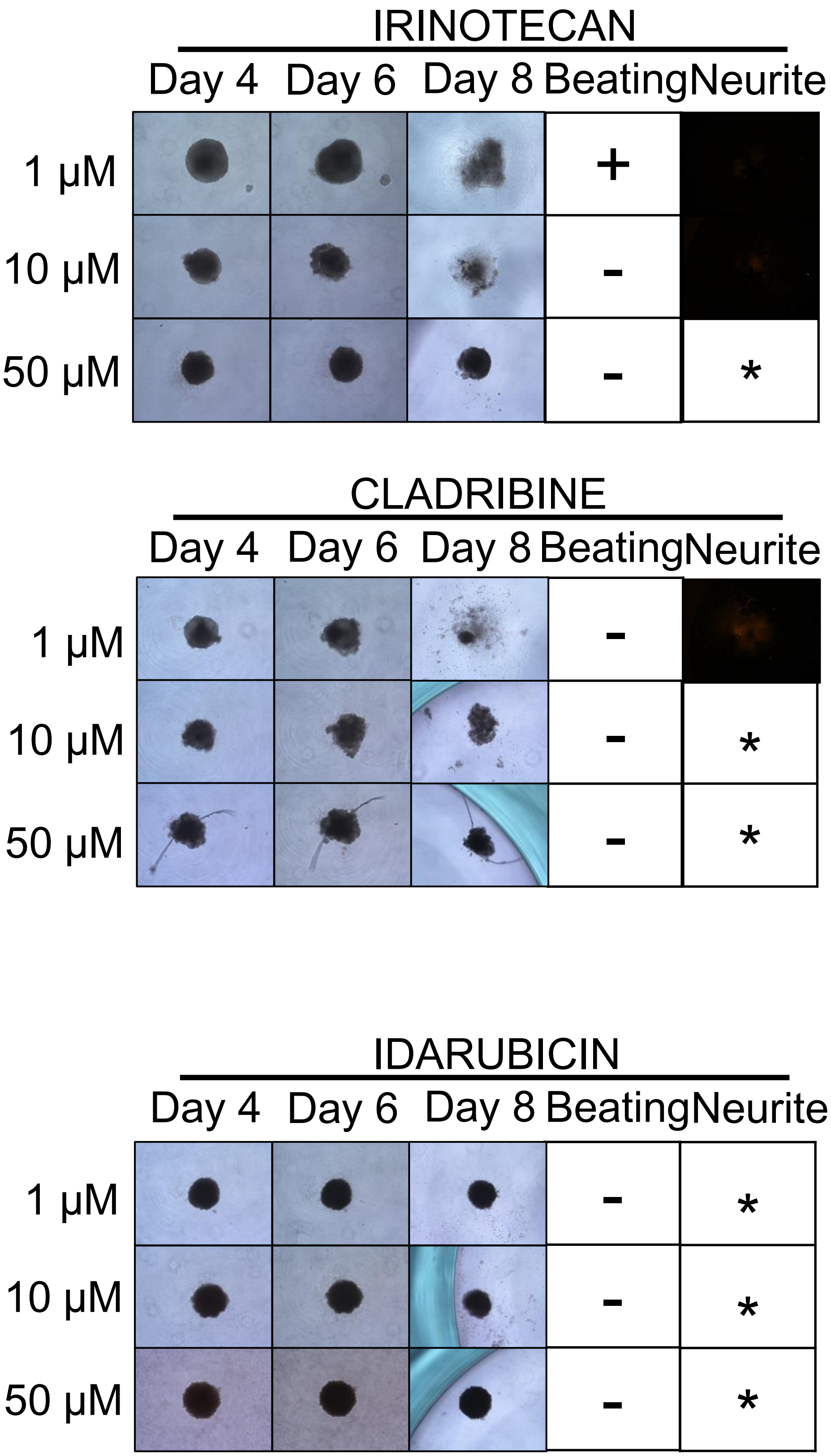

Yu.et al Fig. S1

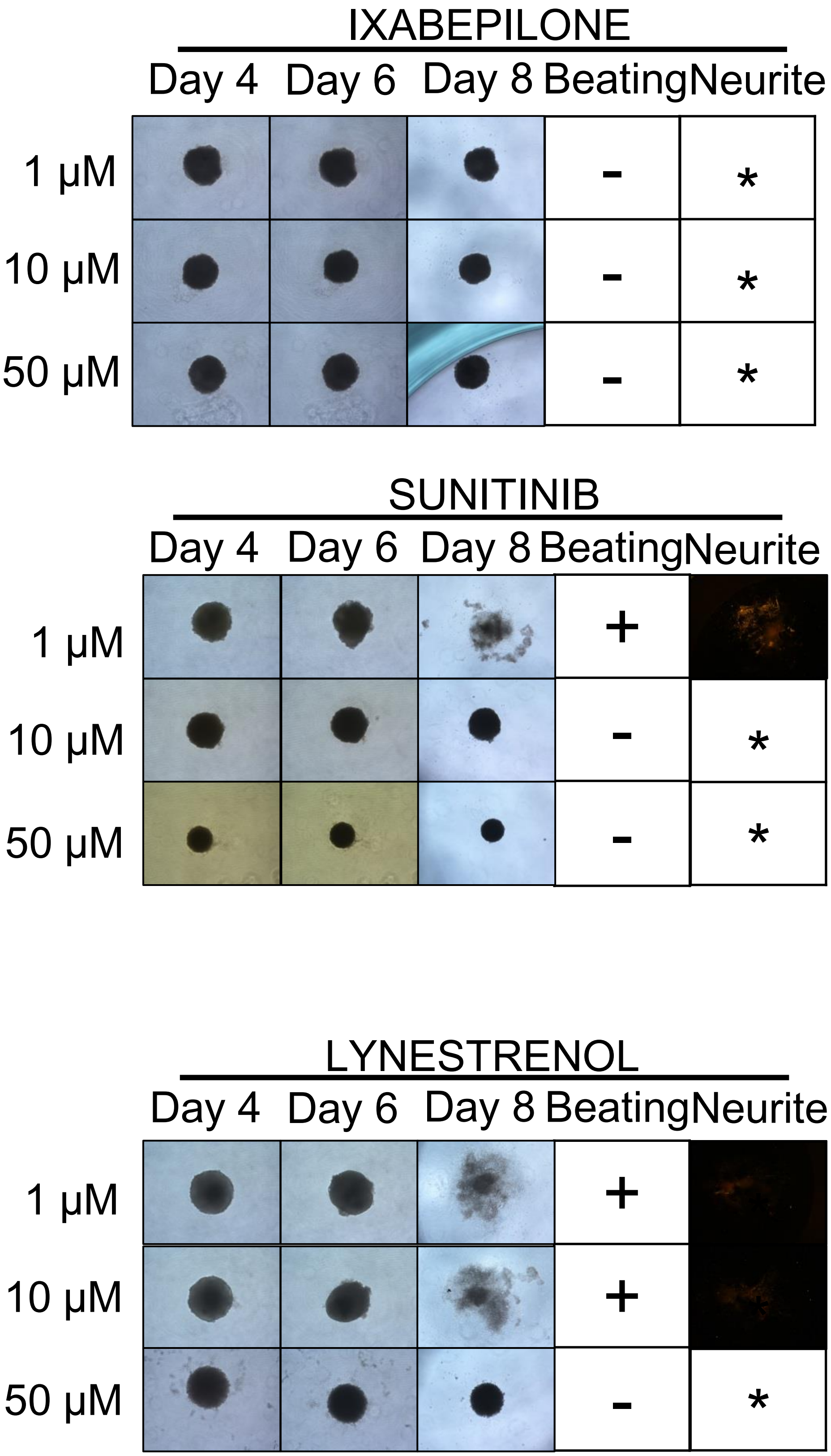

Yu.et al Fig. S1

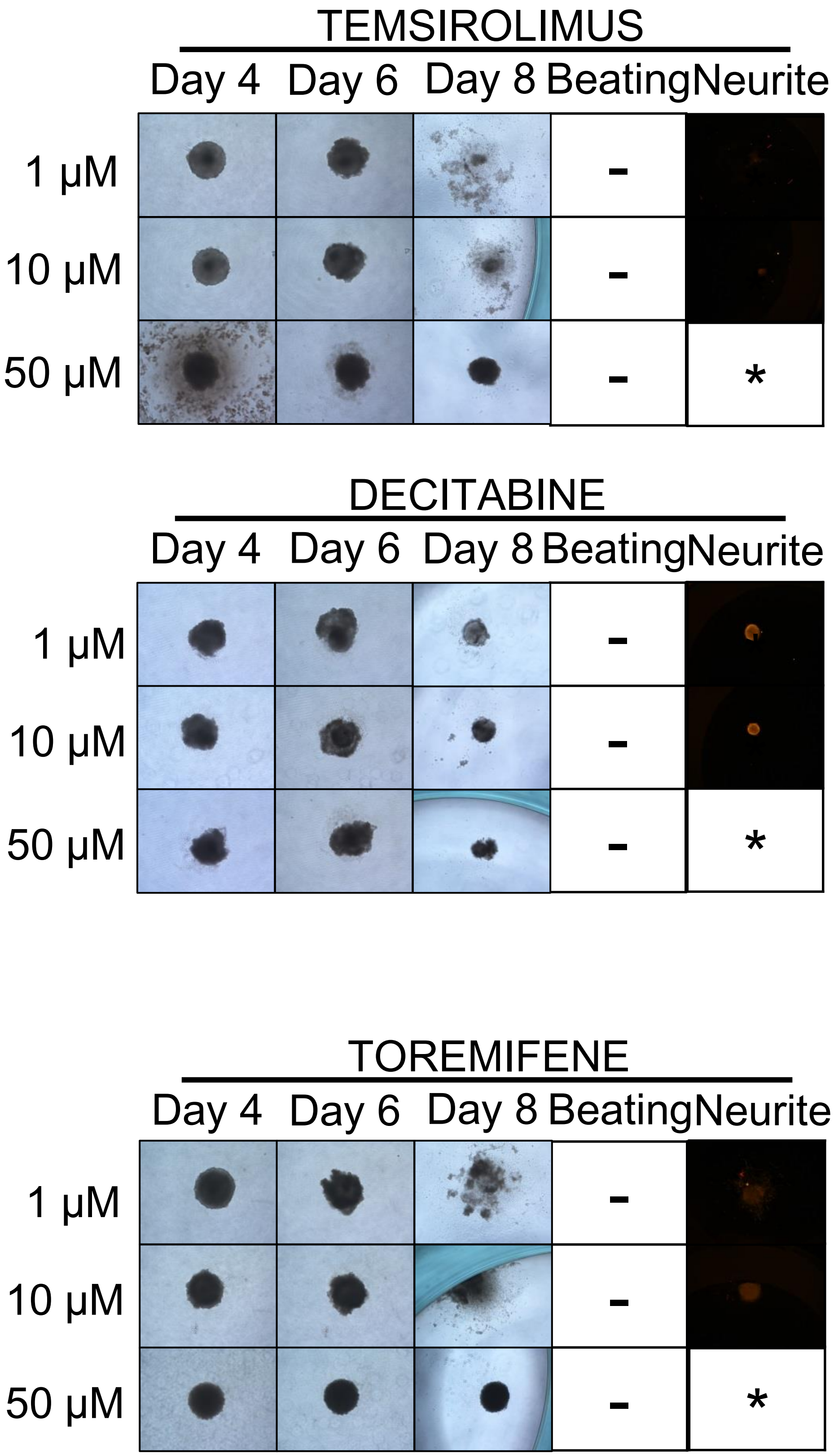

Yu.et al Fig. S1

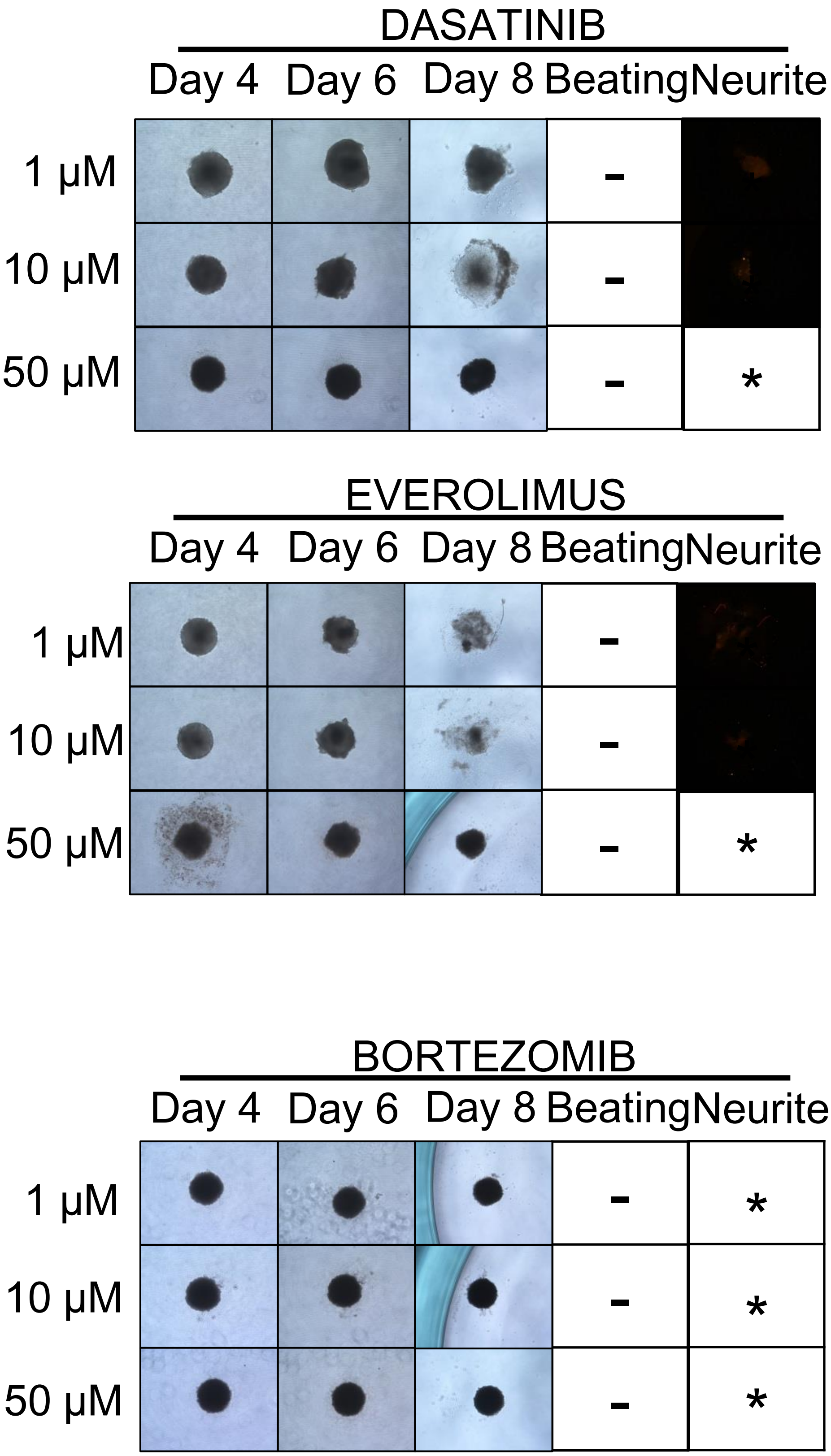

Yu.et al Fig. S1

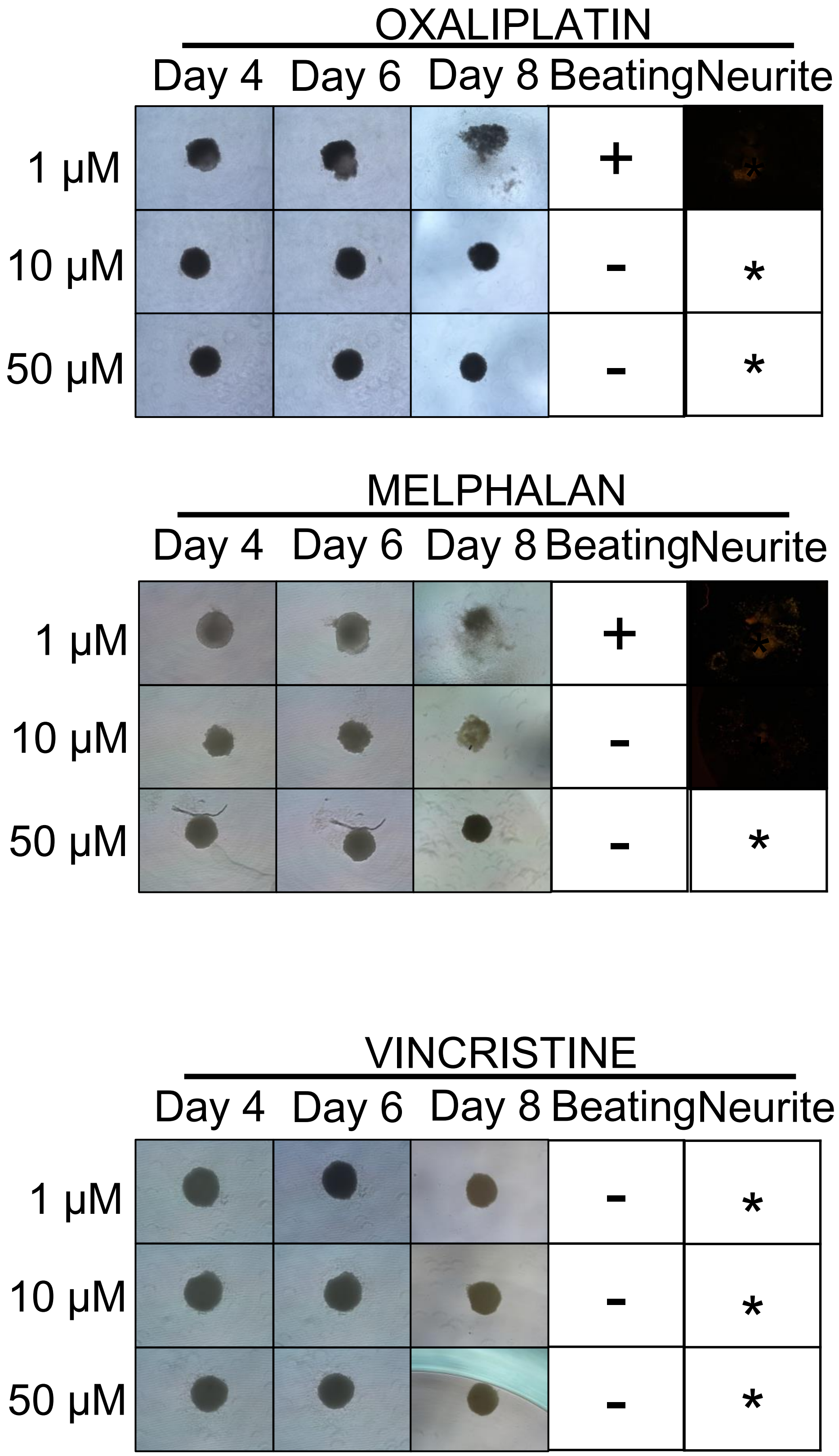

Yu.et al Fig. S1

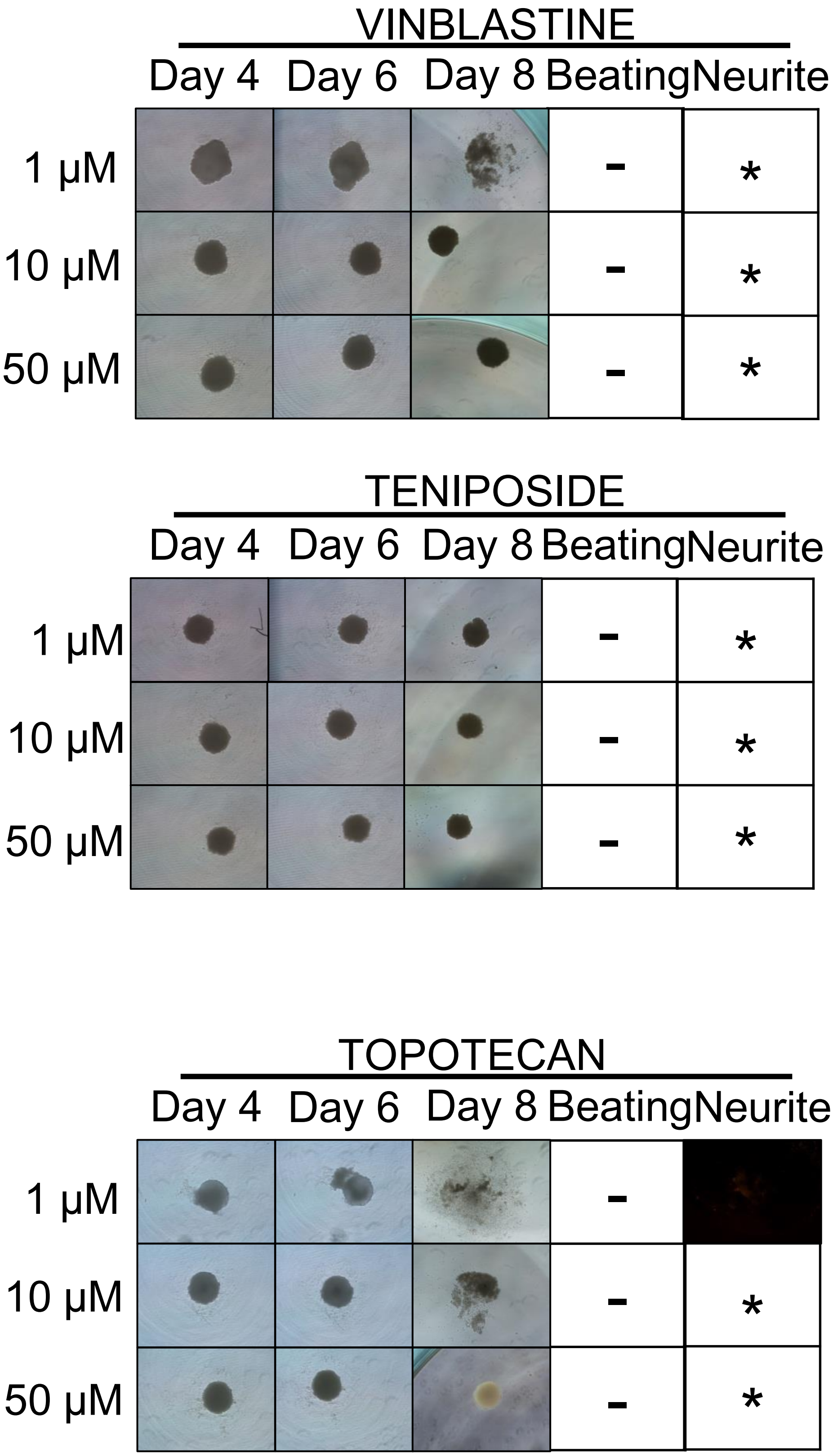

Yu.et al Fig. S1

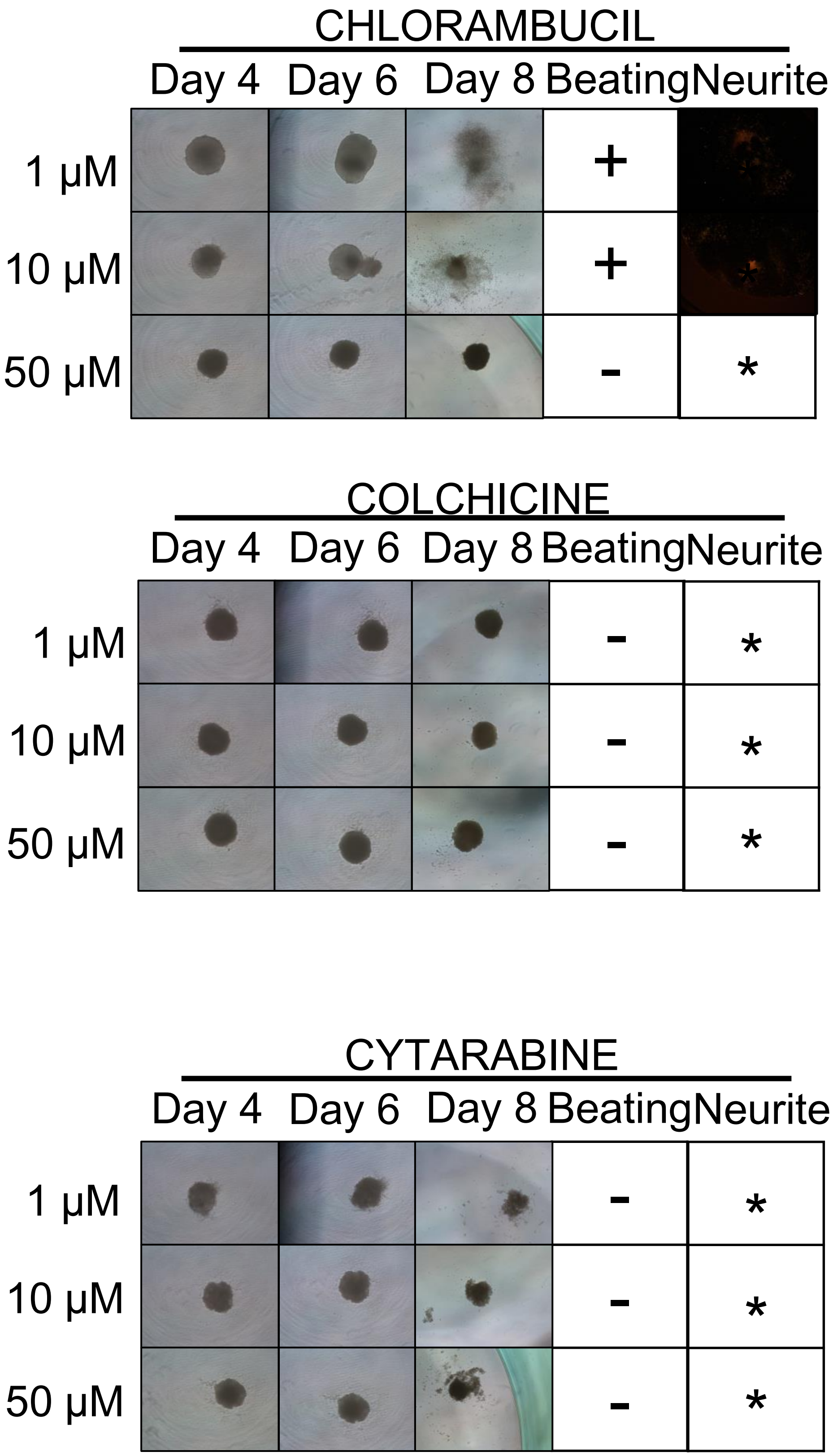

Yu.et al Fig. S1

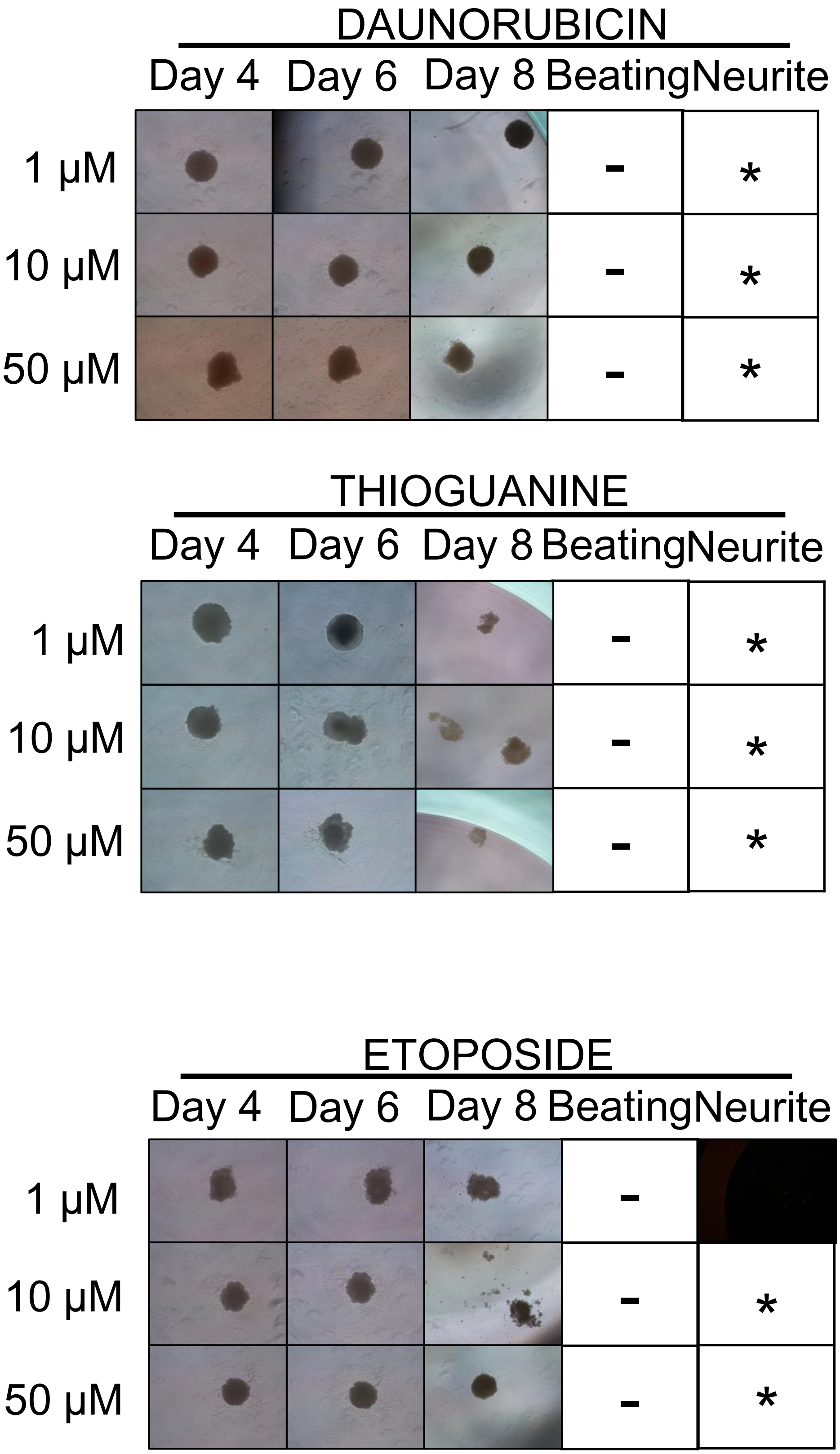

Yu.et al Fig. S1

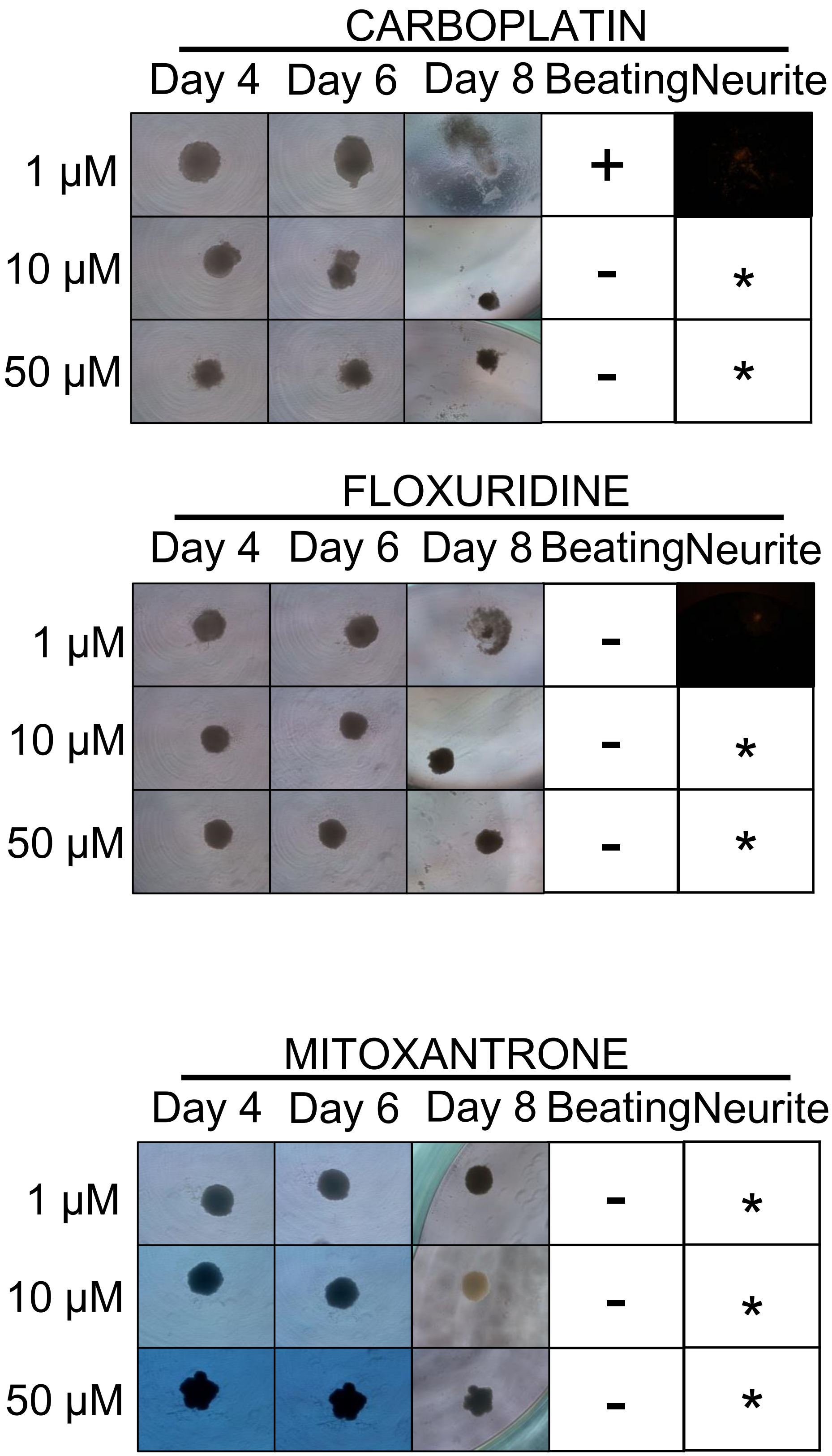

Yu.et al Fig. S1

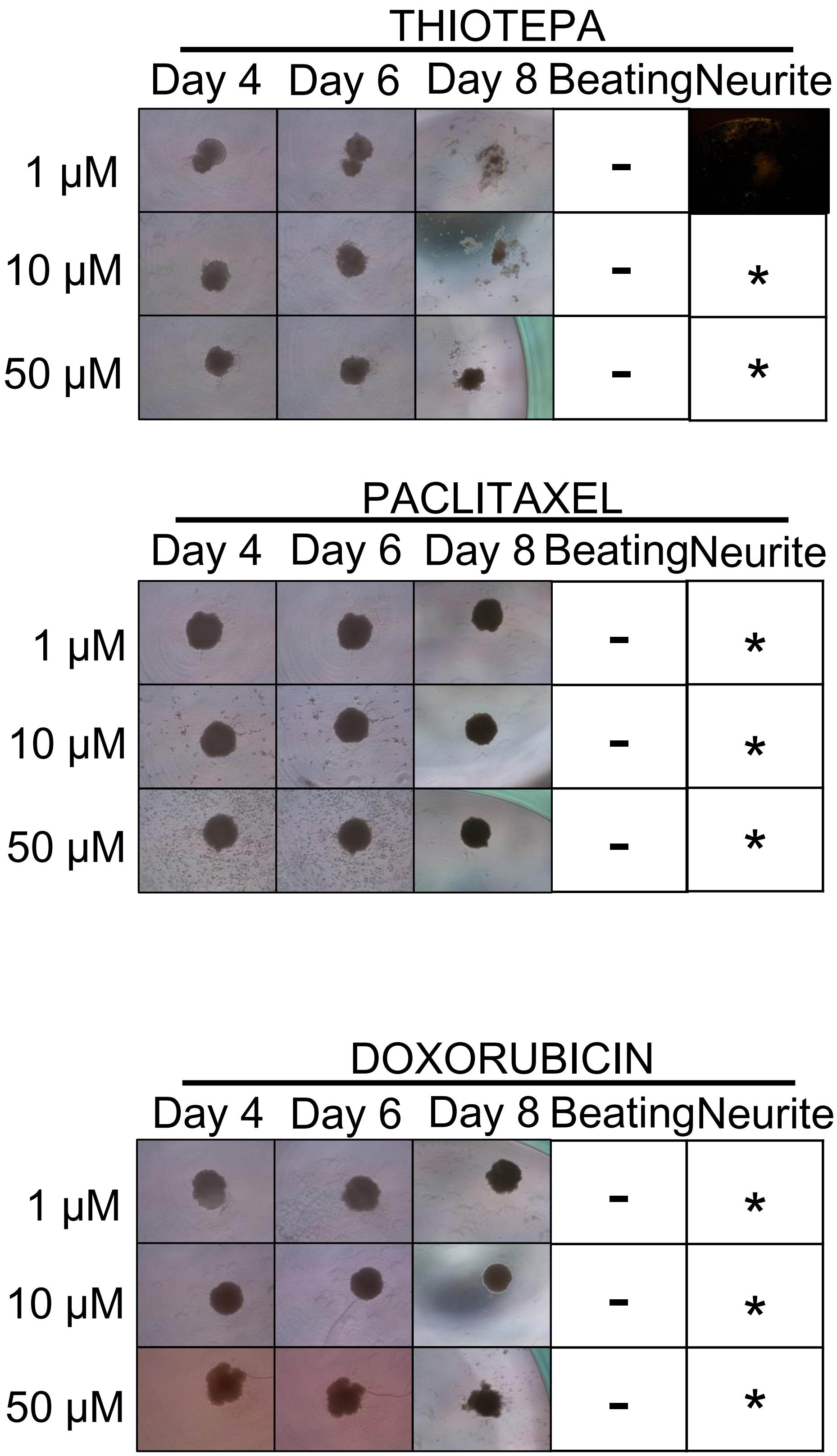

Yu.et al Fig. S1

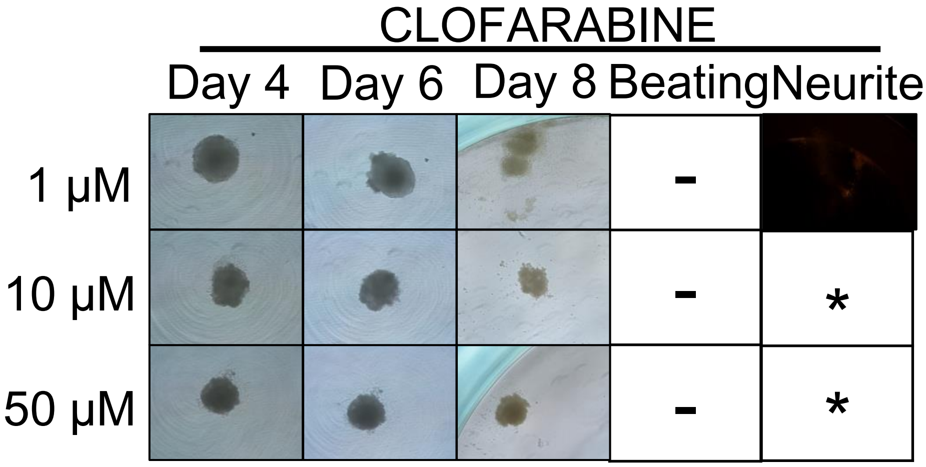

Yu.et al Fig. S1
