# Supplementary material for: A Modified Murine Embryonic Stem Cell Test for Evaluating the Teratogenic Effects of Drugs on Early Embryogenesis
Source: PLoS One. 2015 Dec 18;10(12):e0145286. doi: 10.1371/journal.pone.0145286 (PMC4686177; doi:10.1371/journal.pone.0145286)
Supplement: S1 Methods — (DOCX) [file pone.0145286.s002.docx]

**Methods S1**

**EST screening of Cat.A and Cat.B drugs**

Drugs chosen from FDA pregnancy categories A and B (see Table S3) were supplied as 10 mM DMSO stock solutions by the Chemical Biology Screening Center of Tokyo Medical and Dental University. Administration of these drugs to EBs was carried out as described in Materials and Methods.
